# Identifying MAGE-A4-positive tumors for TCR T cell therapies in HLA-A∗02-eligible patients

**DOI:** 10.1016/j.omtm.2024.101265

**Published:** 2024-05-14

**Authors:** Tianjiao Wang, Jean-Marc Navenot, Stavros Rafail, Cynthia Kurtis, Mark Carroll, Marian Van Kerckhoven, Sofie Van Rossom, Kelly Schats, Konstantinos Avraam, Robyn Broad, Karen Howe, Ashley Liddle, Amber Clayton, Ruoxi Wang, Laura Quinn, Joseph P. Sanderson, Cheryl McAlpine, Carly Carozza, Eric Pimpinella, Susan Hsu, Francine Brophy, Erica Elefant, Paige Bayer, Dennis Williams, Marcus O. Butler, Jeffrey M. Clarke, Justin F. Gainor, Ramaswamy Govindan, Victor Moreno, Melissa Johnson, Janet Tu, David S. Hong, George R. Blumenschein

**Affiliations:** 1 Clinical Biomarkers & Companion Diagnostics, Adaptimmune, Philadelphia, PA, USA; 2 Biomarker Discovery and Platform, Adaptimmune, Philadelphia, PA, USA; 3 Information Management Clinical Systems, Adaptimmune, Philadelphia, PA, USA; 4 Assay Development Histopathology, CellCarta, Antwerpen, Belgium; 5 Histopathology & Image Quantification Unit, CellCarta, Antwerpen, Belgium; 6 Translational Sciences, Adaptimmune, Abingdon, Oxfordshire, UK; 7 Target Validation, Adaptimmune, Abingdon, Oxfordshire, UK; 8 Preclinical Research, Adaptimmune, Abingdon, Oxfordshire, UK; 9 Histocompatibility Laboratory Services, American Red Cross, Philadelphia, PA, USA; 10 Clinical Science, Adaptimmune, Philadelphia, PA, USA; 11 Late Stage Development, Adaptimmune, Philadelphia, PA, USA; 12 Department of Medical Oncology and Hematology, Princess Margaret Cancer Centre, Departments of Immunology and Medicine, University of Toronto, Toronto, ON, Canada; 13 Department of Medicine, Duke Cancer Institute, Durham, NC, USA; 14 Department of Medicine, Massachusetts General Hospital, Boston, MA, USA; 15 Department of Medicine, Washington University School of Medicine, St. Louis, MO, USA; 16 Oncology, START Madrid FJD, Hospital Universitario Fundación Jiménez Díaz, Madrid, Spain; 17 Lung Cancer Research and Drug Development, Sarah Cannon Research Institute, Nashville, TN, USA; 18 Department of General Oncology, The University of Texas MD Anderson Cancer Center, Houston, TX, USA; 19 Department of Investigational Cancer Therapeutics, The University of Texas MD Anderson Cancer Center, Houston, TX, USA; 20 Thoracic/Head and Neck Medical Oncology, The University of Texas MD Anderson Cancer Center, Houston, TX, USA

**Keywords:** HLA, immunotherapy, MAGE-A4, T cell receptor, T cell therapy, biomarker, assay validation, precision medicine, patient screening, clinical trial

## Abstract

T cell receptor (TCR) T cell therapies target tumor antigens in a human leukocyte antigen (HLA)-restricted manner. Biomarker-defined therapies require validation of assays suitable for determination of patient eligibility. For clinical trials evaluating TCR T cell therapies targeting melanoma-associated antigen A4 (MAGE-A4), screening in studies NCT02636855 and NCT04044768 assesses patient eligibility based on: (1) high-resolution HLA typing and (2) tumor MAGE-A4 testing via an immunohistochemical assay in HLA-eligible patients. The HLA/MAGE-A4 assays validation, biomarker data, and their relationship to covariates (demographics, cancer type, histopathology, tissue location) are reported here. HLA-A∗02 eligibility was 44.8% (2,959/6,606) in patients from 43 sites across North America and Europe. While HLA-A∗02:01 was the most frequent HLA-A∗02 allele, others (A∗02:02, A∗02:03, A∗02:06) considerably increased HLA eligibility in Hispanic, Black, and Asian populations. Overall, MAGE-A4 prevalence based on clinical trial enrollment was 26% (447/1,750) across 10 solid tumor types, and was highest in synovial sarcoma (70%) and lowest in gastric cancer (9%). The covariates were generally not associated with MAGE-A4 expression, except for patient age in ovarian cancer and histology in non-small cell lung cancer. This report shows the eligibility rate from biomarker screening for TCR T cell therapies and provides epidemiological data for future clinical development of MAGE-A4-targeted therapies.

## Graphical abstract

**Figure:**
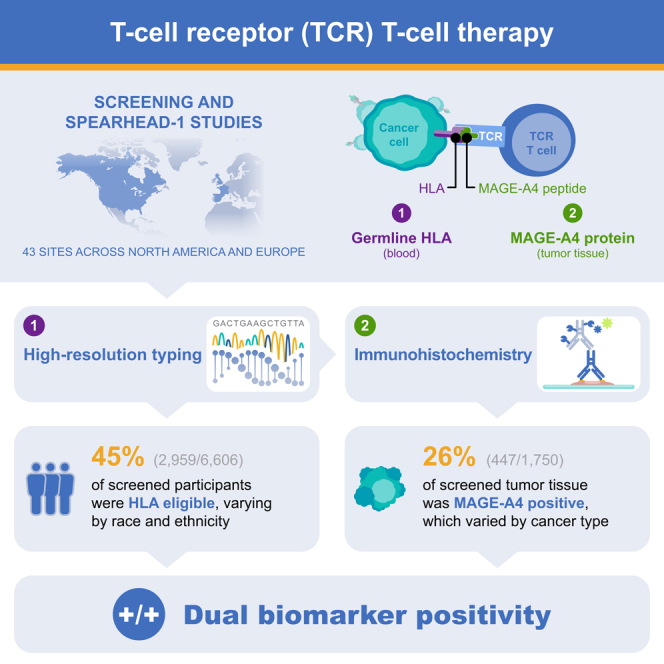

## Teaser

Wang and colleagues report HLA-A genotype and MAGE-A4 tumor expression, the combined key biomarkers required to determine eligibility for clinical trials assessing the clinical efficacy of MAGE-A4-specific TCR T cell therapies. The findings may guide future clinical development based on prevalence of these biomarkers in different populations and indications.

## Introduction

Adoptive cell therapies (ACTs) have improved patient outcomes in various therapeutic settings by employing activated lymphocytes to elicit anti-tumor effects^1^^,^^2^^,^^3^^,^^4^^,^^5^; however, ACT success largely depends on tumor characteristics. For metastatic solid tumors, T cell receptor (TCR) T cell therapies may overcome limitations of other ACTs, such as narrow applicability and/or decreased potential to activate the immune response.^6^ TCR T cell therapies are genetically modified to target specific, internally derived peptides presented on tumor cell surfaces by human leukocyte antigen (HLA) molecules. For a particular TCR T cell therapy to function, a person must express the appropriate HLA type complexed with the tumor peptide that the TCR was engineered to target. Screening is therefore required to identify individuals most likely to benefit from any given product in this therapeutic modality.

To maximize the number of individuals eligible for TCR T cell therapy, the targeted peptide-HLA complex must be carefully selected. High genetic variability exists in the alleles encoding distinct HLA molecules; however, structural and functional homologies within alleles from the same allele group may allow presentation of the same antigenic peptide by multiple different alleles.^7^ HLA molecules have structural requirements for the peptides they are capable of presenting. Therefore, peptides derived from any given cancer-associated protein are typically only able to bind with sufficient affinity to a limited number of HLA alleles. Engineered TCRs are often designed to recognize tumor peptides complexed with HLA-A∗02 alleles because they are observed across many populations,^8^ thereby increasing the likelihood of patient eligibility. While A∗02:01 is the most common HLA-A∗02 subtype in most populations,^9^^,^^10^ other HLA-A∗02 subtypes represent significant proportions in some populations. Optimal characteristics of antigenic peptides for TCR T cell therapy include immunogenicity, cancer specificity, and expression across tumor types.^11^ Melanoma-associated antigen A4 (MAGE-A4) is a cancer testis antigen absent in most healthy tissues but differentially expressed in several solid tumors, including synovial sarcoma (SyS), lung, bladder, head and neck, ovarian, and esophageal cancers.^12^^,^^13^^,^^14^

Afamitresgene autoleucel (afami-cel, formerly ADP-A2M4) and its next-generation counterpart, uzatresgene autoleucel (uza-cel, formerly ADP-A2M4CD8), are TCR T cell therapies engineered to target MAGE-A4 in HLA-A∗02-eligible patients. Afami-cel and uza-cel express the same high-affinity MAGE-A4-targeted TCR, whereas uza-cel includes expression of an additional CD8α coreceptor for enhanced CD4+ T cell functionality and increased cytotoxic potency overall. Both have shown responses across multiple different cancer types.^15^^,^^16^

Here, we describe the preclinical characterization of the HLA-A∗02 alleles that are functionally able to bind the target MAGE-A4-derived peptide and activate the TCR, and are therefore defined as inclusion alleles for patient eligibility. Accurate and robust assays for HLA typing (high resolution) and MAGE-A4 expression are needed to screen and enroll patients into clinical trials of TCR T cell therapies including afami-cel and uza-cel. We present the validation of the HLA and MAGE-A4 assays suitable for identification of eligible patients, as well as data from a multinational screening study (NCT02636855) that prospectively evaluated HLA subtypes and MAGE-A4 profiles to determine eligibility to enroll in clinical trials assessing the safety and efficacy of TCR T cell therapy in patients with metastatic solid cancers and from the SPEARHEAD-1 registrational study (NCT04044768) of afami-cel in SyS and myxoid/round cell liposarcoma (MRCLS).

## Results

### Afami-cel selectivity to different HLA subtypes

Afami-cel displayed comparable *in vitro* potency toward peptide presented by HLA-A∗02:01, 02:02, 02:03, and 02:06, while the response to peptide in the context of A∗02:07 was >10-fold less potent than A∗02:01 (Table 1). Similar interferon-γ (IFN-γ) responses were observed from afami-cel toward MAGE-A4–positive (MAGE-A4+) tumor lines natively expressing A∗02:01, 02:02, 02:03, and 02:06, but no response was observed toward the same lines when expressing A∗02:07 (Figure S1) in the absence of added exogenous peptide. Based on the above functional study, HLA-A∗02:01, A∗02:02, A∗02:03, and A∗02:06 were defined as inclusion alleles. Although similar or greater responses were observed toward target lines expressing HLA-A∗02:05 compared with HLA-A∗02:01, previous study has identified alloreactivity toward this allele.^17^ Therefore, HLA-A∗02:05 was defined as an exclusion allele. Alleles sharing the same protein sequence in domains α1 and α2 (P group) are functionally identical and are also considered inclusion or exclusion alleles. For HLA eligibility in clinical trials investigating afami-cel and uza-cel, a patient should have at least one inclusion HLA-A∗02 allele and no exclusion allele (A∗02:05P). The ability of the HLA typing assay selected to perform the necessary high-resolution (two-field) typing to differentiate these relevant alleles was demonstrated in an accuracy study.

**Table 1:** Afami-cel reactivity to MAGE-A4 peptides presented by different HLA-A2 subtypes, as determined *in vitro* using an IFN-γ cell-ELISA method following challenge of afami-cel with exogenous MAGE-A4 peptide in the context of MAGE-A4-negative tumor lines transduced with HLA-A2 subtypes

Transduced HLA-A∗02 allele	Average log(EC_50_) M
HLA-A∗02:01	−7.8
HLA-A∗02:02	−8.1
HLA-A∗02:03	−7.4
HLA-A∗02:05	−8.8
HLA-A∗02:06	−8.3
HLA-A∗02:07	−6.5

EC_50_, half-maximal effective concentration; ELISA, enzyme-linked immunoassay; HLA, human leukocyte antigen; MAGE-A4, melanoma-associated antigen A4.

### Accuracy of the SeCore assay for HLA typing

When using the SeCore assay, all 70 samples yielded typing results with the SeCore assay that were consistent with the reference genotype, either from the published database or established by the AllType next-generation sequencing (NGS) assay. Among these 70 samples, 27 yielded results without ambiguities (i.e., only one possible genotype), whereas 43 gave ambiguous results (i.e., two or more possible genotypes) requiring group-specific sequencing primers (GSSP) sequencing. GSSP sequencing completely resolved 40 of these ambiguities (i.e., only one possible genotype remained). Two of the three remaining samples with ambiguities included null alleles resulting from insertion or deletion in the coding sequence, and the uTYPE software required, by design, the analyst to confirm the sequence to resolve the ambiguity. For the last sample, GSSP sequencing reduced the list of ambiguities to only two possible genotypes, including the reference genotype. The alternative allele combination (A∗02:135/69:02) could not be excluded by GSSP but was flagged by the uTYPE software as a combination of two rare alleles. With all samples being concordant, the lower limit of the one-sided 95% CI of concordance between the reference and the SeCore genotypes exceeded 95% (the threshold established in the FDA guidance for industry, 2015) with both the Clopper-Pearson exact test (95.81%) and the Wilson exact test (96.28%).

### HLA typing in the screening study and SPEARHEAD-1

**Figure 1:**
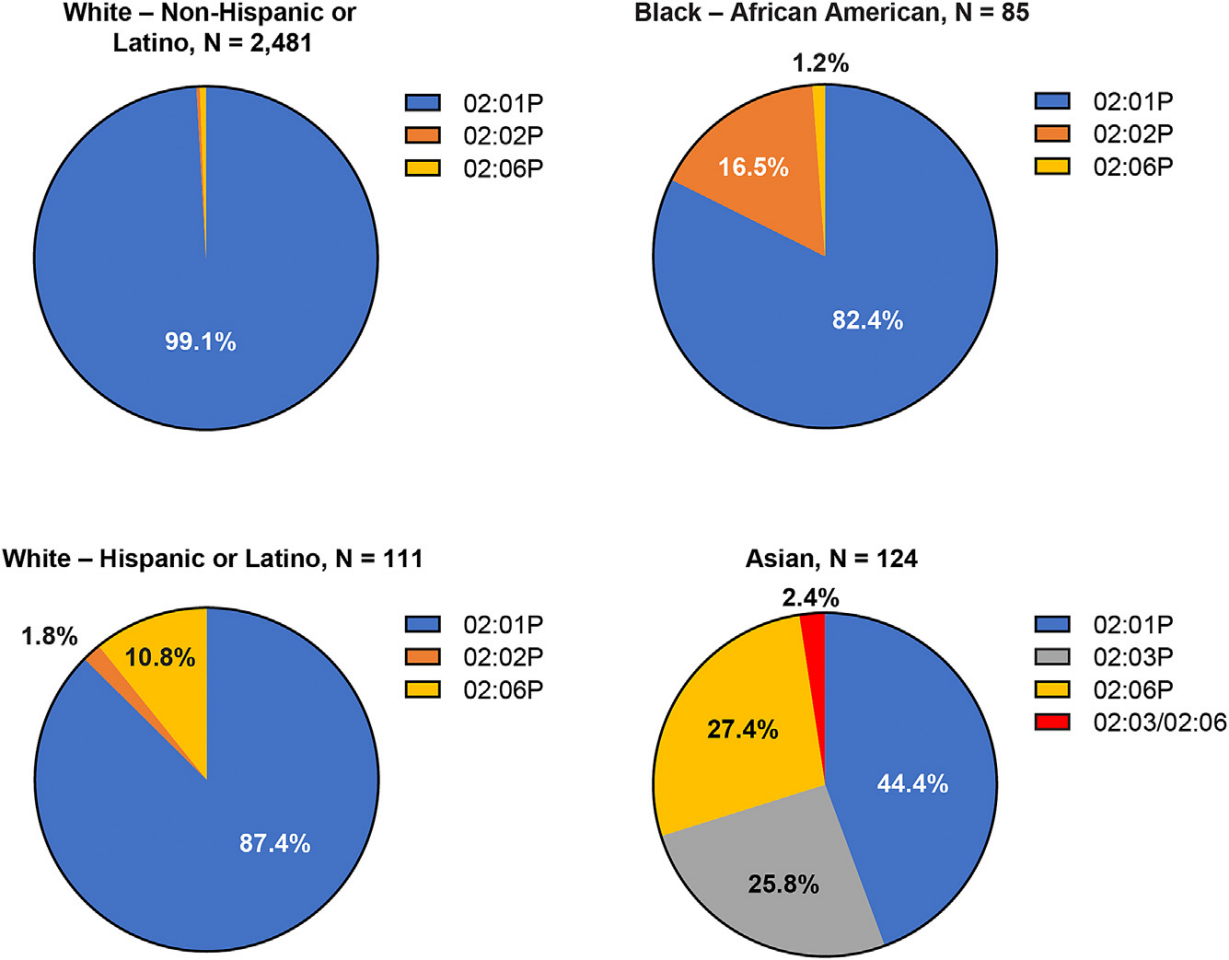
Relative contribution of HLA-A∗02 inclusion alleles to HLA eligibility for afamitresgene autoleucel by race and ethnicity Overall, most HLA-eligible participants were eligible based on the expression of A∗02:01 or one of its P-group members (in blue, e.g., 02:09 or 02:642), either as the only inclusion allele (heterozygous or homozygous), or combined with another inclusion allele. However, some participants were eligible based only on the expression of other inclusion alleles: A∗02:02 (orange), 02:03 (gray), 02:06 (yellow), or both 02:03 and 02:06 (red). The percentage of eligible participants exclusively expressing these inclusion alleles varied greatly by race and ethnicity. HLA, human leukocyte antigen.

A total of 6,606 patients from 43 sites in the US (30), Canada (1), Spain (7), the UK (2), and France (3) had their HLA-A type determined (6,167 from the screening study and 439 from the SPEARHEAD-1 study); among them, 2,959 (44.8%) were eligible based on the criteria for receiving afami-cel or uza-cel. Patients who had both an inclusion allele and an A∗02:05P allele were ineligible (*n* = 29; 0.44% of those screened). In patients for whom demographic information was available, eligibility rate was different between races and ethnicitiesr (Table 2). While a higher percentage of White patients was eligible due to HLA-A∗02:01P, adding A∗02:02, 02:03, and 02:06 as inclusion alleles significantly increased HLA eligibility in some other populations, in particular A∗02:06 in Hispanic and Latino patients, A∗02:02 in African American patients, and both A∗02:03 and A∗02:06 in Asian patients (Figure 1). Among eligible participants, the percentages of patients eligible due to the expression of at least one of these three alleles (A∗02:02, 02:03, and 02:06), without also expressing A∗02:01P, were: 12.6% of Hispanic or Latino, 17.7% of Black or African American, and 55.6% of Asian patients.

**Table 2:** HLA-A typing results by race and ethnicity

	Overall	
Race/ethnicity	Screened, *N* (%)	Eligible, *n* (%)
White, not Hispanic or Latino	5,249 (79.5)	2,481 (47.3)
White, Hispanic or Latino	260 (3.9)	112 (43.1)
White, not specified	58 (0.9)	31 (53.4)
Black or African American	319 (4.8)	85 (26.6)
Asian	435 (6.6)	124 (28.5)
American Indian or Alaska Native	22 (0.3)	8 (36.4)
Native Hawaiian or Pacific Islander	14 (0.2)	1 (7.1)
Not recorded	30 (0.5)	15 (50.0)
Other	219 (3.3)	102 (46.6)
Total	6,606 (100.00)	2,959 (44.8)

HLA, human leukocyte antigen.

### Performance of the MAGE-A4 IHC clinical trial assay

The anti-MAGE-A4 antibody (clone OTI1F9) showed specific staining of MAGE-A4 without cross-reactivity to MAGE-A1, -A2, -A3, -A6, -A9 (in MAGE-A-transduced NALM6 cell lines), -A10 (in the Mel526 cell line with endogenous high MAGE-A10 expression), and -A11 and -A12 (in Mel624). Additional details associated with the MAGE-A4 immunohistochemical (IHC) clinical trial assay are in the supplemental information, Tables S1–S4, and Figures S2–S16. The anti-MAGE-A4 antibody (clone OTI1F9) showed rare staining (0.28%) to MAGE-A8-transduced NALM6 cell line (high MAGE-A8 expression in 56% of cells), and minor cross-reactivity to MAGE-A10-transduced NALM6 cell line, artificial systems with extremely high MAGE-A8 or MAGE-A10 expression. The rare staining by anti-MAGE-A4 (clone OTI1F9) of MAGE-A8 had negligible impact on the diagnostic accuracy of the MAGE-A4 IHC clinical trial assay. The minor cross-reactivity of anti-MAGE-A4 antibody (clone OTI1F9) did not change the MAGE-A4 diagnosis status in tumor tissues with high MAGE-A10 expression as determined by a MAGE-A10 IHC clinical trial assay used to screen patients for a MAGE-A10-targeting TCR T cell study (Figure S6E). In the analytical validation, the tumor/tissue samples showed different MAGE-A4 prevalence in a broad range of solid tumors but not in normal tissues (except testis and placenta). The precision of the MAGE-A4 IHC clinical trial assay was validated at the cutoff (≥30% at ≥2+ intensity) with ≥80% inter-run/intra-run concordance (mostly ≥90%). Inter-lab assay transfer showed 100% concordance on a series of samples of multiple indications. Pathologists’ scoring showed ≥80% (mostly ≥90%) intra-/inter-reader concordance for a series of samples of multiple indications.

### MAGE-A4 expression

**Figure 2:**
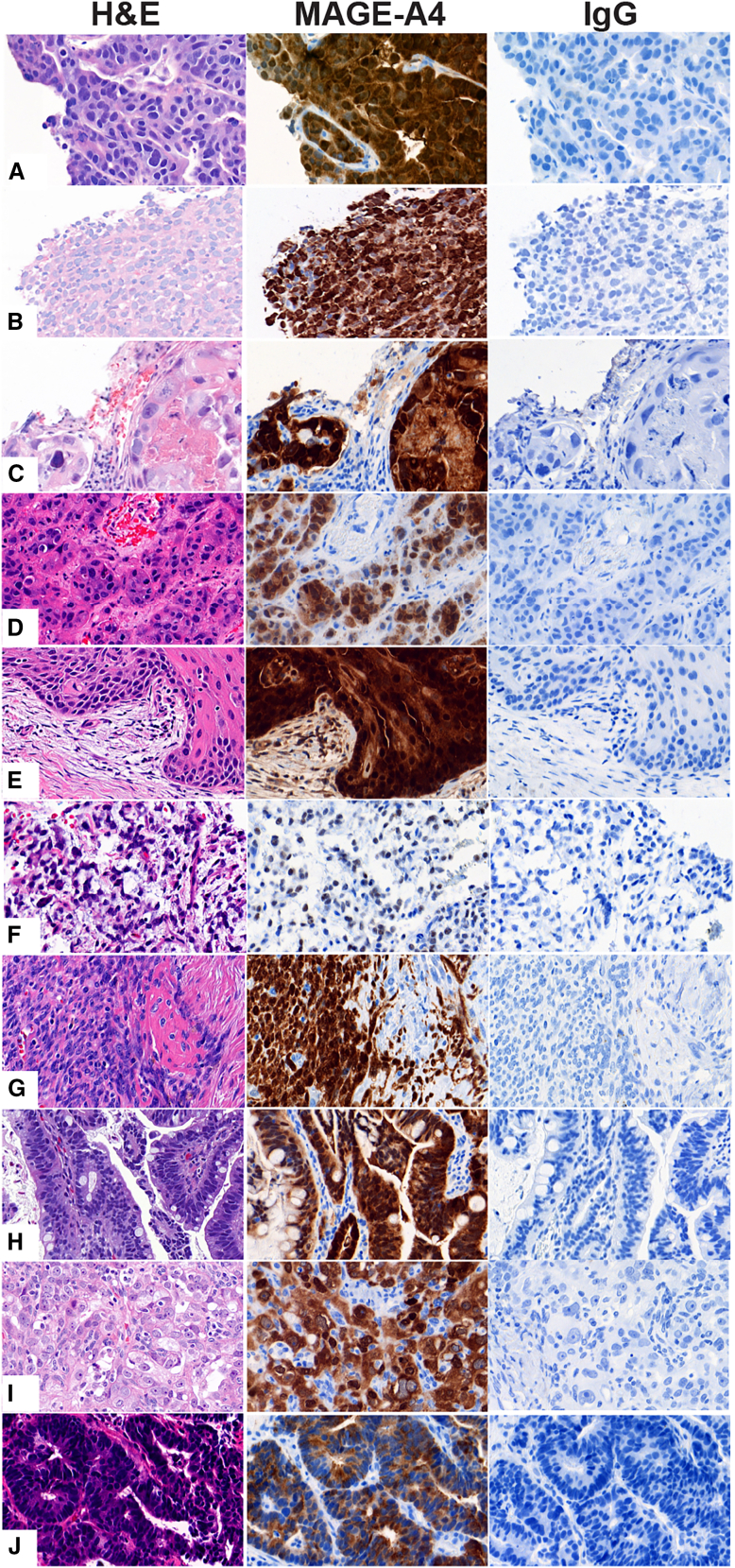
Representative images of histological and MAGE-A4 staining in different cancers screened Tissues from patients with esophagogastric junction (A), melanoma (B), non-small cell lung sarcoma (C), urothelial (D), head and neck (E), myxoid/round cell liposarcoma (F), synovial sarcoma (G), esophageal (H), ovarian (I), or gastric (J) cancers. The cancer tissue was stained with H&E, anti-MAGE-A4 antibody (MAGE-A4), and isotype control (IgG), visualized in the left, middle, and right columns, respectively. MAGE-A4 immunoreactivity demonstrated specific expression in each cancer tissue. Magnification of the images: 40×. H&E, hematoxylin and eosin; IgG, immunoglobulin G; MAGE-A4, melanoma-associated antigen A4.

In the 2,959 HLA-eligible patients, 1,750 had tumor samples evaluable for MAGE-A4 across 31 sites in the US (18), Canada (1), France (3), the UK (2), and Spain (7); among these, 447 patients were MAGE-A4+ (≥30% tumor cells stained at ≥2+ intensities). Representative IHC images, with specific staining of MAGE-A4 in the cytoplasm and nuclei of tumors cells with different staining intensities (0–3+), are shown in Figure 2, and expression in the different tumor types in Figure S17. Most tumor samples (93%) were collected within 4 years of testing, with 55% of them collected within 1 year of testing, and overall archival time ranging from 0 to 20 years. Overall, MAGE-A4+ rate varied among individual tumor types (Figure S18A) but remained similar within 5 years of tissue archival (Figure S18B). Lower MAGE-A4 prevalence observed in samples collected >5 years before testing in some tumor types may reflect the small sample size of tissue archived >5 years before testing, differences in tumor biology, or compromised MAGE-A4 stability (Figures S18A and S18B). However, no relationship between MAGE-A4 protein score (P score) and tissue archival time could be demonstrated; some samples archived for up to 9 years still had a P score of 97, indicating stability of MAGE-A4 in formalin-fixed, paraffin-embedded (FFPE) archived tissues and suitability of older tissue blocks for eligibility purposes (Figure S18C).

**Figure 3:**
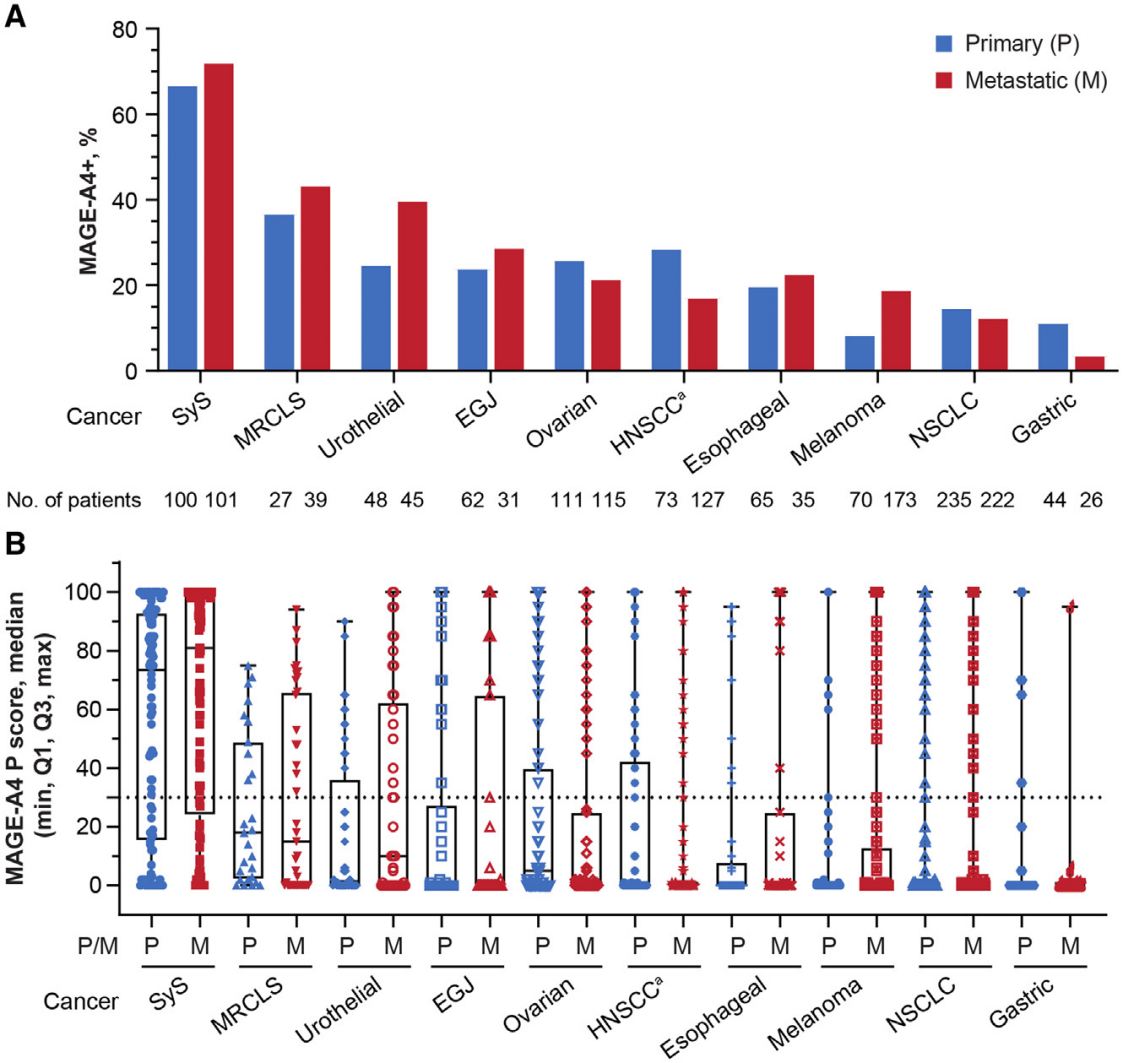
MAGE-A4 positivity and expression level by tissue location (primary or metastatic) across cancer types (A) MAGE-A4 positivity. (B) MAGE-A4 expression level. ^a^*n* = 199 HNSCC, *n* = 1 “other” head and neck cancer histology. The dotted line represents the cutoff value of the P score indicating MAGE-A4 positivity. EGJ, esophagogastric junction cancer; HNSCC, head and neck squamous cell carcinoma; M, metastatic; MAGE-A4, melanoma-associated antigen A4; MRCLS, myxoid/round cell liposarcoma; NSCLC, non-small cell lung cancer; P, primary; P score, protein score; SyS, synovial sarcoma.

**Figure 4:**
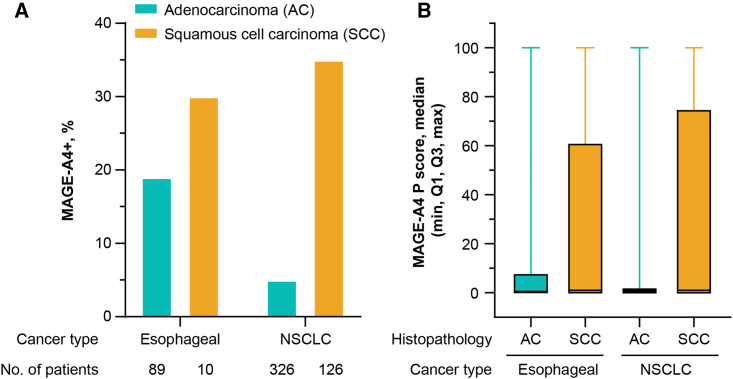
MAGE-A4 positivity and expression level by histopathology (A) MAGE-A4 positivity. (B) MAGE-A4 expression level. AC, adenocarcinoma; MAGE-A4, melanoma-associated antigen A4; MAGE-A4+, MAGE-A4 positive; NSCLC, non-small cell lung cancer; P score, protein score; SCC, squamous cell carcinoma.

**Figure 5:**
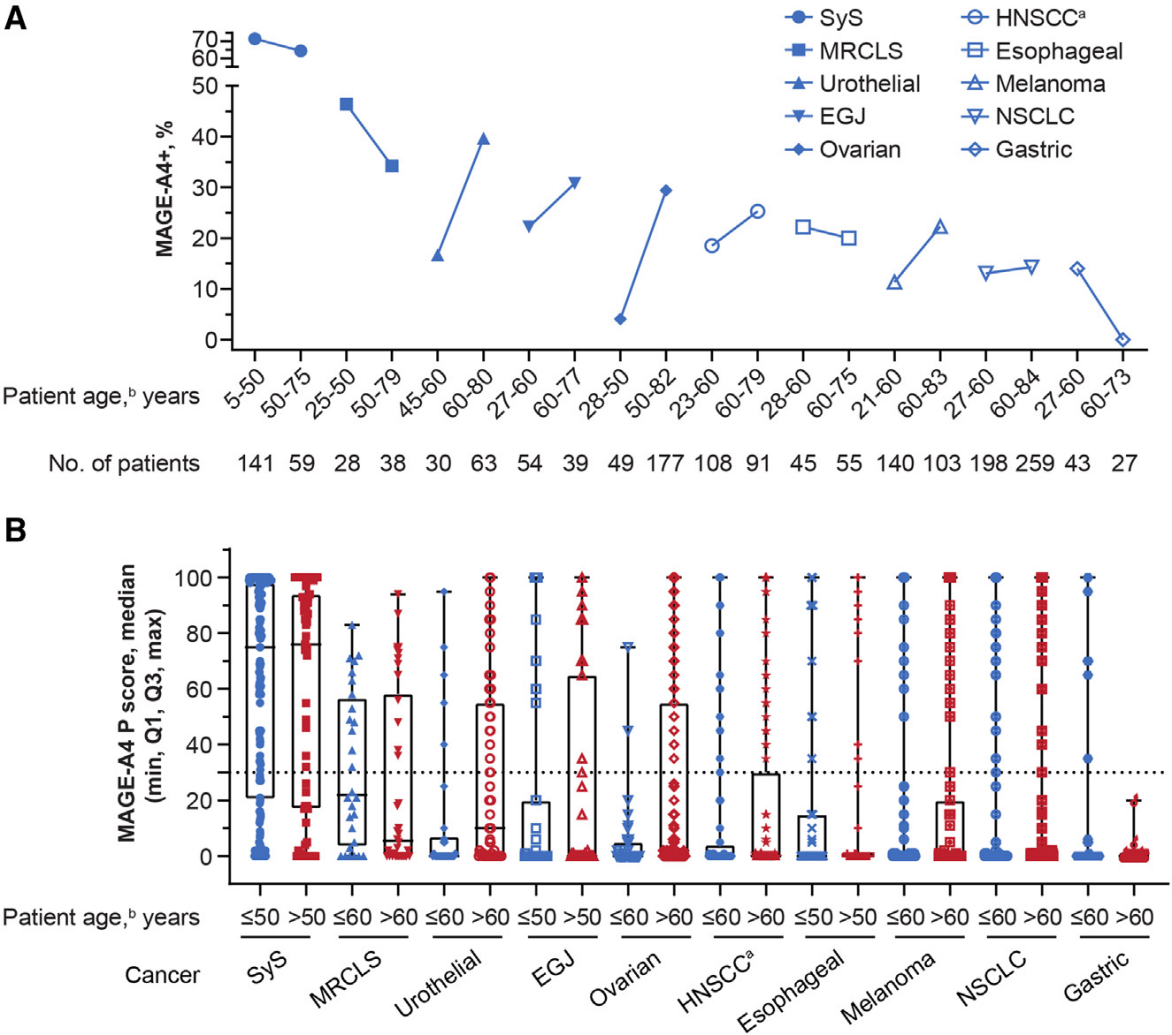
Effect of patient age on MAGE-A4 positivity and expression level across cancer types (A) MAGE-A4 positivity. (B) MAGE-A4 expression level. ^a^*n* = 199 HNSCC, *n* = 1 “other” head and neck cancer histology. ^b^Patient age was determined at biopsy collection. The dotted line represents the cutoff value of the P score indicating MAGE-A4 positivity. EGJ, esophagogastric junction cancer; HNSCC, head and neck squamous cell carcinoma; MAGE-A4, melanoma-associated antigen A4; MAGE-A4+, MAGE-A4 positive; MRCLS, myxoid/round cell liposarcoma; NSCLC, non-small cell lung cancer; P score, protein score; SyS, synovial sarcoma.

The MAGE-A4+ rate was highest in SyS (70%, 140/201) and lowest in gastric cancer (9%, 6/70), but was seen across all tumor types investigated, including MRCLS (40%, 27/67), urothelial cancer (32%, 30/93), esophagogastric junction (EGJ) cancer (26%, 24/93), ovarian cancer (24%, 54/226), head and neck cancer (22%, 43/200), esophageal cancer (21%, 21/100), melanoma (16%, 39/243), and NSCLC (14%, 63/457) (Figure S17). MAGE-A4 expression level was highest, on average, in SyS (median P score = 76), followed by MRCLS (median P score = 15), urothelial cancer (median P score = 5), ovarian cancer (median P score = 2), and the rest of the cancer types (median P score = 0) (Figure S17B), with some samples reaching a P score of 100 in all indications other than MRCLS (highest P score = 94). In a non-pairwise analysis of all tested samples, MAGE-A4 was detected at relatively similar frequencies in primary and metastatic tumors, although higher MAGE-A4+ rate was observed in metastatic tumors of urothelial cancer and melanoma while lower MAGE-A4+ rate was observed in metastatic tumors of head and neck cancer and gastric cancer (Figure 3). In pairwise analysis of MAGE-A4 expression in both primary and metastatic tumor tissues, 16 patients had both samples and 81% (13/16) of these patients had the same MAGE-A4 diagnosis status (positive or negative) regardless of tissue location (primary or metastatic) (data not shown). In both esophageal cancer and NSCLC, MAGE-A4+ rate and expression level were higher in squamous cell carcinoma (SCC) samples, compared with adenocarcinoma (AC) samples (Figure 4). MAGE-A4+ rate was not correlated with the age of patients in esophageal cancer, EGJ cancer, head and neck cancer, NSCLC, or SyS. An apparent negative correlation of MAGE-A4 positivity with patient age was observed in gastric cancer and MRCLS, while an apparent positive correlation was observed in melanoma, ovarian cancer, and urothelial cancer (Figure 5).

As per the univariate and multivariate analyses within each cancer type, the covariates were generally not associated with MAGE-A4 expression, except for patient age in ovarian cancer and histology in NSCLC. After adjusting for confounding factors, patient age (odds ratio [OR] = 9.86; 95% CI, 2.87–62.12) was positively associated with the MAGE-A4 expression in ovarian cancer. In addition, MAGE-A4 expression in NSCLC was significantly higher (OR = 10.02; 94% CI, 5.36–19.54) in samples of SCC compared with AC.

## Discussion

Identifying individuals who are most likely to benefit from treatment is a requirement for precision medicine therapeutic products. Based on the mechanism of action of TCR T cell therapy, screening for HLA genotype and tumor antigen expression are the two components of the biomarker-driven identification of eligible patients. Based on the HLA and MAGE-A4 prevalence for clinical trial enrollment as reported in this study, eligibility (HLA eligible/MAGE-A4+) may vary from 4% to 31% depending on indications, highlighting the need for, and importance of, implementing a biomarker screening program that is both reliable and easily accessible for a TCR T cell therapy. Results from the accuracy evaluations of the HLA typing assay and MAGE-A4 IHC clinical trial assay used in the screening study reported here indicate that they are reliable when assessing eligibility for clinical trials investigating afami-cel and uza-cel.

The process of designing a targeted TCR is founded in the identification of a tumor antigen that binds with sufficient affinity to particular HLA molecules. Because alleles in the HLA-A∗02 group are most frequently expressed in many populations around the world, they are often preferred when identifying appropriate tumor antigens.

Eligibility rates based on HLA criteria in the screening protocol and the SPEARHEAD-1 study were consistent with expectations based on public databases on HLA-A∗02 allele frequencies. Data from the US National Marrow Donor Program^18^^,^^19^ show that A∗02:01P is generally the most frequent allele, but its frequency is higher in White populations (47.5% of individuals) and lower in Asian (18%) and Black or African American (23%) populations (Table S5). In our study, inclusion of A∗02:02P, A∗02:03P, and A∗02:06P increased the proportion of eligible patients across Asian, Hispanic or Latino, and Black or African American populations. A higher percentage of White patients than other races and ethnicities are eligible to receive an A∗02:01-restricted immunotherapy such as afami-cel. In this report, we show that the validations of alleles other than A∗02:01 (A∗02:02, A∗02:03, and A∗02:06) as inclusion criteria for studies offset genetic bias to some extent and increase eligibility in other populations. Whereas public databases report allele frequencies in specific populations, they do not indicate how specific alleles co-segregate within subgroups of a given race or ethnicity, which can lead to over- or underestimation of the percentage of population positive for a set of alleles from the same locus. Our genotyping data provide that insight.

Our results indicate that MAGE-A4 expression can be reliably assessed in fresh biopsy or archival tissues (up to 5 years old); however, the effect of storage time of FFPE blocks on MAGE-A4 positivity beyond 5 years would need further investigation in a longitudinal study. Consistent with previous reports (Table S6 and references thereof),^20^ MAGE-A4 expression in this study was found at varying levels across tumor types. The difference of MAGE-A4 prevalence in this study in comparison with literature reports may be due to differences in assays used and their positivity cutoff, as well as patient populations and disease clinicopathology. The clinical utility of HLA/MAGE-A4 as biomarkers in selecting patients for MAGE-A4-targeted TCR T cell therapies has been demonstrated in two phase 1 trials in multiple indications, including SyS, ovarian cancer, urothelial cancer, and head and neck SCC.^15^^,^^16^ Their clinical utility has been further confirmed in a phase 2 trial of afami-cel in SyS and MRCLS.^21^ Thus, the MAGE-A4 prevalence reported here is of significance in guiding future clinical trial development for anti-MAGE-A4 TCR T cell therapies. In our samples, SyS showed the highest MAGE-A4 expression, whereas gastric cancer showed the lowest. Among the potential factors affecting MAGE-A4 expression, we found no consistent correlation between patient age and MAGE-A4 expression; however, older age of patients was associated with higher MAGE-A4 expression in ovarian cancer, consistent with previous reports.^22^ In addition, MAGE-A4 expression in relation to tumor location (primary vs. metastatic lesions) was generally comparable across the cancer types included in this study, although higher MAGE-A4 expression in metastatic melanoma lesions was noted, in line with what was shown previously.^23^ This implies that a cancer tissue, regardless of its origin/tissue location, may be used to determine a patient’s MAGE-A4 eligibility at screening, without significant impact on the screening efficiency. Finally, we found that SCC compared with AC had significantly higher MAGE-A4 expression in NSCLC, consistent with prior reports.^24^ As patient age, tumor location (primary vs. metastatic), and histology type impact prognosis after cancer treatment, increased understanding of their relationships to MAGE-A4 expression may shed light on clinical development of MAGE-A4-targeted T cell therapy, including patient screening/selection and trial design.

There are some limitations of this study. First, MAGE-A4 prevalence and expression levels may be subjected to tumor heterogeneity, clinicopathology, and sample size; most of the patients in this screening study only had one tissue block submitted for MAGE-A4 testing and some tumor types had a limited samples size (e.g., MRCLS, *n* = 67; gastric cancer, *n* = 70). Second, factors that may affect the determination of MAGE-A4 expression, including tumor location (primary vs. metastatic) and archived tissue storage time, are neither pairwise (for tumor locations) nor longitudinal (for archived tissue storage time), and the impact of archived tissue storage time greater than 5 years (7% of the total samples tested) on MAGE-A4 expression may be uncertain. Third, the actual target of afami-cel and uza-cel is the complex of HLA-A∗02 and the MAGE-A4-derived peptide; however, there is currently no technology able to detect that complex on the surface of tumor cells, especially in FFPE samples. The current screening tests (germline HLA typing and expression of MAGE-A4 by IHC in tumor samples) are admittedly a surrogate for the detection of the peptide-HLA complex. However, the overall response rate per RECIST 1.1 obtained in SyS and other indications demonstrates the clinical validity of the screening process.^15^^,^^16^^,^^21^

Taken together, HLA-A genotype and MAGE-A4 tumor expression are key biomarkers to assess patient eligibility to enroll in various trials of TCR T cell therapy, including those investigating the safety and efficacy of afami-cel and uza-cel. This is the first report of a large-scale HLA and MAGE-A4 prospective screening study with demonstrated clinical utility of the biomarkers, setting a foundation of biomarker screening for TCR T cell therapies, and illustrating the extent of screening required for therapies of this type. The findings on HLA prevalence in different races/ethnicities and MAGE-A4 expression in different tumor and histology types and the impact of patient age in certain cancers (e.g., ovarian cancer) may guide future clinical development of TCR T cell therapies, including disease selection strategy, patient eligibility criteria, trial design, and investigation into different HLA-restricted TCRs. The impact of tumor location (primary vs. metastatic) and archived tissue storage time on antigen determination also provides practical guidance on sample collection for screening and eligibility determination. Future efforts are warranted to further address assay implementation issues and to develop accurate and robust single-plex or multiplex screening assays that are easily deployable and accessible to meet the functional needs of TCR T cell therapies.

## Materials and methods

### Functional assessment of afami-cel selectivity to different HLA subtypes

To explore the functional response of MAGE-A4-targeted TCR T cell therapies to common HLA-A∗02 subtypes, MAGE-A4+ HLA-A∗02-negative tumor cell lines were transduced using lentiviral vectors expressing HLA-A∗02 alleles, green fluorescent protein, and puromycin-N-acetyltransferase. These lines were selected through culture in puromycin, and comparable levels of transgene expression were confirmed by flow cytometry (data not shown). The ability of these lines to induce an afami-cel response was subsequently assessed by IFN-γ cell enzyme-linked immunoassay (ELISA). Generation of afami-cel TCR T cells was described previously.^17^ For the cell-based ELISA, 384-well plates were coated with IFN-γ capture antibodies overnight followed by plating of target cells (10^4^/well), effector T cells (10^4^/well) and/or peptide, or IFN-γ standards. After 48 h, the plates were washed and the assay was carried out following the manufacturer’s protocol (Human IFN-gamma DuoSet ELISA, R&D Systems, Minneapolis, MN), with the use of a luminescent HRP substrate (Glo Substrate, R&D Systems). Luminescence was measured using a FLUOstar Omega plate reader (BMGLabtech, Cary, NC). Peptide response curve fitting was performed using the drc R package using a three-parameter log-logistic function.^25^

### Protocol design

The screening and SPEARHEAD-1 studies adhered to the principles outlined in the Declaration of Helsinki and were conducted according to the International Council for Harmonisation’s Guideline for Good Clinical Practice. Written informed consent was obtained from all patients prior to any study-related procedures being performed. Design of SPEARHEAD-1 has been reported previously.^21^

Men and women aged ≥18 to ≤75 years with advanced solid or hematologic malignancy and a life expectancy >3 months could enroll in the screening study. Eligible cancer types included melanoma, NSCLC, head and neck, gastric, EGJ, esophageal, ovarian, urothelial, SyS, and MRCLS cancers. Patients must have been able to provide blood samples and tumor samples (e.g., archived FFPE tumor blocks or tissue sections, or fresh biopsies if feasible).

Patients’ sex, race, and ethnicity were determined based on self-reporting by checking boxes associated with their demographics. For biological sex, there were two options (i.e., male or female). The options for race and ethnicity were as follows: White, Black or African American, Asian, American Indian or Alaska Native, Native Hawaiian or Pacific Islander, Hispanic or Latino, not Hispanic or Latino, or other.

The primary endpoints of the screening protocol were determination of MAGE-A4 antigen expression profile and HLA genotype for subsequent assessment of their eligibility for clinical trials of afami-cel and uza-cel TCR T cell therapies. The exploratory endpoint was determination of incidence of antigen expression in different cancer types.

### HLA typing

Blood samples were collected from screened patients as the source of DNA for HLA-A typing. High-resolution (two-field) typing of HLA-A was required to discriminate inclusion, exclusion, and neutral A∗02 alleles and determine eligibility. All samples were typed via the SeCore assay (One Lambda, Thermo Fisher Scientific, Los Angeles, CA), a Sanger sequencing-based typing assay that has received 510(k) approval from the FDA. In brief, amplification by polymerase chain reaction of HLA-A alleles using locus-specific primers was followed by bi-directional sequencing of exons 1 to 5 on an ABI 3730xl DNA Analyzer (Thermo Fisher Scientific) and subsequent analysis using uTYPE HLA Sequence Analysis Software (Thermo Fisher Scientific). When necessary, ambiguities were resolved using the SeCore GSSP or an SSP assay if an appropriate GSSP was not readily available. Buccal swabs were used for HLA typing of patients who consented remotely (*n* = 58); those who were determined to be HLA eligible and whose tumor expressed MAGE-A4 had their HLA type confirmed with a blood sample (*n* = 4). All testing occurred at Histocompatibility Laboratory Services, American Red Cross, in Philadelphia, and IMGM, in Martinsried, Germany.

An accuracy study using both well-characterized samples expressing several frequent and less frequent A∗02 alleles and DNA samples from patients with SyS was conducted to assess the capacity of the SeCore assay to correctly assign genotype and its suitability for the intended use. The AllType NGS assay (One Lambda) was used as the predicate for samples that were not well characterized or for which the published genotype was erroneous. Seventy DNA samples were evaluated including 64 from Epstein-Barr virus-transformed lymphoblastic cell lines, which were procured from the International Histocompatibility Working Group (*n* = 34) or from the Class I UCLA DNA Reference Panel (*n* = 28), or were derived in-house (*n* = 2). In addition, six samples were from patients with SyS who were screened for eligibility to participate in a TCR T cell therapy clinical trial. Concordance between the SeCore genotype and the reference genotype (published or established with the AllType assay) was defined as the reference genotype being either identical to the SeCore genotype (absence of ambiguities) or being included among the possible SeCore genotypes (presence of ambiguities).

### MAGE-A4 expression

MAGE-A4 testing of tumor samples, either an archived FFPE specimen or a fresh biopsy, in HLA-eligible patients was carried out via an IHC clinical trial assay. Tissue blocks were cut as 4-μm thickness slides, pretreated in the 3 in 1 PT module with TRS low pH antigen retrieval solution (Dako-K8005) at 60°C for 2 h and then at 97°C for 20 min, and then stained on the Dako autostainer Link 48 platform with an anti-MAGE-A4 monoclonal antibody (clone OTIF9, Origene, TA505362, 10 μg/mL) and an IgG isotype control antibody (IgG2a, Sigma Aldrich, M9144, 10 μg/mL) for 30 min at room temperature (22°C–25°C), visualized by the EnVision+ System-HRP Labelled Polymer (Dako-K4001) combined with a Dako Liquid DAB Substrate Chromogen System (Dako-K3468) for 30 and 5 min, respectively, counterstained with hematoxylin for 5 min, and then finally cover-slipped on the Sakura coverslipper, which are all qualified and validated at CellCarta. The MAGE-A4 IHC clinical trial assay was performed at a Clinical Laboratory Improvement Amendments 1998-certified and College of American Pathologists-accredited central laboratory, and specificity, precision, inter-lab concordance, and pathologist scoring concordance were analyzed to evaluate assay performance (details in supplemental information).

MAGE-A4 expression was determined by both percentage of tumor cell staining and intensity of cell staining (nuclear/cytoplasmic staining at 0, 1+, 2+, 3+ intensity). MAGE-A4 expression level was defined by P score (percent of tumor cells staining at 2+, 3+). P score was initially defined as tumor samples with percent cell staining at ≥1+ with 10% cutoff for screening/enrollment. A protocol amendment shifted this cutoff to a P score ≥30% at ≥2+ for MAGE-A4 positivity, which is used in all clinical trials. Both the MAGE-A4+ rate (%) and MAGE-A4 expression level (median P score) are reported here, based on the cutoff of P score ≥30% at ≥2+. H score is assessed as part of our translational research but is not used to determine eligibility, therefore it is not included with the screening protocol data.

### Statistical analyses

The biomarker (HLA and MAGE-A4) screening samples were collected between May 22, 2017 and November 19, 2021 for this analysis for the screening study (NCT02636855) and from June 26, 2019 until October 22, 2021 for SPEARHEAD-1 (NCT04044768). Covariates of MAGE-A4 expression used in this study were demographics (sex, age), histopathology (cancer type, tumor subtype [primary vs. metastatic], estimated number of cancer cells, and estimated percentage of inflammatory cells), histology type (AC and SCC), and FFPE sample storage time. Covariates were assessed by univariate and multivariate methods using logistic regression modeling, and results are presented as odds ratios with 95% CIs and *p* values. Age, estimated number of cancer cells, FFPE sample storage time, sex, tumor subtype, and histology were categorical variables, whereas estimated percentage of inflammatory cells was a continuous variable. Software tools used for this study are available as open-source R v.3.6.3 and associated packages.

## Data and code availability

All data relevant to the study are included in the article or uploaded as supplemental information. The raw datasets generated, used, and analyzed during the current study are available from the corresponding author on reasonable request.

## Supplemental information

Document S1. Figures S1–S18 and Tables S1–S6

Document S2. Article plus supplemental information
