# Supplementary material for: Identifying MAGE-A4-positive tumors for TCR T cell therapies in HLA-A∗02-eligible patients
Source: Mol Ther Methods Clin Dev. 2024 May 14;32(2):101265. doi: 10.1016/j.omtm.2024.101265 (PMC11170170; doi:10.1016/j.omtm.2024.101265)
Supplement: Document S1. Figures S1–S18 and Tables S1–S6 [file mmc1.pdf]

OMTM, Volume *32*

Supplemental information

Identifying MAGE-A4-positive tumors for TCR T cell therapies in HLA-A*02-eligible patients

Tianjiao Wang, Jean-Marc Navenot, Stavros Rafail, Cynthia Kurtis, Mark Carroll, Marian Van Kerckhoven, Sofie Van Rossom, Kelly Schats, Konstantinos Avraam, Robyn Broad, Karen Howe, Ashley Liddle, Amber Clayton, Ruoxi Wang, Laura Quinn, Joseph P. Sanderson, Cheryl McAlpine, Carly Carozza, Eric Pimpinella, Susan Hsu, Francine Brophy, Erica Elefant, Paige Bayer, Dennis Williams, Marcus O. Butler, Jeffrey M. Clarke, Justin F. Gainor, Ramaswamy Govindan, Victor Moreno, Melissa Johnson, Janet Tu, David S. Hong, and George R. Blumenschein Jr.

SUPPLEMENTAL TEXT

MAGE-A4 immunohistochemistry (IHC) clinical trial assay (CTA)

Samples

Tumor and normal tissue

Formalin-fixed paraffin-embedded (FFPE) tissue blocks were obtained in accordance with the Helsinki Declaration of 1975, following patient privacy procedures and approval by the hospital ethics committee (EC/PC/avl/2016.003) or purchased from different commercial providers (Proteogenex, QualTek Molecular Laboratories, Adaptimmune, ABS Bio, BioIVT, and Discovery Life Sciences).

Solid tumor indications (unique samples) used for MAGE-A4 validation (prevalence, precision, assay transfer, and inter-lot robustness): lung cancer (4), urinary bladder (2), HNSCC (33), ovarian cancer (44), gastric cancer (35), esophageal adenocarcinoma (31), esophageal squamous cell carcinoma (34), esophagastric junction adenocarcinoma (49), melanoma skin (2), synovial sarcoma (35), myxoid/round cell liposarcoma (35), endometrium carcinoma (37).

FFPE blocks used during the assay development and validation passed the following quality requirements: no tissue detachment, sufficient tissue, adequate staining (hematoxylin and eosin [H&E] or PTEN or ki67 IHC), and no impaired tissue integrity. Additionally, all tumor specimens were evaluated by a certified pathologist.

Cell lines

FFPE cell line slides (A375 and HCT116) with known expression levels of MAGE-A4 were procured, produced, characterized, and provided by Adaptimmune as run controls.

Additional cell lines were procured, produced, and characterized by Adaptimmune for specificity study of anti–MAGE-A4 monoclonal antibody (clone OTI1F9), including MAGE-As transduced NALM6 cell lines, NCI-H82, NCI-H466, Mel526, and Mel624, whose MAGE-As expression were shown by flow cytometry with Tomato Red reporter, quantitative reverse transcription polymerase chain reaction (RT-qPCR), or publicly available RNAseq database.

Tissue microarrays

TMA slides were purchased from US Biomax (MNO1021). All tissues were collected under the highest ethical standards, with the donor being informed completely and with their consent. US Biomax makes sure they follow standard medical care and protect the donors' privacy. Furthermore, all human tissues were collected under HIPAA-approved protocols and have been tested negative for HIV and hepatitis B and are approved for commercial product development.

Staining procedure

Before staining, slides were baked for 2 hours at 60°C and deparaffinized using the automated in pretreatment module: 3-in-1 specimen preparation procedure using TRS low pH antigen retrieval solution (K8005) (20 min, 97°C). The Envision detection system (EnVision+ System- HRP Labeled Polymer Anti-Mouse [Dako - K4001]) combined with a Dako Liquid DAB Substrate Chromogen System (Dako - K3468) was used for visualization. All stained slides were scanned as whole-slide images using a digital slide scanner (3DHISTECH, Budapest, Hungary).

Pathologist scoring

Slides were scored for the overall percentage of MAGE-A4–positive tumor cells and the intensity of MAGE-A4 staining. Only tumor cells were scored and any expression in surrounding stroma was ignored. All scoring was performed by a pathologist on either glass slides or high-resolution scanned whole-slide digital images.

Highly heterogenous tumors, such as synovial sarcoma and myxoid/round cell liposarcoma (MRCLS), require scoring in multiple high-power fields using the “field of view” method, while tumors with homogeneous MAGE-A4 expression are scored using regional method.

The percentage of MAGE-A4–positive tumor cells was determined at each intensity (0, 1+, 2+, and 3+ intensity) relative to the total number of viable tumor cells in the sample. When a MAGE-A4 signal was present in the cytoplasm and nucleus, the compartment with the highest-intensity expression was evaluated. Specific scoring rules were applied when scoring liposarcoma with myxoid regions and regions with complex arborizing vessel patterns. Arborizing vessels and the cell poor myxoid component were not taken into account when present. The high cellularity regions of the tumor were scored when scoring the myxoid liposarcoma cases.

A sample was considered MAGE-A4 positive if ≥30% tumor cells had a MAGE-A4 positivity at 2+ intensity or more (Figure S2).

Statistical analysis

A specific cutoff point (≥30% MAGE-A4–positive tumor cells stained at ≥2+ intensities) was applied on the scoring outcome to determine positivity/negativity for each sample. The positive/negative status was used to establish concordance. For precision assessment (intra- and inter-run variability), percent positive agreement and percent negative agreement of repeat staining of samples were based on the positive/negative status. The acceptance criterion for precision was set as 80% concordance at the slide level (nine replicates in three different runs for each of a minimum of four different samples per indication). Furthermore, the concordance on sample level is included for descriptive purposes.

Accuracy/specificity of the MAGE-A4 antibody

Several FFPE cell line slides and control tissue (normal testis) with known expression levels of MAGE-A4 were characterized to determine the MAGE-A4 specificity.

Cell line A375, which is known to be positive (datasheet Origene, clone OTI1F9; Sanderson et al., 2019) for MAGE-A4, demonstrated MAGE-A4–positive staining using the MAGE-A4 IHC CTA, while for cell line HCT116, known to be negative for MAGE-A4, no staining could be observed in all staining runs (Figure S3). In testis, nuclear and cytoplasmic MAGE-A4 staining was observed in the atrophic ducts and in the seminiferous tubules with strong intratubular staining, while no MAGE-A4 staining was demonstrated in the stroma (Figure S4).

No MAGE-A4 staining was observed in a normal human tissue TMA, except for human testis (Figure S5). The TMA with different (normal) human tissue types was evaluated for staining intensity and no positivity for MAGE-A4 could be observed in these normal tissues (breast, intestine, liver, lung, stomach, heart, fallopian tube) with a 10 µg/ml concentration of the primary antibody. For testis, nuclear and cytoplasmic MAGE-A4 positivity is present in the seminiferous tubules.

To further validate the specificity of anti–MAGE-A4 antibody, NALM6 parental cell line was transduced with different full-length MAGE-As (-A1, -A2, -A3, -A4, -A6, -A8, -A9, -A10). The expression of MAGE-As in NALM6-transduced cell lines was confirmed by flow cytometry with a reporter (Tomato Red) (Figure S6A). Anti–MAGE-A4 antibody stained specifically to MAGE-A4–transduced NALM6 cell line without cross-reactivity to MAGE-A1, -A2, -A3, -A6, and -A9. Rare staining (0.28%) by the anti–MAGE-A4 antibody was observed in MAGE-A8–transduced NALM6 cells (with a high MAGE-A8 expression level in >50% of cells), which is unlikely to change the diagnostic accuracy of an assay using the anti–MAGE-A4 antibody. Some low-level staining by the anti–MAGE-A4 antibody of MAGE-A10–transduced NALM6 cell line was also observed (Figure S6B).

To further investigate the cross-reactivity of anti–MAGE-A4 antibody to MAGE-A10, three cell lines (NCI-H82, NCI-H466, and Mel526) were chosen for further characterization. By qRT-PCR, NCI-H82 was shown to have high MAGE-A4 expression and high MAGE-A10 expression (MAGE-A4^{h}/MAGE-A10^{h}).

NCI-H466 was shown to have low MAGE-A4 expression and high MAGE-A10 expression (MAGE-A4^{l}/MAGE-A10^{h}). Mel526 was shown to have negligible MAGE-A4 expression and high MAGE-A10 expression (MAGE-A4^{n}/MAGE-A10^{h}) (Figure S6C). Anti–MAGE-A4 antibody showed strongly positive, weakly positive and negative staining in NCI-H82, NCI-H466, and Mel526, respectively, supporting the specificity of anti–MAGE-A4 antibody for MAGE-A4 without cross-reactivity to MAGE-A10 (Figure S6D). Some low-intensity staining could be observed with the anti–MAGE-A4 antibody in transduced NALM6 cells expressing extremely high levels of MAGE-A10 (Figure S6B), indicating a possible low-affinity cross-reactivity that can only be detected in this artificial system, which may not be physiologically or pathologically relevant.

Further evidence of the specificity of the MAGE-A4 CTA and estimation of the potential impact of the cross-reactivity with MAGE-A10 on its diagnostic value was provided by the analysis of data from 352 tumor samples from 10 different indications (melanoma, bladder cancer, NSCLC, head and neck cancer, esophageal cancer, esophagogastric junction cancer, ovarian cancer, gastric cancer, MRCLS, synovial sarcoma) stained for expression of both MAGE-A4 and MAGE-A10. These samples were screened under a screening protocol (ADP-0000-001, NCT02636855) used to determine eligibility for enrollment into one of two clinical trials using T cells directed against MAGE-A10 (ADP-0022-003, NCT02592577 and ADP-0022-004, NCT02989064) as well as a clinical trial using T cells directed against MAGE-A4 (ADP-0044-001, NCT03132922). For detection of MAGE-A10, a CTA based on a goat polyclonal antibody (Santa Cruz, Cat # sc-324906) was developed, validated, and used under a CLIA-certified laboratory to stain by IHC sections from FFPE tumor samples. Serial sections from the same samples were stained with the MAGE-A10 CTA. Similar to the MAGE-A4 CTA, scoring for the MAGE-A10 CTA was based on the percentage of live tumor cells stained at intensities of 0, 1+, 2+, or 3+. Most tested samples showed no expression of either target proteins. Figure S6E shows the P score (percentage of tumor cells stained at ≥2+) for both MAGE-A4 and MAGE-A10 in a selection of positively stained samples. Among the positively stained samples, the majority had expression of both MAGE-A4 and MAGE-A10 with similar levels. Four samples showed MAGE-A4 staining with a P score of 100 (with 3+ staining intensity in 70%–100% of tumor cells) but MAGE-A10 P score of 0, demonstrating the specificity of the anti–MAGE-A10 antibody, without cross-reactivity to MAGE-A4 (all the four data points overlapped and are shown as one in Figure S6E). Conversely, eight samples (Figure S6E, circled) showed P scores for MAGE-A10 between 20 and 100 (with 3+ staining intensity in 10%–70% of tumor cells) and no or very low staining for MAGE-A4 (P scores between 0 and 10). The low staining for MAGE-A4 observed in these samples could be due to actual low expression of MAGE-A4, but, even assuming that the MAGE-A4 signal is entirely due to cross-reactivity of the anti–MAGE-A4 antibody with MAGE-A10, this low cross-reactivity would not change the MAGE-A4 diagnosis status of these samples (positivity cutoff, ≥30% ≥2+), thus negating the risk of false positivity and confirming the diagnostic validity of the MAGE-A4 CTA.

In addition, anti–MAGE-A4 antibody staining by IHC showed no staining in Mel624 cell line, which is MAGE-A11 positive and MAGE-A12 positive by mRNA profile (Figure S6D). This further indicates the specificity of anti–MAGE-A4 antibody without cross-reactivity to MAGE-A11 and MAGE-A12.

MAGE-A4 prevalence

MAGE-A4 prevalence was assessed on a broad tissue sample set including a wide range of tumor indications (Table S1). For the determination of the prevalence of MAGE-A4 in different tumor indications, a MAGE-A4 cutoff of ≥30% at a ≥2+ intensity was applied. Feasibility of the MAGE-A4 assay was assessed in 316 tissue samples distributed over nine tumor indications covering the complete MAGE-A4 dynamic range (Figure S2, Table S1). Of the 316 tissue samples tested, 80 samples were positive for MAGE-A4. As demonstrated in Figure S2, the prevalence of MAGE-A4 ranged from 6% in EGJ cancer up to 67% in synovial sarcoma. Lower prevalence is observed in gastric cancer, EGJ cancer, MRCLS, ovarian cancer, and endometrium carcinoma, while in synovial sarcoma, HNSCC, and esophageal squamous cell carcinoma, and adenocarcinoma the prevalence is higher.

Robustness of the assay

To evaluate the robustness of the MAGE-A4 IHC CTA, precision testing (intra-run and inter-run), inter-lot (lot 1 vs. lot 2) and inter-lab (CellCarta BE vs. CellCarta US) comparison was evaluated to confirm the MAGE-A4 IHC CTA robustness regardless of the antibody lot used or the lab performing the MAGE-A4 assay. Each sample tested was stained for MAGE-A4 and their corresponding isotype control.

The MAGE-A4 IHC CTA robustness was evaluated both qualitatively and semi-quantitatively as scored by a pathologist. For each slide, the MAGE-A4 status (positivity cutoff: ≥30% tumor cells stained by MAGE-A4 at a ≥2+ intensity) was determined. The evaluation was based on the case status of each sample. All serial sections of each sample should be positive or negative in all the reads and an 80% overall concordance (overall percent agreement [OPA]) must be reached on slide level to consider the robustness as valid. The robustness results are summarized below.

Repeatability and reproducibility: precision

To evaluate the robustness (intra-run and inter-run) of the MAGE-A4 IHC assay, precision testing was performed in three independent staining runs on non-consecutive days on at least two Dako Link autostainer platforms by at least two different operators (Figure S7) to evaluate inter-run, intra-run, inter-operator, and inter-instrument variability. In each run, four serial sections were stained (three slides with the positive protocol and one with the negative protocol) from each block to evaluate the repeatability (intra-run) and reproducibility (inter-run) of the assay. Over three runs, 12 slides (nine with positive protocol and three with negative protocol) were stained per block.

Based on these results from the qualitative (Figures S8 and S9) and semi-quantitative (Table S2) evaluation, the precision of the MAGE-A4 assay was confirmed and each indication tested showed a concordance of >80%. Therefore, the MAGE-A4 assay was considered robust. Since different operators and instruments were used, the MAGE A4 assay is robust regardless of the operator or Dako Link autostainer instrument used for staining. MRCLS showed the lowest robustness (OPA 89%) since five slides from two samples deviated resulting in a different category. In the first sample, one slide was scored negative (22% at ≥2+ intensity), whereas the average score for this samples was 37% at ≥2+ intensity. In the second sample, four slides were scored negative (21%, 27%, 29%, and 29% at ≥2+ intensity), whereas the average score for this sample was 33% at ≥2+ intensity. Since the cutoff for MAGE-A4 is 30%, both samples were borderline cases, and all slides were scored around the cutoff. The variation on slide level was minimal (15% CV and 20% CV, respectively) but since categorization was used, this resulted in a different category.

Inter-lab variability

The MAGE-A4 assay was initially validated at CellCarta Antwerp (CC BE). After validation at CC BE, the MAGE-A4 assay was transferred to CellCarta Naperville (CC US). All of CellCarta's laboratories are CAP/CLIA certified. All staining platforms at all sites are cross validated twice per year.

For inter-lab variability between CellCarta Antwerp (CC BE) and CellCarta Naperville (CC US), two serial slides of 12 tissue samples (six sarcoma and six carcinoma samples) (Table S3) were stained at both labs and evaluated for concordance using the ≥30% positivity at a ≥2+ intensity cutoff.

As demonstrated in Table S3, one sample, a MRCLS, had a higher variability (CV 47% [2.53/5.36]) compared to the other samples (CV <10%) although the MAGE-A4 status remained unchanged. Biological variation between the two stained slides could lead to the observed variability of staining.

Based on the results, it has been concluded that the MAGE-A4 assay performs similarly regardless of the staining lab, CC BE or CC US. The robustness of the MAGE-A4 assay is therefore confirmed. Representative images of the inter-lab comparison are included in Figures S10–S13.

Inter-lot variability

To evaluate lot-to-lot variability, three different MAGE-A4 lots (A001, F001, and F002) were compared and evaluated for robustness. Lot-to-lot variability was tested on synovial sarcoma (5), MRCLS (4), melanoma (3), breast carcinoma (1), ovarian carcinoma (1), bladder carcinoma (1), lung carcinoma (1), laryngeal squamous cell carcinoma (1), and qualified batch run controls (MAGE-A4–positive and –negative cell pellet, normal tissue, and MAGE-A4–positive tumor sample). Serial slides of the tissue samples were stained with both lots and qualitatively and semi-quantitatively evaluated.

The results of inter-lot variability are presented in Figures S14–S16 and Table S4. In general, F001 showed a slightly weaker staining compared to A001 and F002. However, the scoring was not affected, and the lot-to-lot variability was considered valid.

Based on the results, it has been concluded that the MAGE-A4 assay performs similarly regardless of the MAGE-A4 antibody lot used for testing.

SUPPLEMENTAL TABLES

Table S1. MAGE-A4 prevalence data in commercially procured FFPE tissue carcinoma and sarcoma samples.

EGJ, esophagogastric junction; FFPE, formalin fixed paraffin embedded; MAGE-A4, melanoma-associated antigen A4.

Tumor indication	Tissue samples tested	Prevalence MAGE-A4 with 30% cutoff	Dynamic range % MAGE-A4 at 2+ and 3+ intensity		
			0%–25%	25%–35% (around the cutoff)	35%–100%
Synovial sarcoma	30	20/30 (67%)	10	1	19
Myxoid/round cell liposarcoma	31	4/31 (13%)	26	1	4
Ovarian	41	6/41 (15%)	33	3	5
Endometrium	37	6/37 (16%)	31	2	4
Gastric adenocarcinoma	34	5/34 (15%)	29	0	5
Esophageal squamous cell carcinoma	34	15/34 (44%)	19	2	13
EGJ	49	3/49 (6%)	46	1	2
Esophageal adenocarcinoma	30	6/30 (20%)	23	2	5
Head and neck squamous cell carcinoma	30	15/30 (50%)	15	0	15

Table S2. Tumor indications used for precision evaluation.

EGJ, esophagogastric junction carcinoma; HNSCC, head and neck squamous cell carcinoma; MAGE-A4, melanoma-associated antigen A4; MRCLS, myxoid/round cell liposarcoma.

Tumor indication	Number of samples tested for robustness	Overall concordance on slide level (total slide stained)	Dynamic range % MAGE-A4		
			0%–25%	25%–35% (around the cutoff)	35%–100%
Synovial sarcoma	5	100% (45/45)	1	2*	2
MRCLS	5	89% (40/45)	2	2*	1
Ovarian	10	100% (89/89)**	5	3	2
Endometrium carcinoma	5	100% (44/44)**	2	2	1
Esophageal adenocarcinoma	5	100% (45/45)	0	2	3
Esophageal squamous cell carcinoma	4	100% (36/36)	1	2	1
Gastric adenocarcinoma	5	100% (45/45)	2	0	3
HNSCC	9	100% (81/81)	2	3	4
EGJ	4	100% (36/36)	3	0	1
Mix of other solid tumors:<br>Bladder carcinoma<br>Melanoma<br>Lung carcinoma	 2<br>2<br>4	100% (72/72)	0	1	7

*One sample from synovial sarcoma (average % MAGE-A4 at ≥2+: 36%) and one sample from MRCLS (average % MAGE-A4 at ≥2+: 37%) were counted as samples around the cutoff in precision evaluation since their MAGE-A4 scores fell closely enough although not strictly within 25%–35%.

**One slide could not be evaluated.

Table S3. Results of MAGE-A4 CTA assay transfer.

BE, Belgium; CTA, clinical trial assay; HNSCC, head and neck squamous cell carcinoma; MAGE-A4, melanoma-associated antogen A4; MRCLS, myxoid/round cell liposarcoma; SD, standard deviation; US, United States.

Tumor indication (n° samples tested)	Results MAGE-A4			
	Average % at ≥2+ intensity	SD	Status	Concordance US/BE
Melanoma (2)	95.00	0.00	Positive	100% concordant
	10.00	0.00	Negative	
Ovarian (1)	30.00	0.00	Positive	
Synovial sarcoma (4)	0.00	0.00	Negative	
	0.00	0.00	Negative	
	91.00	2.02	Positive	
	44.42	4.36	Positive	
Bladder carcinoma (1)	60.00	0.00	Positive	
HNSCC (1)	100.00	0.00	Positive	
Lung carcinoma (1)	67.50	3.54	Positive	
MRCLS (2)	5.36*	2.53	Negative*	
	7.00	0.24	Negative	

*An MRCLS sample with higher variability (CV 47% [2.53/5.36)] in % MAGE-A4 positivity compared to the 11 other carcinoma samples.

Table S4. Inter-lot robustness of the MAGE-A4 antibody.

MAGE-A4, melanoma-associated antigen A4; MRCLS, myxoid/round cell liposarcoma.

MAGE-A4 antibody lot	Tumor type	% positive cells at ≥2+ intensity	MAGE-A4 status
**Lot A001 vs. F001**			
A001	Synovial sarcoma	46	Positive
F001		39	Positive
A001	Synovial sarcoma	96	Positive
F001		90	Positive
A001	Synovial sarcoma	0	Negative
F001		0	Negative
A001	MRCLS	0	Negative
F001		0	Negative
A001	MRCLS	29	Negative
F001		25	Negative
A001	MRCLS	47	Positive
F001		32	Positive
A001	Melanoma	13	Negative
F001		12	Negative
A001	Breast cancer	0	Negative
F001		0	Negative
A001	Esophageal cancer	92	Positive
F001		83	Positive
**Lot F001 vs. F002**			
F001	Melanoma	95	Positive
F002		95	Positive
F001	Ovarian cancer	25	Negative
F002		25	Negative
F001	Synovial sarcoma	0	Negative
F002		0	Negative
F001	Synovial sarcoma	0	Negative
F002		0	Negative
F001	Urinary bladder cancer	65	Positive
F002		50	Positive
F001	Laryngeal squamous cell carcinoma	100	Positive
F002		100	Positive
F001	Melanoma	15	Negative
F002		10	Negative
F001	Lung squamous cell carcinoma	65	Positive
F002		75	Positive

Table S5. Prevalence of HLA-A*02 alleles in different races and ethnicities in patients screened by the NMDP.[1,2]

API, Asian or Pacific Islander; NMDP, National Marrow Donnor Program.

Numbers represent the percentage of individuals expressing each allele or group of alleles in the population of interest using the formula Pi = 2 x F – (F^2), where Pi is the percentage of individuals expressing the allele and F is the allele frequency.

Allele	NMDP European Caucasian (N = 1,242,890)	NMDP African American (N = 416,518)	NMDP Mexican or Chicano (N = 261,235)	NMDP API (2007, N = 3,542)	NMDP Chinese (N = 99,672)	NMDP Japanese (N = 24,582)
A*02:01	47.51	23.17	37.51	18.02	18.03	27.41
A*02:02	0.18	8.11	1.51	0.06	0.02	0.01
A*02:03	0.004	0.04	0.04	6.22	14.88	0.28
A*02:06	0.36	0.14	3.92	9.42	6.86	14.40
A*02:01 + 02:02 + 02:03 + 02:06	47.90	30.41	41.80	31.90	37.12	39.82

References:

1. Gragert, L., Madbouly, A., Freeman, J., Maiers, M. (2013). Six-locus high resolution HLA haplotype frequencies derived from mixed-resolution DNA typing for the entire US donor registry. Hum. Immunol. *74*, 1313–1320. https://doi.org/10.1016/j.humimm.2013.06.025

2. Maiers, M., Gragert, L., Klitz, W. (2007). High-resolution HLA alleles and haplotypes in the United States population. Hum. Immunol. *68*, 779–788. https://doi.org/10.1016/j.humimm.2007.04.005

Table S6. MAGE-A4 prevalence reported in this study in comparison to previous literature reports.

EGJ, esophagogastric junction; HNSCC, head and neck squamous cell carcinoma; IHC, immunohistochemistry; MRCLS, myxoid/round cell liposarcoma; NSCLC, non-small cell lung cancer; RT-qPCR, quantitative reverse transcription polymerase chain reaction; SyS, synovial sarcoma.

Cancer	This study (ADP-0000-001/ADP-0044-002)			Literature reports			
	MAGE-A4+ (%)	Assay	Positivity cutoff	MAGE-A4+ (%)	Assay	Positivity cutoff	Reference
SyS	70% (140/201)	IHC	≥30%, ≥2+	82% (89/108)	IHC	Total score ≥3	1
				53% (9/17)	IHC	≥5%, ≥1+	2
MRCLS	40% (27/67)	IHC	≥30%, ≥2+	0% (0/9)	IHC	≥5%, ≥1+	2
				68% (63/93)	IHC	Total score ≥3	3
Urothelial	32% (30/93)	IHC	≥30%, ≥2+	64% (60/94)	IHC	>0%, ≥1+	4
				42% (175/418)	IHC	>0%, ≥1+	5
				19% (281/1522)	IHC	>0%, ≥1+	6
EGJ	26% (24/93)	IHC	≥30%, ≥2+				N/A
Ovarian	24% (54/226)	IHC	≥30%, ≥2+	42% (31/74)	IHC	≥5%, ≥1+	7
				36% (106/294)	IHC	≥5%, ≥1+	8
HNSCC	22% (43/200)	IHC	≥30%, ≥2+	72% (63/88)	IHC	>0%, ≥1+	9
				24% (12/51)	RT-qPCR	Ct ≤30	9
				60% (34/57)	RT-qPCR	≥1% reference	10
				38% (27/72)	RT-qPCR	>12.2 copies/10^4 GAPDH	11
Esophageal	21% (21/100)	IHC	≥30%, ≥2+	55% (124/226)	RT-qPCR	>12.2 copies/10^4 GAPDH	11
				7% (3/46)	Microarray	≥ 2-fold of normal tissue	12
				<20% (12/59)	IHC	>0%, ≥2+	12
Melanoma	16% (39/243)	IHC	≥30%, ≥2+	<10% (4/47)	IHC	>0%, ≥1+	13

				9% (53/586)	IHC	>0%, ≥1+	14
NSCLC	14% (63/457)	IHC	≥30%, ≥2+	30% (47/159)	IHC	H score ≥100	15
				18% (12/67)	RT-qPCR	>12.2 copies/10^4 GAPDH	11
Gastric	9% (6/70)	IHC	≥30%, ≥2+	35% (7/20)	RT-qPCR	>12.2 copies/10^4 GAPDH	11

References:

1. Iura, K., Maekawa, A., Kohashi, K., Ishii, T., Bekki, H., Otsuka, H., Yamada, Y., Yamamoto, H., Harimaya, K., Iwamoto, Y., et al. (2017). Cancer-testis antigen expression in synovial sarcoma: NY-ESO-1, PRAME, MAGEA4, and MAGEA1. Hum. Pathol. *61*, 130–139. https://doi.org/10.1016/j.humpath.2016.12.006

2. Kakimoto, T., Matsumine, A., Kageyama, S., Asanuma, K., Matsubara, T., Nakamura, T., Iino, T., Ikeda, H., Shiku, H., Sudo, A. (2019). Immunohistochemical expression and clinicopathological assessment of the cancer testis antigens NY-ESO-1 and MAGE-A4 in high-grade soft-tissue sarcoma. Oncol. Lett. *17*, 3937–3943. https://doi.org/10.3892/ol.2019.10044

3. Iura, K., Kohashi, K., Ishii, T., Maekawa, A., Bekki, H., Otsuka, H., Yamada, Y., Yamamoto, H., Matsumoto, Y., Iwamoto, Y., et al. (2017). MAGEA4 expression in bone and soft tissue tumors: its utility as a target for immunotherapy and diagnostic marker combined with NY-ESO-1. Virchows Arch. *471*, 383–392. https://doi.org/10.1007/s00428-017-2206-z

4. Sharma, P., Shen, Y., Wen, S., Bajorin, D.F., Reuter, V.E., Old, L.J., Jungbluth, A.A. (2006). Cancer-testis antigens: expression and correlation with survival in human urothelial carcinoma. Clin. Cancer Res. *12*, 5442–5447. https://doi.org/10.1158/1078-0432.CCR-06-0527

5. Bergeron, A., Picard, V., LaRue, H., Harel, F., Hovington, H., Lacombe, L., Fradet, Y. (2009). High frequency of MAGE-A4 and MAGE-A9 expression in high-risk bladder cancer. Int. J. Cancer *125*, 1365–1371. https://doi.org/10.1002/ijc.24503

6. Kocher, T., Zheng, M., Bolli, M., Simon, R., Forster, T., Schultz-Thater, E., Remmel, E., Noppen, C., Schmid, U., Ackermann, D., et al. (2002). Prognostic relevance of MAGE-A4 tumor antigen expression in transitional cell carcinoma of the urinary bladder: a tissue microarray study. Int. J. Cancer *100*, 702–705. https://doi.org/10.1002/ijc.10540

7. Yakirevich, E., Sabo, E., Lavie, O., Mazareb, S., Spagnoli, G.C., Resnick, M.B. (2003). Expression of the MAGE-A4 and NY-ESO-1 cancer-testis antigens in serous ovarian neoplasms. Clin. Cancer Res. *9*, 6453–6460.

8. Daudi, S., Eng, K.H., Mhawech-Fauceglia, P., Morrison, C., Miliotto, A., Beck, A., Matsuzaki, J., Tsuji, T., Groman, A., Gnjatic, S., et al. (2014). Expression and immune responses to MAGE antigens predict survival in epithelial ovarian cancer. PLoS One *9*, e104099. https://doi.org/10.1371/journal.pone.0104099

9. Baran, C.A., Agaimy, A., Wehrhan, F., Weber, M., Hille, V., Brunner, K., Wickenhauser, C., Siebolts, U., Nkenke, E., Kesting, M., et al. (2019). MAGE-A expression in oral and laryngeal leukoplakia predicts malignant transformation. Mod. Pathol. *32*, 1068–1081. https://doi.org/10.1038/s41379-019-0253-5

10. Cuffel, C., Rivals, J.P., Zaugg, Y., Salvi, S., Seelentag, W., Speiser, D.E., Lienard, D., Monnier, P., Romero, P., Bron, L., et al. (2011). Pattern and clinical significance of cancer-testis gene expression in head and neck squamous cell carcinoma. Int. J. Cancer *128*, 2625–2634. https://doi.org/10.1002/ijc.25607

11. Ishihara, M., Kageyama, S., Miyahara, Y., Ishikawa, T., Ueda, S., Soga, N., Naota, H., Mukai, K., Harada, N., Ikeda, H., et al. (2020). MAGE-A4, NY-ESO-1 and SAGE mRNA expression rates and co-expression relationships in solid tumours. BMC Cancer *20*, 606. https://doi.org/10.1186/s12885-020-07098-4

12. Lin, J., Lin, L., Thomas, D.G., Greenson, J.K., Giordano, T.J., Robinson, G.S., Barve, R.A., Weishaar, F.A., Taylor, J.M., Orringer, M.B., et al. (2004). Melanoma-associated antigens in esophageal adenocarcinoma: identification of novel MAGE-A10 splice variants. Clin. Cancer Res. *10*, 5708–5716. https://doi.org/10.1158/1078-0432.CCR-04-0468

13. Errington, J.A., Conway, R.M., Walsh-Conway, N., Browning, J., Freyer, C., Cebon, J., Madigan, M.C. (2012). Expression of cancer-testis antigens (MAGE-A1, MAGE-A3/6, MAGE-A4, MAGE-C1 and NY-ESO-1) in primary human uveal and conjunctival melanoma. Br. J. Ophthalmol. *96*, 451–458. https://doi.org/10.1136/bjophthalmol-2011-300432

14. Barrow, C., Browning, J., MacGregor, D., Davis, I.D., Sturrock, S., Jungbluth, A.A., Cebon, J. (2006). Tumor antigen expression in melanoma varies according to antigen and stage. Clin. Cancer Res. *12*, 764–771. https://doi.org/10.1158/1078-0432.CCR-05-1544

15. Su, C., Xu, Y., Li, X., Ren, S., Zhao, C., Hou, L., Ye, Z., Zhou, C. (2015). Predictive and prognostic effect of CD133 and cancer-testis antigens in stage Ib-IIIA non-small cell lung cancer. Int. J. Clin. Exp. Pathol. *8*, 5509–5518.

SUPPLEMENTAL FIGURES

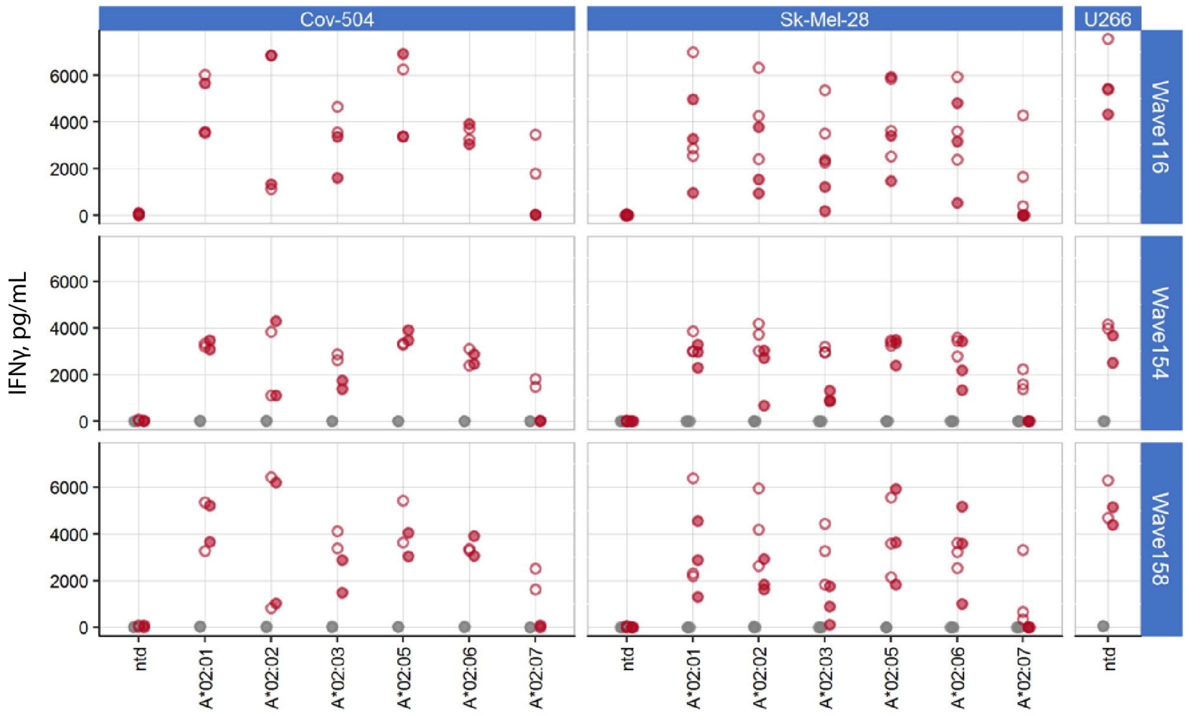

Figure S1. IFNγ release by afami-cel in response to MAGE-A4–positive cell lines expressing different HLA-A2 subtypes.

Cov-504 and Sk-Mel-28 (MAGE-A4 positive, HLA-A2 negative) cells were transduced to express HLA-A*02:01, HLA-A*02:02, HLA-A*02:03, HLA-A*02:05, HLA-A*02:06 and HLA-A*02:07 and subsequently used as targets for three separate batches of afami-cel (red points) or donor-matched non-transduced T cells (gray points; not available for Wave116). The natively HLA-A*02:01–positive and MAGE-A4–positive cell line U266 was included as a positive control. Closed circles show recognition (IFNγ release pg/ml) of endogenous MAGE-A4, open circles show recognition of cell lines exogenously loaded with 10^{-5} M synthetic MAGE-A4$_{230\text{-}239}$ peptide (IFNγ release pg/ml). Data are facetted vertically by T-cell donor. HLA, human leukocyte antigen; IFN, interferon; MAGE-A4, melanoma-associated antigen A4; ntd, non-transduced.

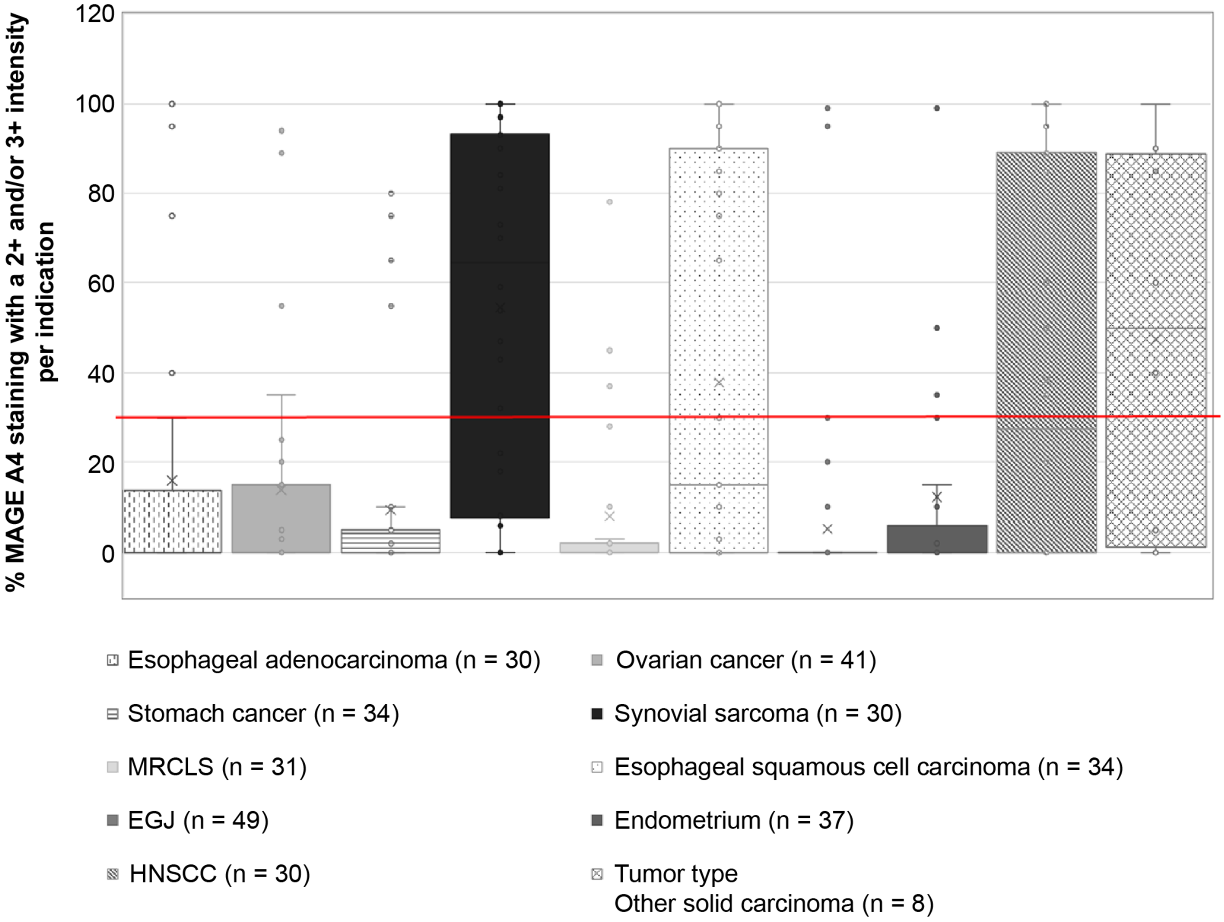

Figure S2. Distribution of the % MAGE-A4 positivity per tumor indication. Cutoff for MAGE-A4 positivity is indicated by a red horizontal line at 30%. The number of samples tested is indicated between brackets (n). EGJ, esophagogastric junction carcinoma; HNSCC, head and neck squamous cell carcinoma; MAGE-A4, melanoma-associated antigen A4; MRCLS, myxoid/round cell liposarcoma.

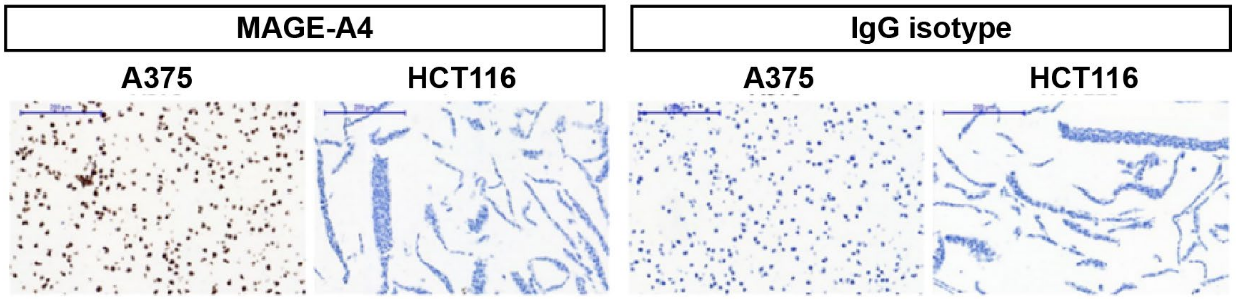

Figure S3. MAGE-A4 expression in control cell lines A375 (positive) and HCT116 (negative).

IgG, immunoglobulin G; MAGE-A4, melanoma-associated antigen A4.

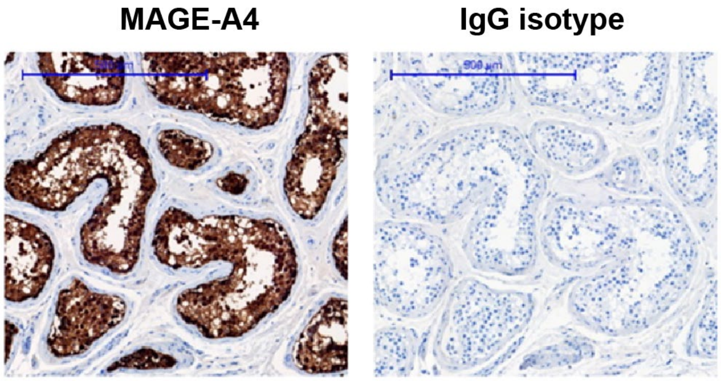

Figure S4. MAGE-A4 expression in control tissue (testis).

IgG, immunoglobulin G; MAGE-A4, melanoma-associated antigen A4.

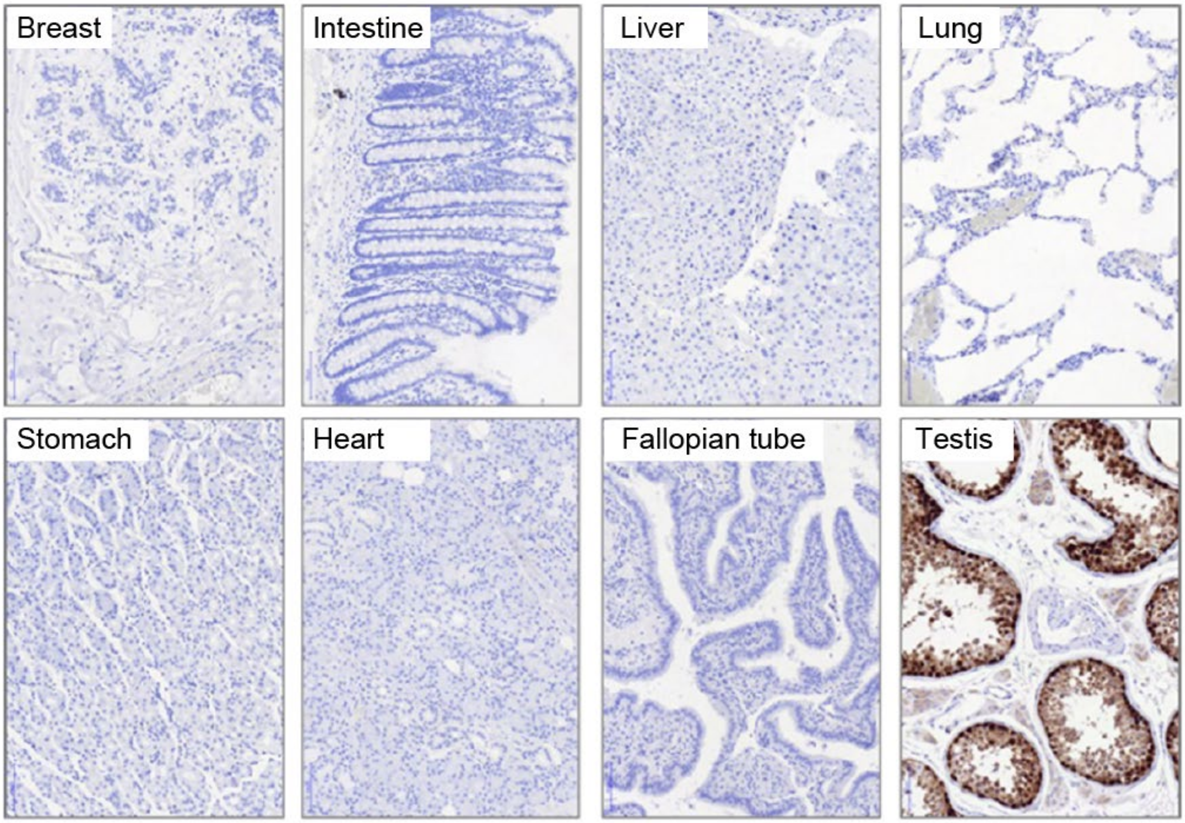

Figure S5. MAGE-A4 expression in normal tissues.

MAGE-A4, melanoma-associated antigen A4.

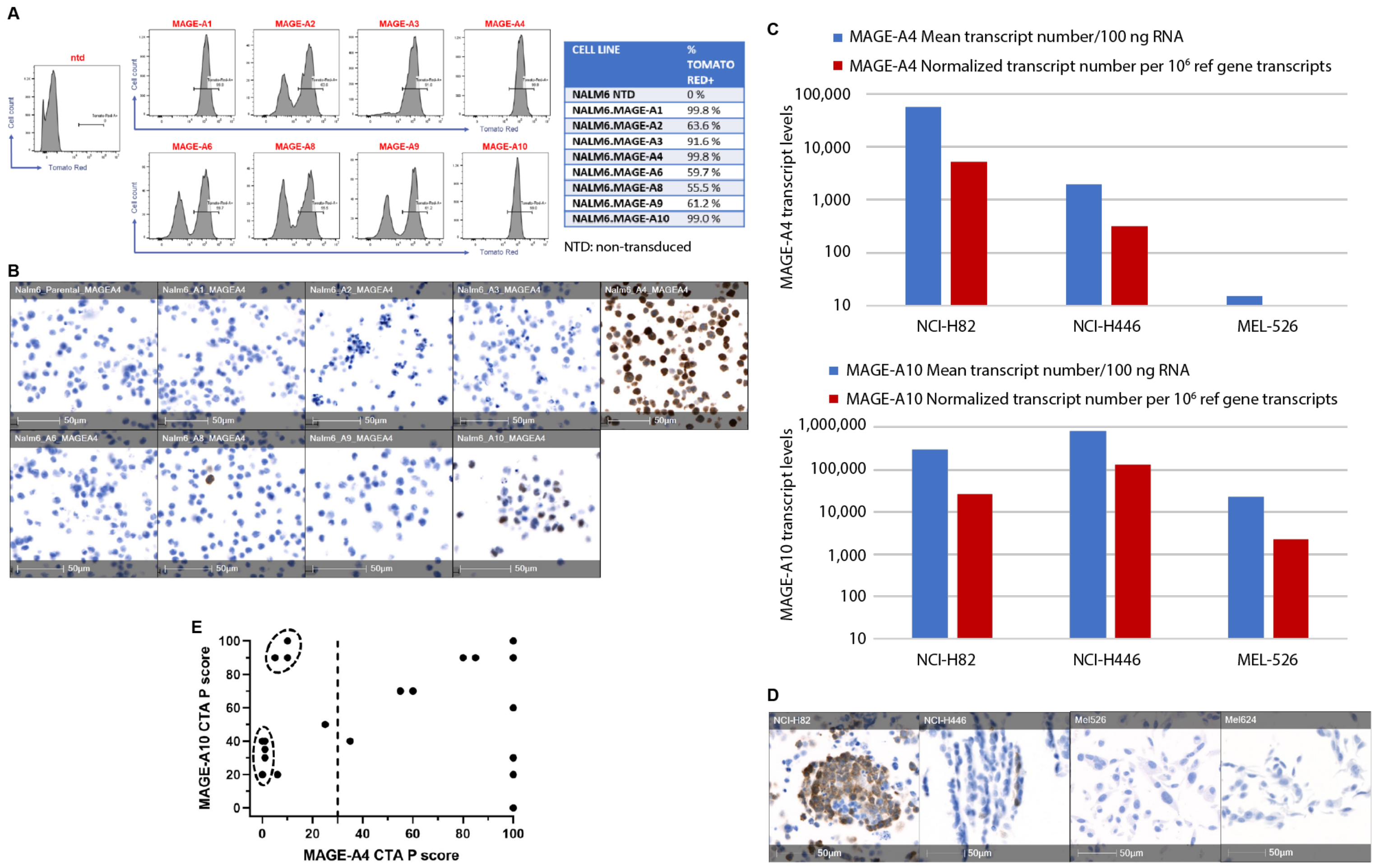

Figure S6. Anti–MAGE-A4 antibody specificity.

(A) MAGE-As expression in MAGE-As transduced NALM6 cell lines as determined by flow cytometry detection of the reporter protein Tomato Red. (B) IHC staining with anti–MAGE-A4 antibody of transduced NALM6 cell lines expressing various MAGE-As (A1, A2, A3, A4, A6, A8, A9 ,and A10). (C) MAGE-A4 and MAGE-A10 expression in NCI-H82, NCI-H466, and Mel526 cell lines as determined by qRT-PCR. (D) IHC staining with anti–MAGE-A4 antibody of NCI-H82 (MAGE-A4 high/MAGE-A10 high), NCI-H466 (MAGE-A4 low/MAGE-A10 high), Mel526 (MAGE-A4 negligible/MAGE-A10 high), and Mel624 (MAGE-A11+, MAGE-A12+) cell lines. (E) Tumor tissues stained and scored by both MAGE-A4 IHC CTA and MAGE-A10 IHC CTA. Dash vertical line showed the MAGE-A4 positivity cutoff (≥30% staining at ≥2+ intensities). The circled samples were highlighted to illustrate the negligible impact of MAGE-A10 expression on the determination of MAGE-A4 diagnosis. CTA, clinical trial assay; IHC, immunohistochemistry; MAGE-A4, melanoma-associated antigen A4; ntd, non-transduced; RT-PCR, reverse transcription polymerase chain reaction.

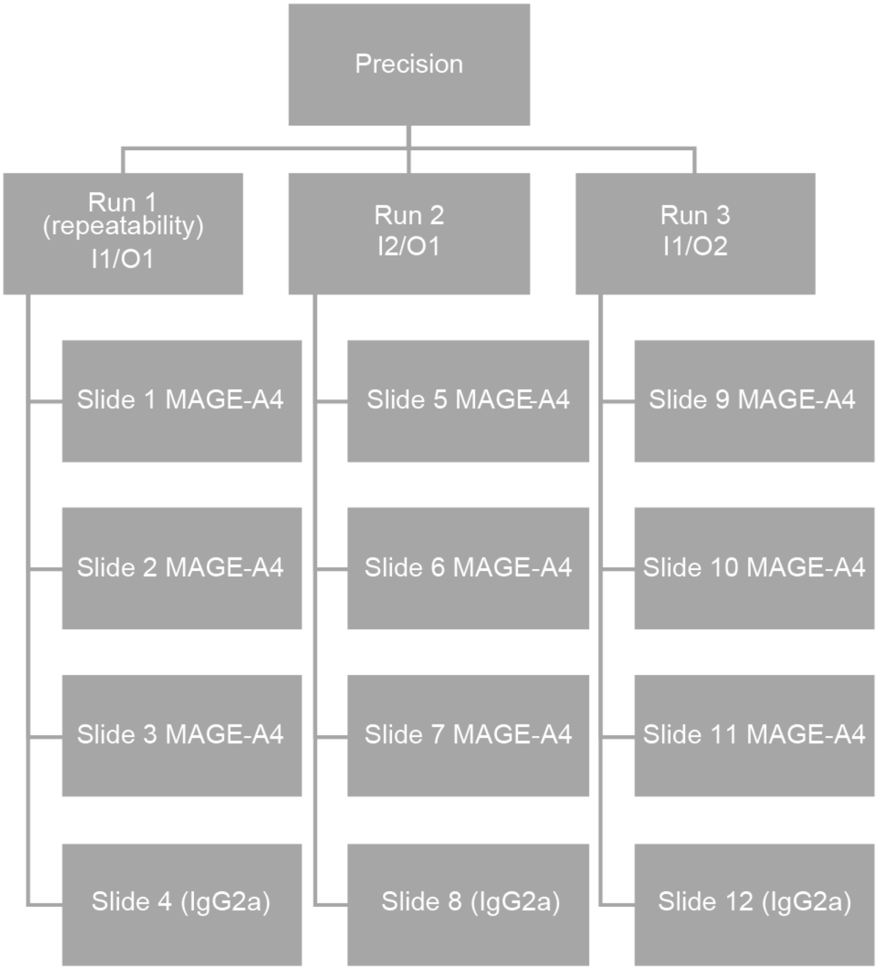

Figure S7. Diagram illustrating the robustness assessment strategy.

IgG, immunoglobulin G; MAGE A4, melanoma-associated antigen A4.

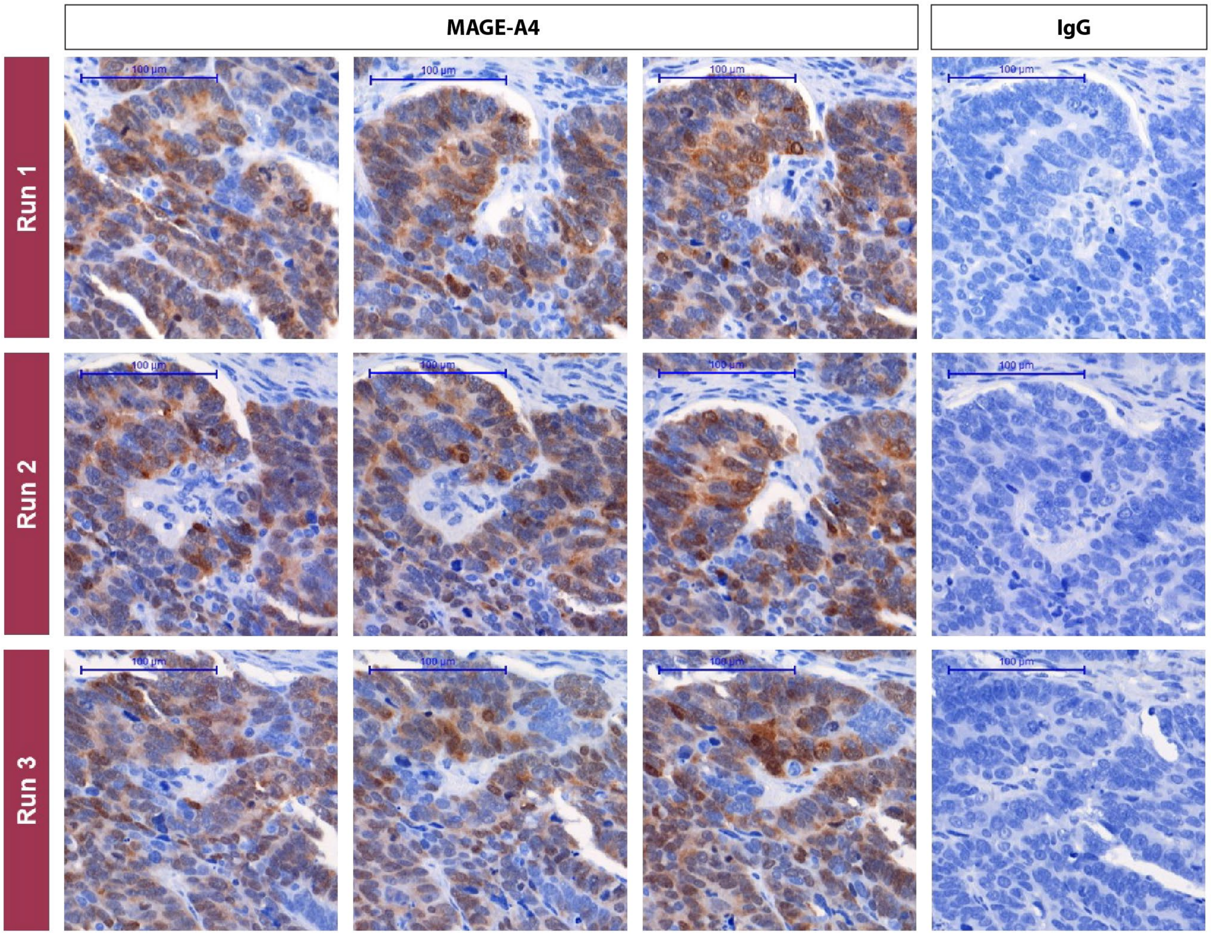

Figure S8. Representative detail images of the precision assessment of MAGE-A4 on a negative ovarian cancer tissue with 20% MAGE-A4 positivity at ≥2+ intensity during sensitivity screening.

Three serial sections of each sample were stained in each run for MAGE-A4. In each run, one slide was stained for the isotype IgG control. Repeatability was assessed in run 1, reproducibility was evaluated between the three different runs. 10x magnification. IgG, immunoglobulin G; MAGE-A4, melanoma-associated antigen A4.

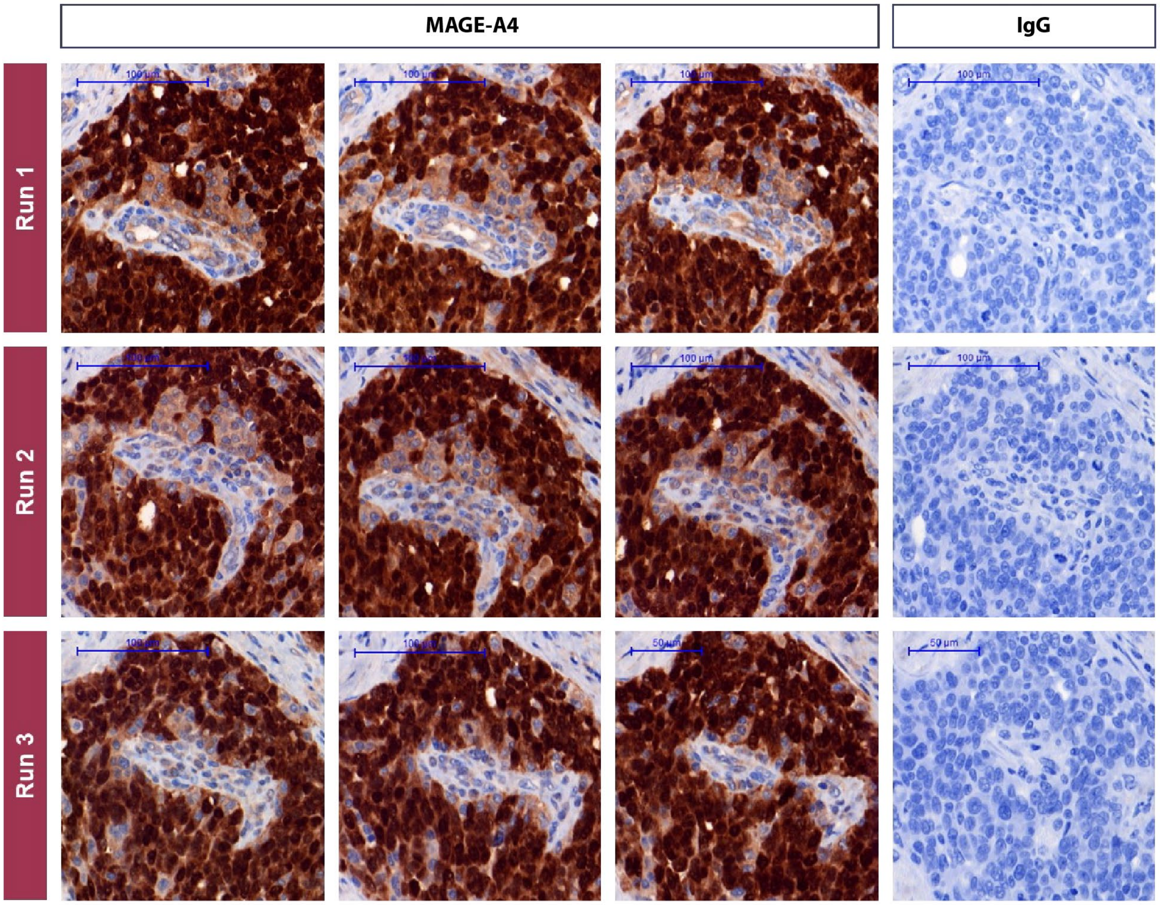

Figure S9. Representative detail images of the precision assessment of MAGE-A4 on a positive endometrium cancer tissue with a 94% MAGE-A4 positivity at ≥2+ intensity during sensitivity screening.

Three serial sections of each sample were stained in each run for MAGE-A4. In each run, one slide was stained for the isotype IgG control. Repeatability was assessed in run 1, reproducibility was evaluated between the three different runs. 20x magnification. IgG, immunoglobulin G; MAGE-A4, melanoma-associated antigen A4.

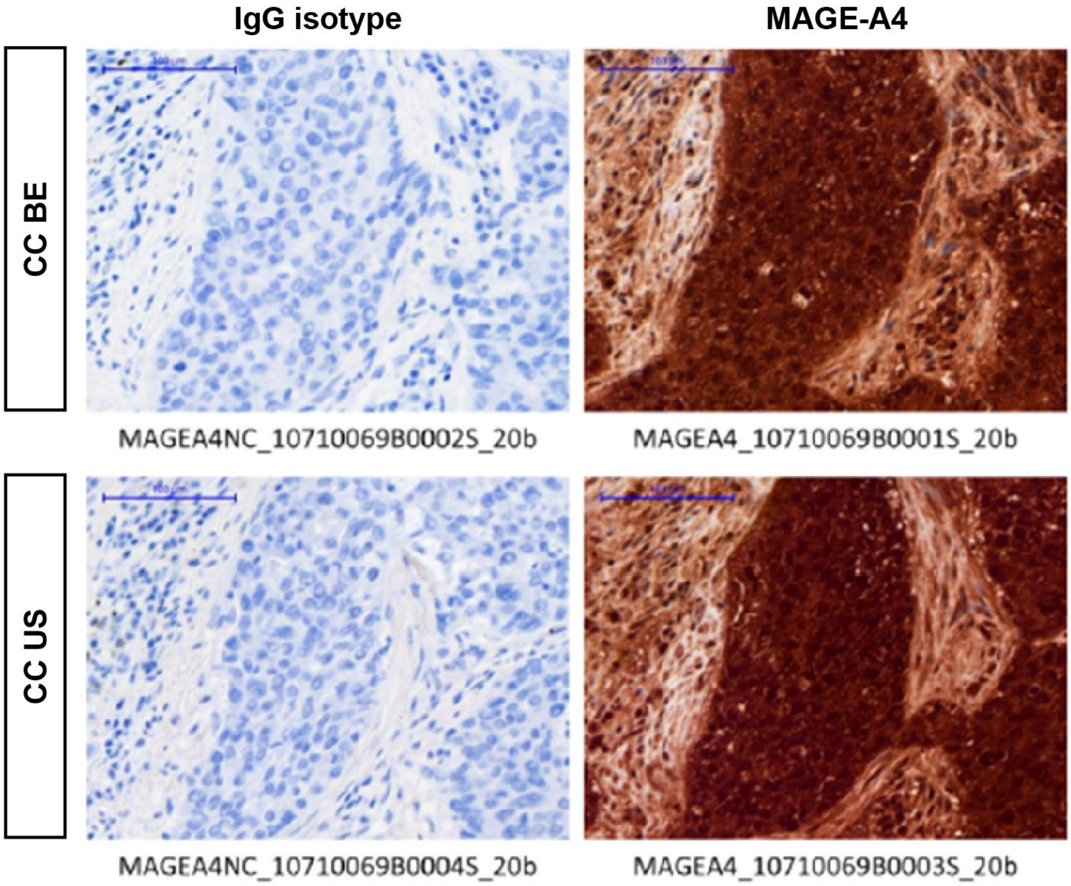

Figure S10. Representative images from lung squamous cell carcinoma stained at CC BE (top) and CC US (bottom) with the MAGE-A4 primary antibody (right) and the IgG isotype control (left).

BE, Belgium; CC, CellCarta, IgG, immunoglobulin G; MAGE-A4, melanoma-associated antigen A4; US, United States.

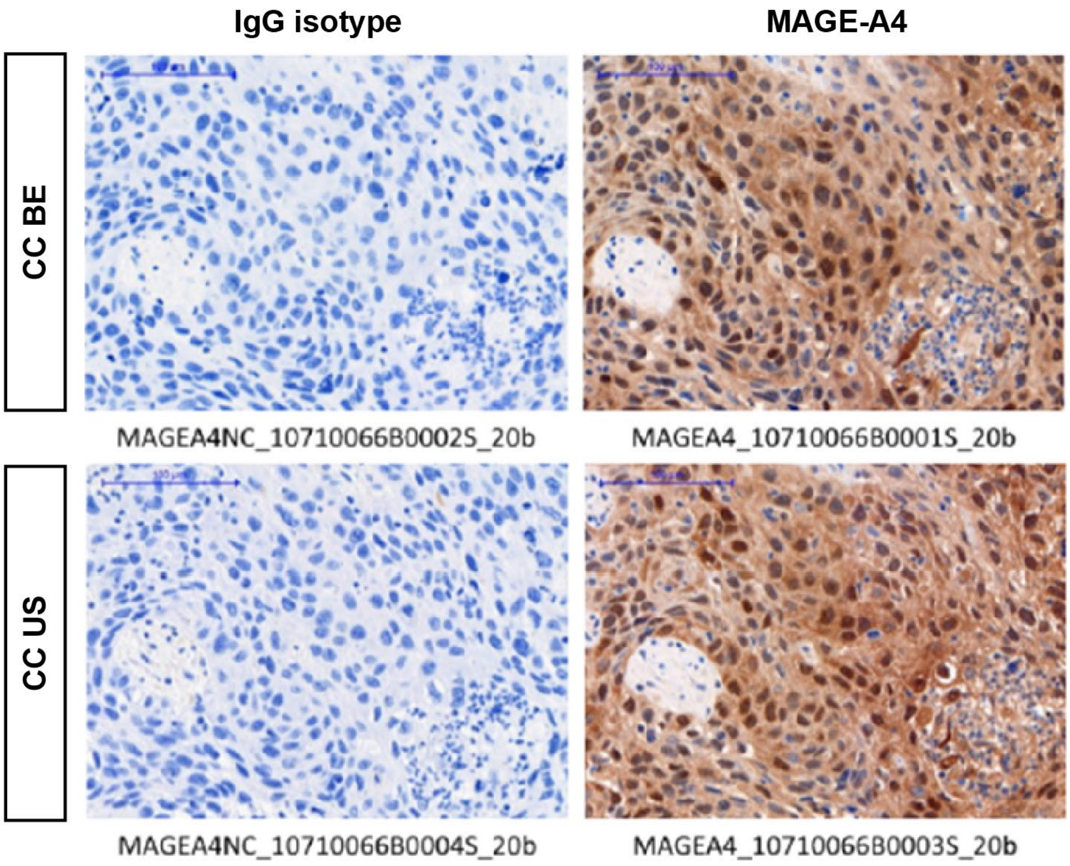

Figure S11. Representative images from urinary bladder cancer stained at CC BE (top) and CC US (bottom) with the MAGE-A4 primary antibody (right) and the IgG isotype control (left).

BE, Belgium; CC, CellCarta, IgG, immunoglobulin G; MAGE-A4, melanoma-associated antigen A4; US, United States.

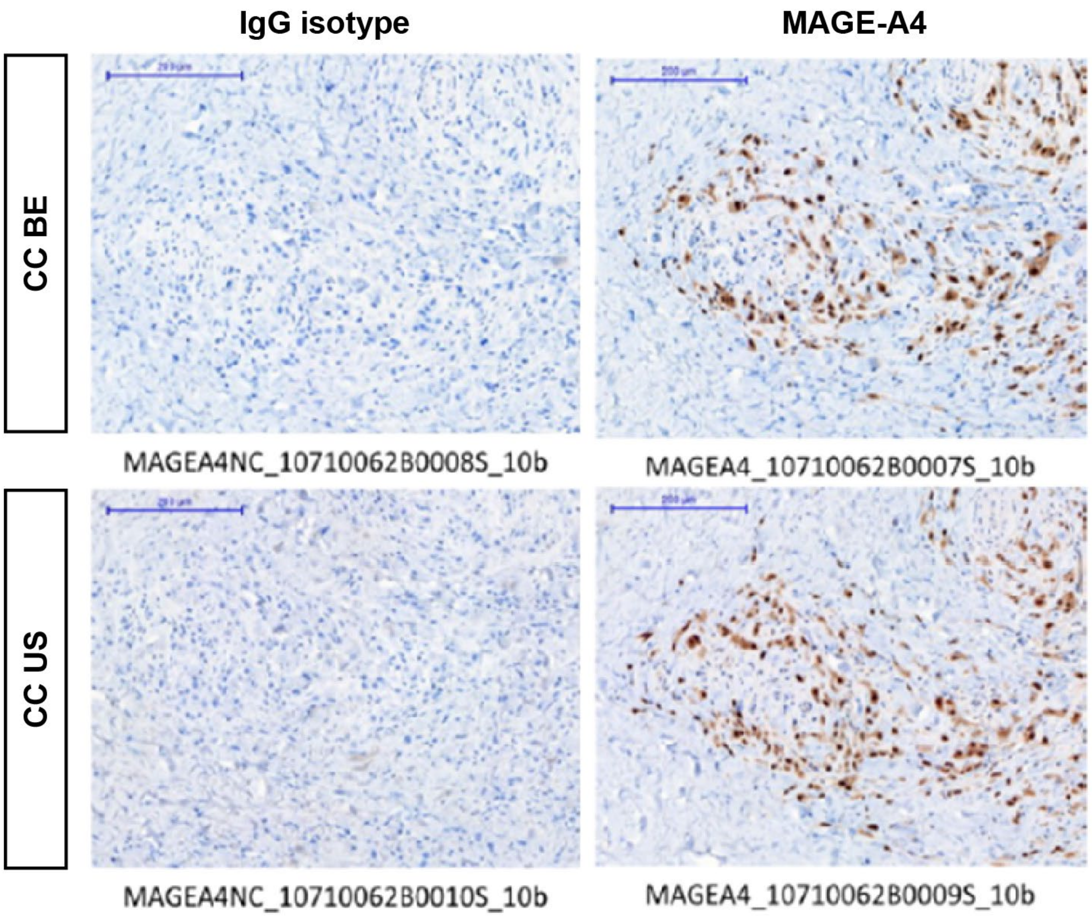

Figure S12. Representative images from melanoma cancer stained at CC BE (top) and CC US (bottom) with the MAGE-A4 primary antibody (right) and the IgG isotype control (left).

BE, Belgium; CC, CellCarta, IgG, immunoglobulin G; MAGE-A4, melanoma-associated antigen A4; US, United States.

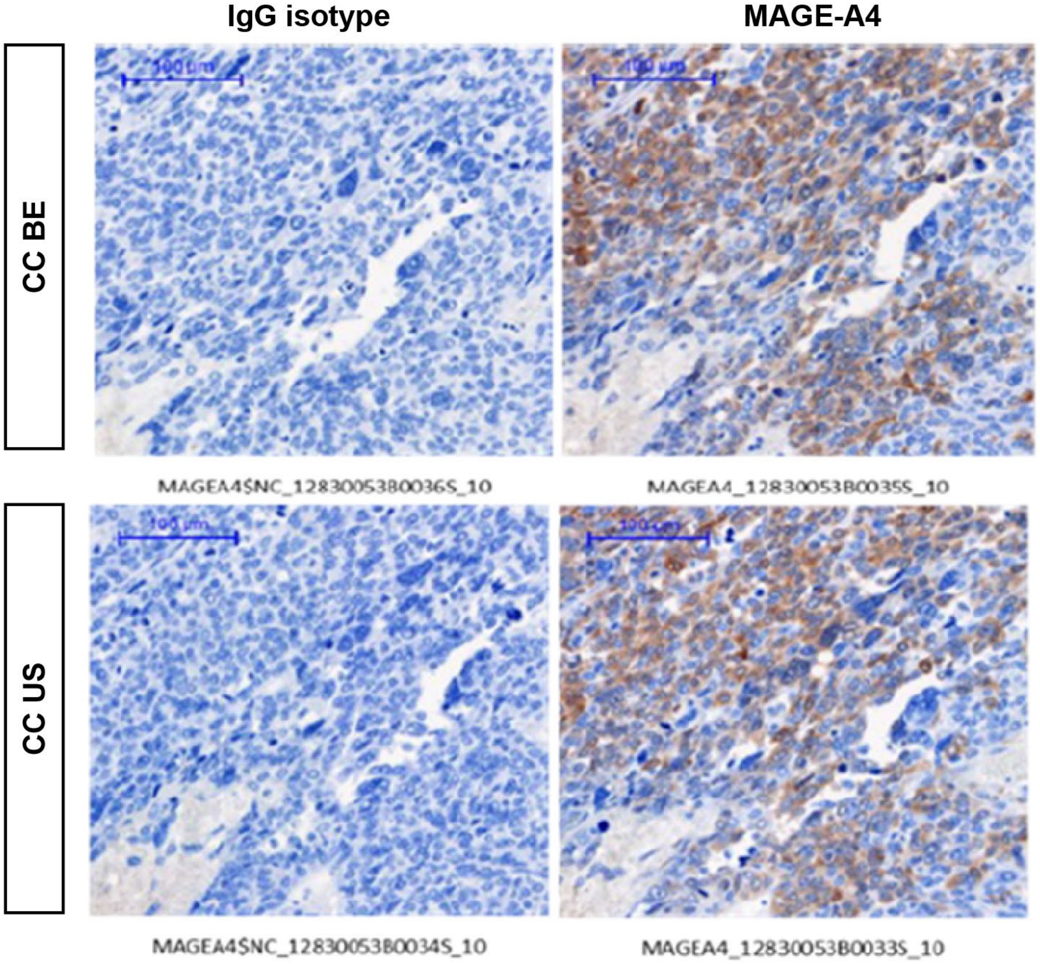

Figure S13. Representative images from myxoid/round cell liposarcoma stained at CC BE (top) and CC US (bottom) with the MAGE-A4 primary antibody (right) and the IgG isotype control (left).

BE, Belgium; CC, CellCarta, IgG, immunoglobulin G; MAGE-A4, melanoma-associated antigen A4; US, United States.

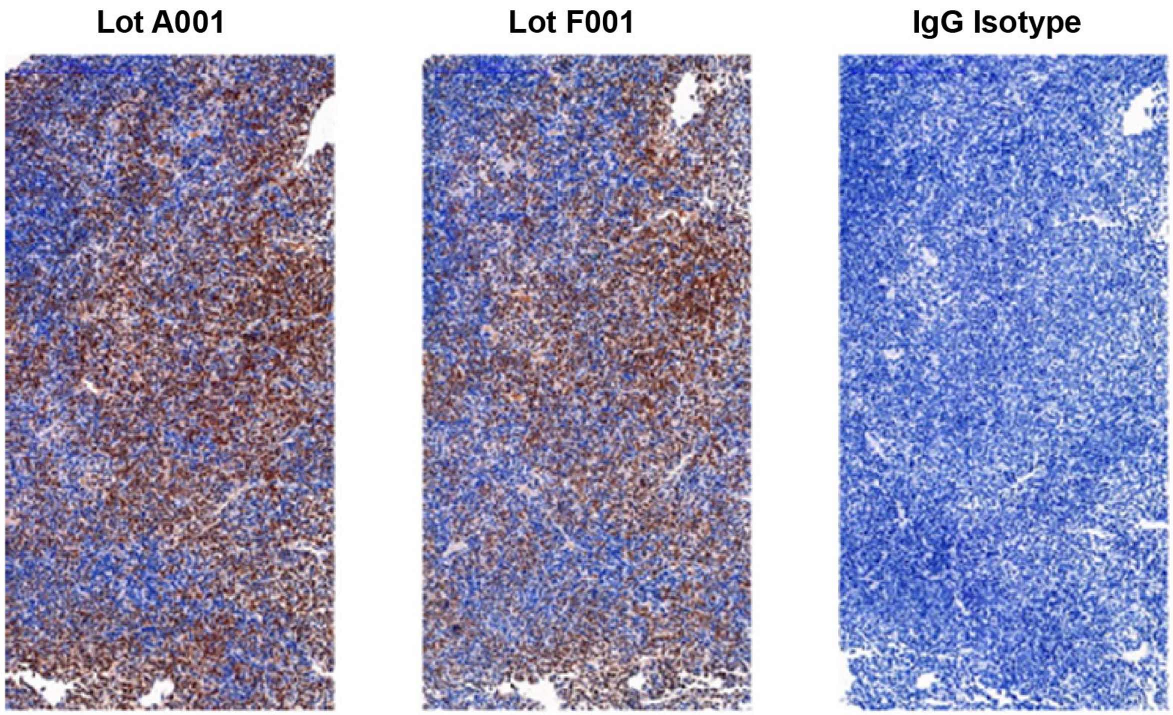

Figure S14. Serial slides of synovial sarcoma stained for MAGE-A4 using different antibody lots (A001 and F001).

No immunoreactivity is detected in the negative IgG controls. IgG, immunoglobulin G; MAGE-A4, melanoma-associated antigen A4.

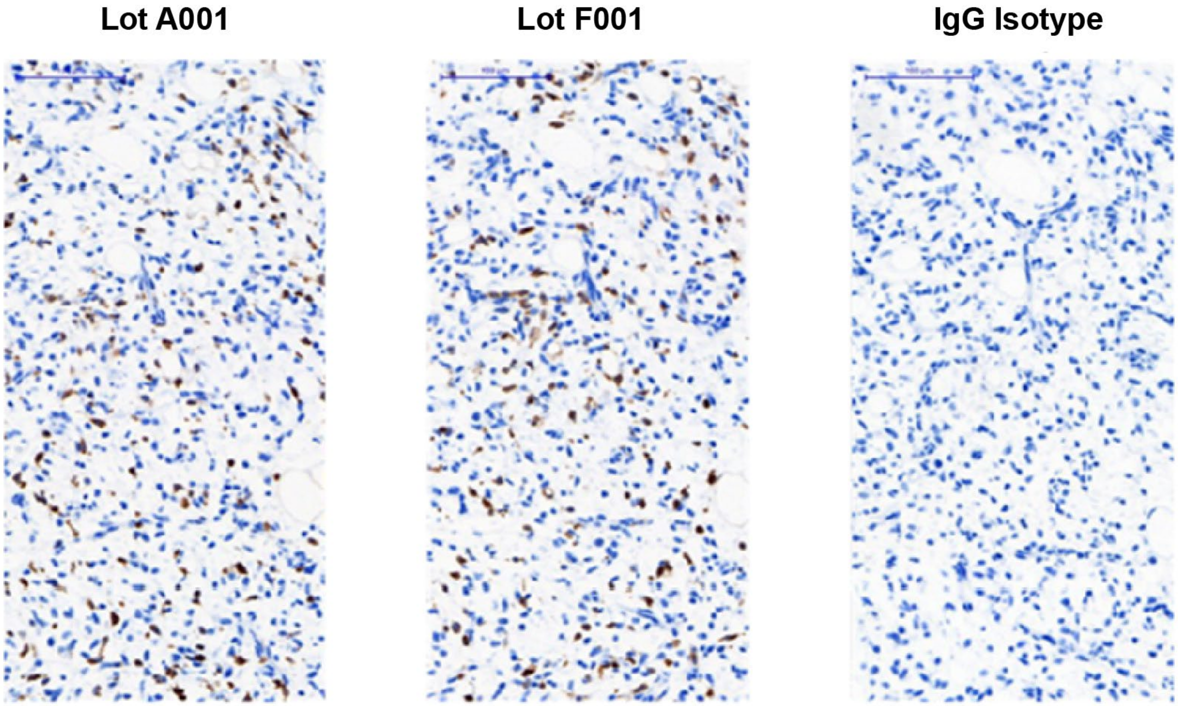

Figure S15. Serial slides of MRCLS stained for MAGE-A4 using different antibody lots (A001 and F001).

No immunoreactivity is detected in the negative IgG controls. IgG, immunoglobulin G; MAGE-A4, melanoma-associated antigen A4; MRCLS, myxoid/round cell liposarcoma.

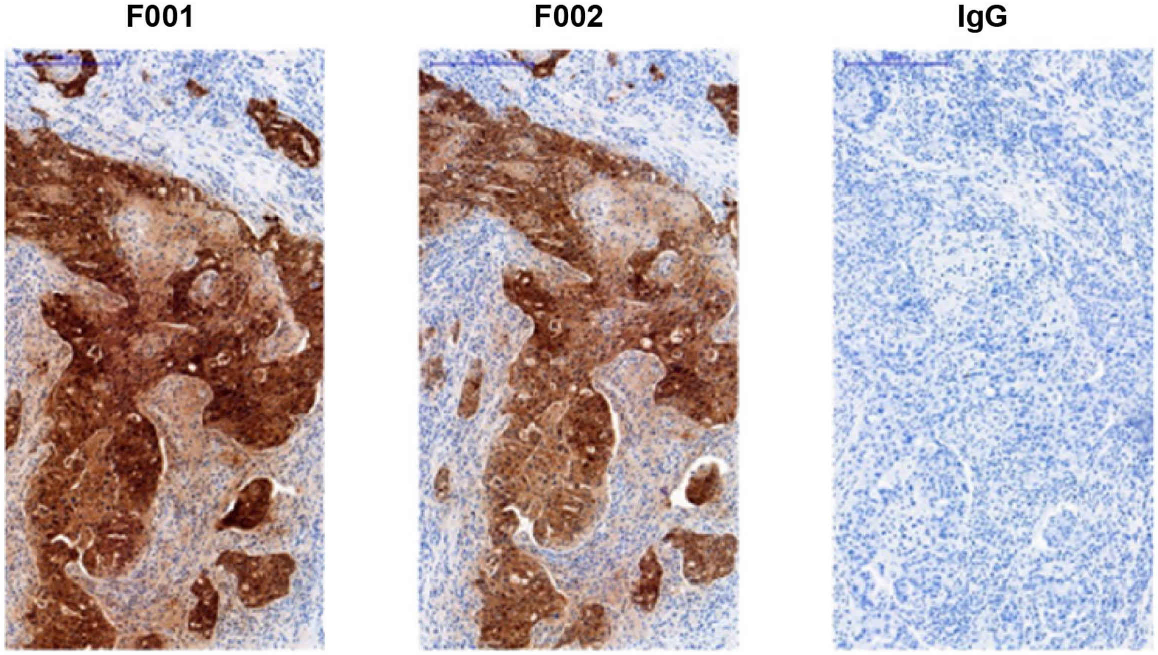

Figure S16. Serial slides of ovarian cancer stained for MAGE-A4 using different antibody lots (F001 and F002).

No immunoreactivity is detected in the negative IgG controls. IgG, immunoglobulin; MAGE-A4, melanoma-associated antigen A4.

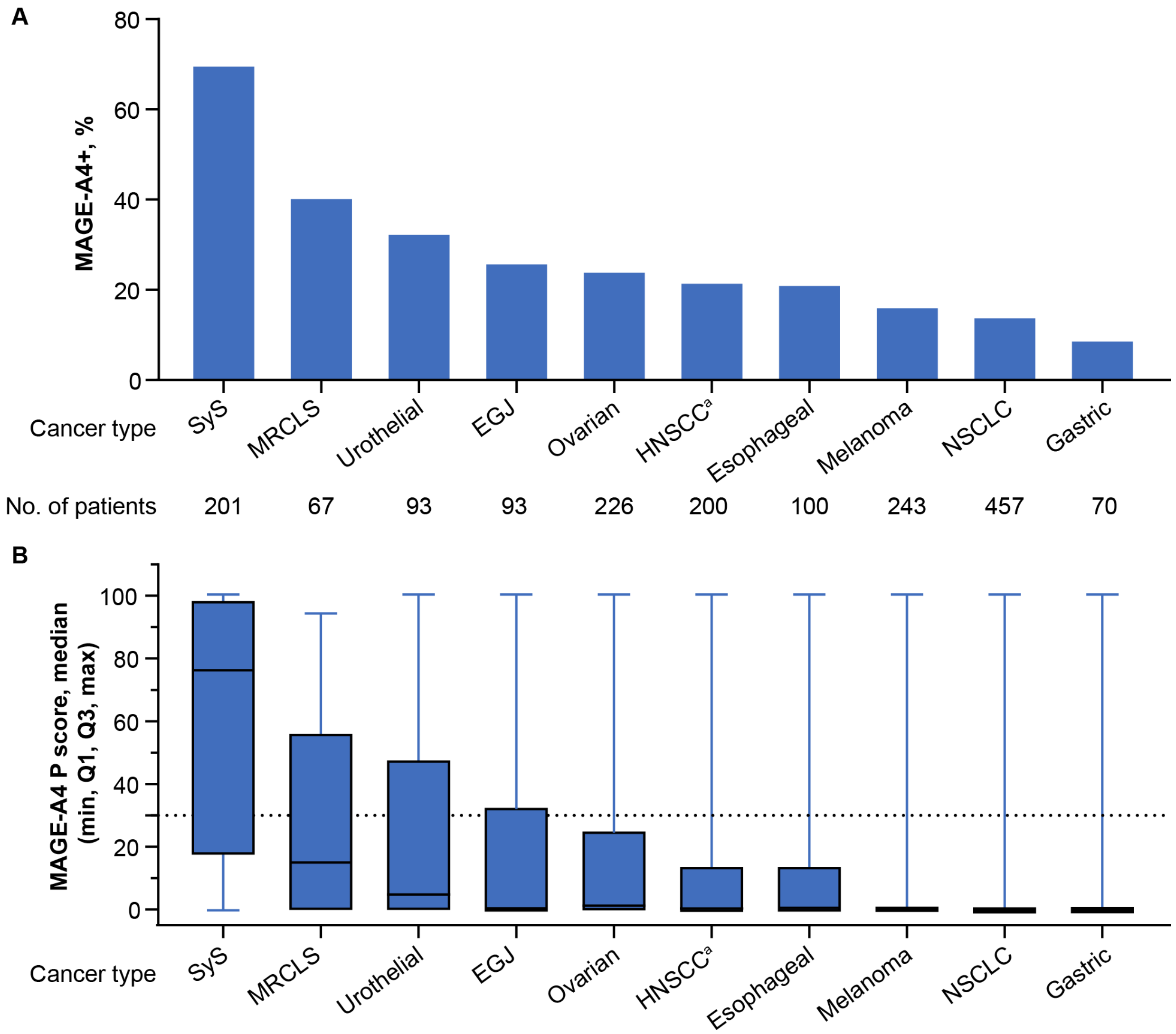

Figure S17. MAGE-A4 positivity and expression level by cancer type.

(A) MAGE-A4 positivity. (B) MAGE-A4 expression level. [a]n = 199 HNSCC, n = 1 “other” head and neck cancer histology. The dotted line represents the cutoff value of the P score indicating MAGE-A4 positivity. EGJ, esophagogastric junction cancer; HNSCC, head and neck squamous cell carcinoma; MAGE-A4, melanoma-associated antigen A4; MRCLS, myxoid/round cell liposarcoma; NSCLC, non-small cell lung cancer; P score, protein score; SyS, synovial sarcoma.

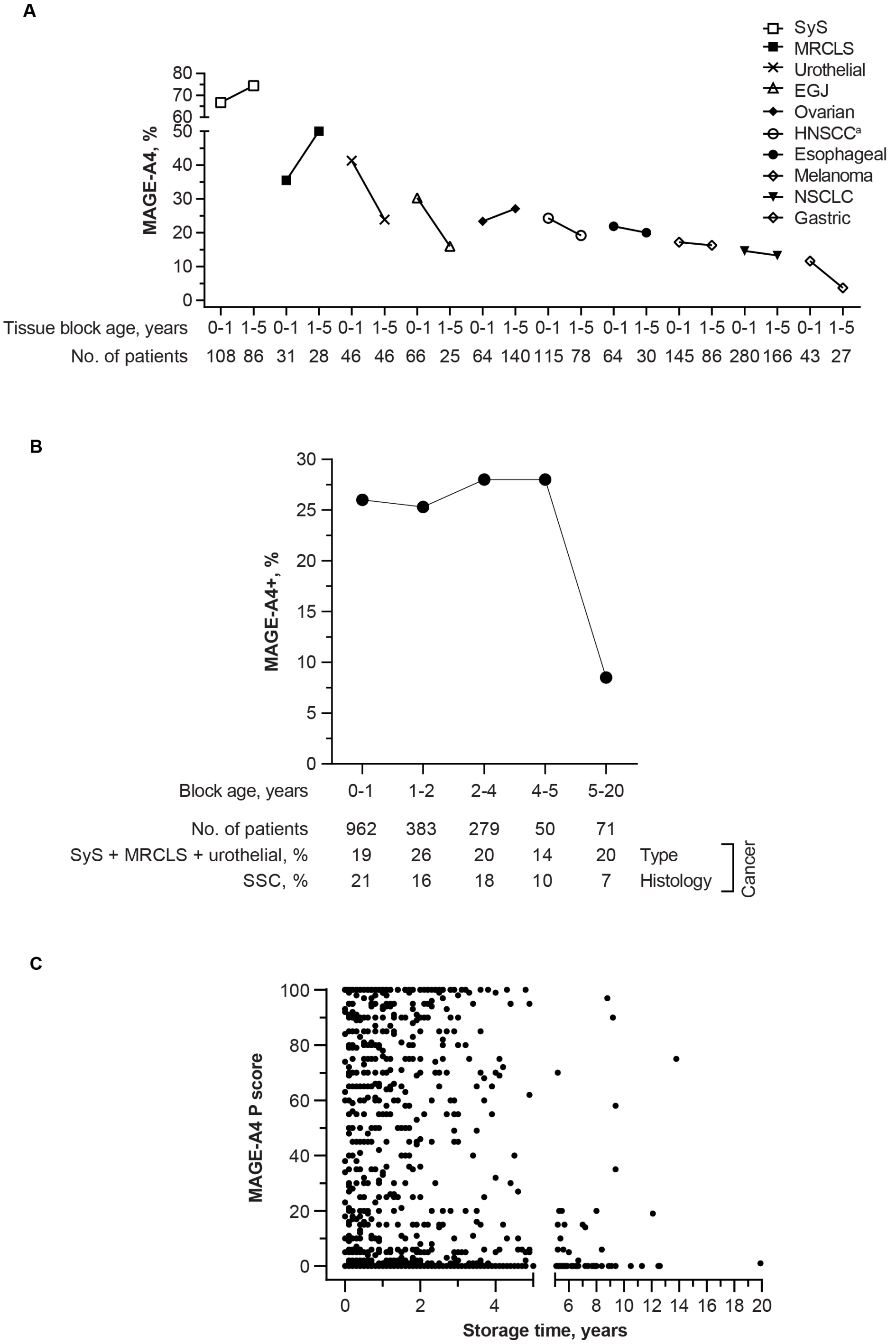

Figure S18. Effect of FFPE tumor sample storage time on MAGE-A4 positivity across cancer types.

(A) Storage time on MAGE-A4 positivity for individual cancer types. (B) Block age on MAGE-A4 positivity for all samples overall. (C) FFPE storage time on MAGE-A4 P score for all samples. [a]n = 199 HNSCC, n = 1 “other” head and neck cancer histology. EGJ, esophagogastric junction cancer; FFPE, formalin-fixed, paraffin-embedded; HNSCC, head and neck squamous cell carcinoma; MAGE-A4, melanoma-associated antigen A4; MRCLS, myxoid/round cell liposarcoma; NSCLC, non-small cell lung cancer; P score, protein score; SCC, squamous cell carcinoma; SyS, synovial sarcoma.
