# Supplementary material for: Identifying MAGE-A4-positive tumors for TCR T cell therapies in HLA-A∗02-eligible patients
Source: Mol Ther Methods Clin Dev. 2024 May 14;32(2):101265. doi: 10.1016/j.omtm.2024.101265 (PMC11170170; doi:10.1016/j.omtm.2024.101265)
Supplement: Document S2. Article plus supplemental information [file mmc2.pdf]

Molecular Therapy
Methods & Clinical Development
Original Article

Identifying MAGE-A4-positive tumors for TCR T cell therapies in HLA-A*02-eligible patients

Tianjiao Wang,[1,21] Jean-Marc Navenot,[1,21] Stavros Rafail,[2] Cynthia Kurtis,[1] Mark Carroll,[3] Marian Van Kerckhoven,[4] Sofie Van Rossom,[4] Kelly Schats,[4] Konstantinos Avraam,[5] Robyn Broad,[6] Karen Howe,[7] Ashley Liddle,[6] Amber Clayton,[7] Ruoxi Wang,[3] Laura Quinn,[8] Joseph P. Sanderson,[8] Cheryl McAlpine,[6] Carly Carozza,[9] Eric Pimpinella,[9] Susan Hsu,[9] Francine Brophy,[10] Erica Elefant,[10] Paige Bayer,[10] Dennis Williams,[11] Marcus O. Butler,[12] Jeffrey M. Clarke,[13] Justin F. Gainor,[14] Ramaswamy Govindan,[15] Victor Moreno,[16] Melissa Johnson,[17] Janet Tu,[18] David S. Hong,[19,22] and George R. Blumenschein, Jr.[20,22]

[1]Clinical Biomarkers & Companion Diagnostics, Adaptimmune, Philadelphia, PA, USA; [2]Biomarker Discovery and Platform, Adaptimmune, Philadelphia, PA, USA; [3]Information Management Clinical Systems, Adaptimmune, Philadelphia, PA, USA; [4]Assay Development Histopathology, CellCarta, Antwerpen, Belgium; [5]Histopathology & Image Quantification Unit, CellCarta, Antwerpen, Belgium; [6]Translational Sciences, Adaptimmune, Abingdon, Oxfordshire, UK; [7]Target Validation, Adaptimmune, Abingdon, Oxfordshire, UK; [8]Preclinical Research, Adaptimmune, Abingdon, Oxfordshire, UK; [9]Histocompatibility Laboratory Services, American Red Cross, Philadelphia, PA, USA; [10]Clinical Science, Adaptimmune, Philadelphia, PA, USA; [11]Late Stage Development, Adaptimmune, Philadelphia, PA, USA; [12]Department of Medical Oncology and Hematology, Princess Margaret Cancer Centre, Departments of Immunology and Medicine, University of Toronto, Toronto, ON, Canada; [13]Department of Medicine, Duke Cancer Institute, Durham, NC, USA; [14]Department of Medicine, Massachusetts General Hospital, Boston, MA, USA; [15]Department of Medicine, Washington University School of Medicine, St. Louis, MO, USA; [16]Oncology, START Madrid FJD, Hospital Universitario Fundación Jiménez Díaz, Madrid, Spain; [17]Lung Cancer Research and Drug Development, Sarah Cannon Research Institute, Nashville, TN, USA; [18]Department of General Oncology, The University of Texas MD Anderson Cancer Center, Houston, TX, USA; [19]Department of Investigational Cancer Therapeutics, The University of Texas MD Anderson Cancer Center, Houston, TX, USA; [20]Thoracic/Head and Neck Medical Oncology, The University of Texas MD Anderson Cancer Center, Houston, TX, USA

T cell receptor (TCR) T cell therapies target tumor antigens in a human leukocyte antigen (HLA)-restricted manner. Biomarker-defined therapies require validation of assays suitable for determination of patient eligibility. For clinical trials evaluating TCR T cell therapies targeting melanoma-associated antigen A4 (MAGE-A4), screening in studies NCT02636855 and NCT04044768 assesses patient eligibility based on: (1) high-resolution HLA typing and (2) tumor MAGE-A4 testing via an immunohistochemical assay in HLA-eligible patients. The HLA/MAGE-A4 assays validation, biomarker data, and their relationship to covariates (demographics, cancer type, histopathology, tissue location) are reported here. HLA-A*02 eligibility was 44.8% (2,959/6,606) in patients from 43 sites across North America and Europe. While HLA-A*02:01 was the most frequent HLA-A*02 allele, others (A*02:02, A*02:03, A*02:06) considerably increased HLA eligibility in Hispanic, Black, and Asian populations. Overall, MAGE-A4 prevalence based on clinical trial enrollment was 26% (447/1,750) across 10 solid tumor types, and was highest in synovial sarcoma (70%) and lowest in gastric cancer (9%). The covariates were generally not associated with MAGE-A4 expression, except for patient age in ovarian cancer and histology in non-small cell lung cancer. This report shows the eligibility rate from biomarker screening for TCR T cell therapies and provides epidemiological data for future clinical development of MAGE-A4-targeted therapies.

INTRODUCTION

Adoptive cell therapies (ACTs) have improved patient outcomes in various therapeutic settings by employing activated lymphocytes to elicit anti-tumor effects[1–5]; however, ACT success largely depends on tumor characteristics. For metastatic solid tumors, T cell receptor (TCR) T cell therapies may overcome limitations of other ACTs, such as narrow applicability and/or decreased potential to activate the immune response.[6] TCR T cell therapies are genetically modified to

Received 29 September 2023; accepted 10 May 2024;
https://doi.org/10.1016/j.omtm.2024.101265.

[21]These authors contributed equally

[22]These authors contributed equally

Correspondence: Tianjiao Wang, Clinical Biomarkers & Companion Diagnostics, Adaptimmune, Philadelphia, PA, USA.
E-mail: Tianjiao.Wang@adaptimmune.com
Correspondence: Jean-Marc Navenot, Clinical Biomarkers & Companion Diagnostics, Adaptimmune, Philadelphia, PA, USA.
E-mail: Jean-Marc.Navenot@adaptimmune.com
Correspondence: David S. Hong, Department of Investigational Cancer Therapeutics, The University of Texas MD Anderson Cancer Center, Houston, TX, USA.
E-mail: dshong@mdanderson.org
Correspondence: George R. Blumenschein, Thoracic/Head and Neck Medical Oncology, The University of Texas MD Anderson Cancer Center, Houston, TX, USA.
E-mail: gblumens@mdanderson.org

This is an open access article under the CC BY license (http://creativecommons.org/licenses/by/4.0/).

Table 1. Afami-cel reactivity to MAGE-A4 peptides presented by different HLA-A2 subtypes, as determined *in vitro* using an IFN-γ cell-ELISA method following challenge of afami-cel with exogenous MAGE-A4 peptide in the context of MAGE-A4-negative tumor lines transduced with HLA-A2 subtypes

Transduced HLA-A*02 allele	Average log(EC_{50}) M
HLA-A*02:01	−7.8
HLA-A*02:02	−8.1
HLA-A*02:03	−7.4
HLA-A*02:05	−8.8
HLA-A*02:06	−8.3
HLA-A*02:07	−6.5

EC_{50}, half-maximal effective concentration; ELISA, enzyme-linked immunoassay; HLA, human leukocyte antigen; MAGE-A4, melanoma-associated antigen A4.

target specific, internally derived peptides presented on tumor cell surfaces by human leukocyte antigen (HLA) molecules. For a particular TCR T cell therapy to function, a person must express the appropriate HLA type complexed with the tumor peptide that the TCR was engineered to target. Screening is therefore required to identify individuals most likely to benefit from any given product in this therapeutic modality.

To maximize the number of individuals eligible for TCR T cell therapy, the targeted peptide-HLA complex must be carefully selected. High genetic variability exists in the alleles encoding distinct HLA molecules; however, structural and functional homologies within alleles from the same allele group may allow presentation of the same antigenic peptide by multiple different alleles.[7] HLA molecules have structural requirements for the peptides they are capable of presenting. Therefore, peptides derived from any given cancer-associated protein are typically only able to bind with sufficient affinity to a limited number of HLA alleles. Engineered TCRs are often designed to recognize tumor peptides complexed with HLA-A*02 alleles because they are observed across many populations,[8] thereby increasing the likelihood of patient eligibility. While A*02:01 is the most common HLA-A*02 subtype in most populations,[9,10] other HLA-A*02 subtypes represent significant proportions in some populations. Optimal characteristics of antigenic peptides for TCR T cell therapy include immunogenicity, cancer specificity, and expression across tumor types.[11] Melanoma-associated antigen A4 (MAGE-A4) is a cancer testis antigen absent in most healthy tissues but differentially expressed in several solid tumors, including synovial sarcoma (SyS), lung, bladder, head and neck, ovarian, and esophageal cancers.[12–14]

Afamitresgene autoleucel (afami-cel, formerly ADP-A2M4) and its next-generation counterpart, uzatresgene autoleucel (uza-cel, formerly ADP-A2M4CD8), are TCR T cell therapies engineered to target MAGE-A4 in HLA-A*02-eligible patients. Afami-cel and uza-cel express the same high-affinity MAGE-A4-targeted TCR, whereas uza-cel includes expression of an additional CD8α coreceptor for enhanced CD4+ T cell functionality and increased cytotoxic potency overall. Both have shown responses across multiple different cancer types.[15,16]

Here, we describe the preclinical characterization of the HLA-A*02 alleles that are functionally able to bind the target MAGE-A4-derived peptide and activate the TCR, and are therefore defined as inclusion alleles for patient eligibility. Accurate and robust assays for HLA typing (high resolution) and MAGE-A4 expression are needed to screen and enroll patients into clinical trials of TCR T cell therapies including afami-cel and uza-cel. We present the validation of the HLA and MAGE-A4 assays suitable for identification of eligible patients, as well as data from a multinational screening study (NCT02636855) that prospectively evaluated HLA subtypes and MAGE-A4 profiles to determine eligibility to enroll in clinical trials assessing the safety and efficacy of TCR T cell therapy in patients with metastatic solid cancers and from the SPEARHEAD-1 registrational study (NCT04044768) of afami-cel in SyS and myxoid/round cell liposarcoma (MRCLS).

RESULTS

Afami-cel selectivity to different HLA subtypes

Afami-cel displayed comparable *in vitro* potency toward peptide presented by HLA-A*02:01, 02:02, 02:03, and 02:06, while the response to peptide in the context of A*02:07 was >10-fold less potent than A*02:01 (Table 1). Similar interferon-γ (IFN-γ) responses were observed from afami-cel toward MAGE-A4–positive (MAGE-A4+) tumor lines natively expressing A*02:01, 02:02, 02:03, and 02:06, but no response was observed toward the same lines when expressing A*02:07 (Figure S1) in the absence of added exogenous peptide. Based on the above functional study, HLA-A*02:01, A*02:02, A*02:03, and A*02:06 were defined as inclusion alleles. Although similar or greater responses were observed toward target lines expressing HLA-A*02:05 compared with HLA-A*02:01, previous study has identified alloreactivity toward this allele.[17] Therefore, HLA-A*02:05 was defined as an exclusion allele. Alleles sharing the same protein sequence in domains α1 and α2 (P group) are functionally identical and are also considered inclusion or exclusion alleles. For HLA eligibility in clinical trials investigating afami-cel and uza-cel, a patient should have at least one inclusion HLA-A*02 allele and no exclusion allele (A*02:05P). The ability of the HLA typing assay selected to perform the necessary high-resolution (two-field) typing to differentiate these relevant alleles was demonstrated in an accuracy study.

Accuracy of the SeCore assay for HLA typing

When using the SeCore assay, all 70 samples yielded typing results with the SeCore assay that were consistent with the reference genotype, either from the published database or established by the AllType next-generation sequencing (NGS) assay. Among these 70 samples, 27 yielded results without ambiguities (i.e., only one possible genotype), whereas 43 gave ambiguous results (i.e., two or more possible genotypes) requiring group-specific sequencing primers (GSSP) sequencing. GSSP sequencing completely resolved 40 of these ambiguities (i.e., only one possible genotype remained). Two of the three remaining samples with ambiguities included null alleles

Table 2. HLA-A typing results by race and ethnicity

	Overall	
Race/ethnicity	Screened, *N* (%)	Eligible, *n* (%)
White, not Hispanic or Latino	5,249 (79.5)	2,481 (47.3)
White, Hispanic or Latino	260 (3.9)	112 (43.1)
White, not specified	58 (0.9)	31 (53.4)
Black or African American	319 (4.8)	85 (26.6)
Asian	435 (6.6)	124 (28.5)
American Indian or Alaska Native	22 (0.3)	8 (36.4)
Native Hawaiian or Pacific Islander	14 (0.2)	1 (7.1)
Not recorded	30 (0.5)	15 (50.0)
Other	219 (3.3)	102 (46.6)
Total	6,606 (100.00)	2,959 (44.8)

HLA, human leukocyte antigen.

resulting from insertion or deletion in the coding sequence, and the uTYPE software required, by design, the analyst to confirm the sequence to resolve the ambiguity. For the last sample, GSSP sequencing reduced the list of ambiguities to only two possible genotypes, including the reference genotype. The alternative allele combination (A*02:135/69:02) could not be excluded by GSSP but was flagged by the uTYPE software as a combination of two rare alleles. With all samples being concordant, the lower limit of the one-sided 95% CI of concordance between the reference and the SeCore genotypes exceeded 95% (the threshold established in the FDA guidance for industry, 2015) with both the Clopper-Pearson exact test (95.81%) and the Wilson exact test (96.28%).

HLA typing in the screening study and SPEARHEAD-1

A total of 6,606 patients from 43 sites in the US (30), Canada (1), Spain (7), the UK (2), and France (3) had their HLA-A type determined (6,167 from the screening study and 439 from the SPEARHEAD-1 study); among them, 2,959 (44.8%) were eligible based on the criteria for receiving afami-cel or uza-cel. Patients who had both an inclusion allele and an A*02:05P allele were ineligible (n = 29; 0.44% of those screened). In patients for whom demographic information was available, eligibility rate was different between races and ethnicitiesr (Table 2). While a higher percentage of White patients was eligible due to HLA-A*02:01P, adding A*02:02, 02:03, and 02:06 as inclusion alleles significantly increased HLA eligibility in some other populations, in particular A*02:06 in Hispanic and Latino patients, A*02:02 in African American patients, and both A*02:03 and A*02:06 in Asian patients (Figure 1). Among eligible participants, the percentages of patients eligible due to the expression of at least one of these three alleles (A*02:02, 02:03, and 02:06), without also expressing A*02:01P, were: 12.6% of Hispanic or Latino, 17.7% of Black or African American, and 55.6% of Asian patients.

Performance of the MAGE-A4 IHC clinical trial assay

The anti-MAGE-A4 antibody (clone OTI1F9) showed specific staining of MAGE-A4 without cross-reactivity to MAGE-A1, -A2, -A3, -A6, -A9 (in MAGE-A-transduced NALM6 cell lines), -A10 (in the Mel526 cell line with endogenous high MAGE-A10 expression), and -A11 and -A12 (in Mel624). Additional details associated with the MAGE-A4 immunohistochemical (IHC) clinical trial assay are in the supplemental information, Tables S1–S4, and Figures S2–S16. The anti-MAGE-A4 antibody (clone OTI1F9) showed rare staining (0.28%) to MAGE-A8-transduced NALM6 cell line (high MAGE-A8 expression in 56% of cells), and minor cross-reactivity to MAGE-A10-transduced NALM6 cell line, artificial systems with extremely high MAGE-A8 or MAGE-A10 expression. The rare staining by anti-MAGE-A4 (clone OTI1F9) of MAGE-A8 had negligible impact on the diagnostic accuracy of the MAGE-A4 IHC clinical trial assay. The minor cross-reactivity of anti-MAGE-A4 antibody (clone OTI1F9) did not change the MAGE-A4 diagnosis status in tumor tissues with high MAGE-A10 expression as determined by a MAGE-A10 IHC clinical trial assay used to screen patients for a MAGE-A10-targeting TCR T cell study (Figure S6E). In the analytical validation, the tumor/tissue samples showed different MAGE-A4 prevalence in a broad range of solid tumors but not in normal tissues (except testis and placenta). The precision of the MAGE-A4 IHC clinical trial assay was validated at the cutoff (≥30% at ≥2+ intensity) with ≥80% inter-run/intra-run concordance (mostly ≥90%). Inter-lab assay transfer showed 100% concordance on a series of samples of multiple indications. Pathologists' scoring showed ≥80% (mostly ≥90%) intra-/inter-reader concordance for a series of samples of multiple indications.

MAGE-A4 expression

In the 2,959 HLA-eligible patients, 1,750 had tumor samples evaluable for MAGE-A4 across 31 sites in the US (18), Canada (1), France (3), the UK (2), and Spain (7); among these, 447 patients were MAGE-A4+ (≥30% tumor cells stained at ≥2+ intensities). Representative IHC images, with specific staining of MAGE-A4 in the cytoplasm and nuclei of tumors cells with different staining intensities (0–3+), are shown in Figure 2, and expression in the different tumor types in Figure S17. Most tumor samples (93%) were collected within 4 years of testing, with 55% of them collected within 1 year of testing, and overall archival time ranging from 0 to 20 years. Overall, MAGE-A4+ rate varied among individual tumor types (Figure S18A) but remained similar within 5 years of tissue archival (Figure S18B). Lower MAGE-A4 prevalence observed in samples collected >5 years before testing in some tumor types may reflect the small sample size of tissue archived >5 years before testing, differences in tumor biology, or compromised MAGE-A4 stability (Figures S18A and S18B). However, no relationship between MAGE-A4 protein score (P score) and tissue archival time could be demonstrated; some samples archived for up to 9 years still had a P score of 97, indicating stability of MAGE-A4 in formalin-fixed, paraffin-embedded (FFPE) archived tissues and suitability of older tissue blocks for eligibility purposes (Figure S18C).

The MAGE-A4+ rate was highest in SyS (70%, 140/201) and lowest in gastric cancer (9%, 6/70), but was seen across all tumor

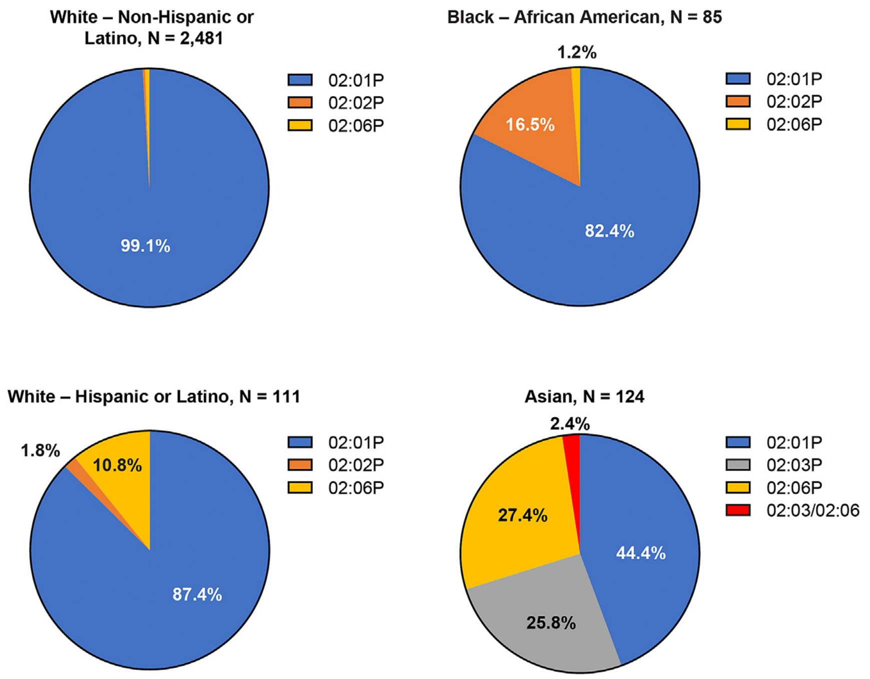

Figure 1. Relative contribution of HLA-A*02 inclusion alleles to HLA eligibility for afamitresgene autoleucel by race and ethnicity

Overall, most HLA-eligible participants were eligible based on the expression of A*02:01 or one of its P-group members (in blue, e.g., 02:09 or 02:642), either as the only inclusion allele (heterozygous or homozygous), or combined with another inclusion allele. However, some participants were eligible based only on the expression of other inclusion alleles: A*02:02 (orange), 02:03 (gray), 02:06 (yellow), or both 02:03 and 02:06 (red). The percentage of eligible participants exclusively expressing these inclusion alleles varied greatly by race and ethnicity. HLA, human leukocyte antigen.

types investigated, including MRCLS (40%, 27/67), urothelial cancer (32%, 30/93), esophagogastric junction (EGJ) cancer (26%, 24/93), ovarian cancer (24%, 54/226), head and neck cancer (22%, 43/200), esophageal cancer (21%, 21/100), melanoma (16%, 39/243), and NSCLC (14%, 63/457) (Figure S17). MAGE-A4 expression level was highest, on average, in SyS (median P score = 76), followed by MRCLS (median P score = 15), urothelial cancer (median P score = 5), ovarian cancer (median P score = 2), and the rest of the cancer types (median P score = 0) (Figure S17B), with some samples reaching a P score of 100 in all indications other than MRCLS (highest P score = 94). In a non-pairwise analysis of all tested samples, MAGE-A4 was detected at relatively similar frequencies in primary and metastatic tumors, although higher MAGE-A4+ rate was observed in metastatic tumors of urothelial cancer and melanoma while lower MAGE-A4+ rate was observed in metastatic tumors of head and neck cancer and gastric cancer (Figure 3). In pairwise analysis of MAGE-A4 expression in both primary and metastatic tumor tissues, 16 patients had both samples and 81% (13/16) of these patients had the same MAGE-A4 diagnosis status (positive or negative) regardless of tissue location (primary or metastatic) (data not shown). In both esophageal cancer and NSCLC, MAGE-A4+ rate and expression level were higher in squamous cell carcinoma (SCC) samples, compared with adenocarcinoma (AC) samples (Figure 4). MAGE-A4+ rate was not correlated with the age of patients in esophageal cancer, EGJ cancer, head and neck cancer, NSCLC, or SyS. An apparent negative correlation of MAGE-A4 positivity with patient age was observed in gastric cancer and MRCLS, while an apparent positive correlation was observed in melanoma, ovarian cancer, and urothelial cancer (Figure 5).

As per the univariate and multivariate analyses within each cancer type, the covariates were generally not associated with MAGE-A4 expression, except for patient age in ovarian cancer and histology in NSCLC. After adjusting for confounding factors, patient age (odds ratio [OR] = 9.86; 95% CI, 2.87–62.12) was positively associated with the MAGE-A4 expression in ovarian cancer. In addition, MAGE-A4 expression in NSCLC was significantly higher (OR = 10.02; 94% CI, 5.36–19.54) in samples of SCC compared with AC.

DISCUSSION

Identifying individuals who are most likely to benefit from treatment is a requirement for precision medicine therapeutic products. Based on the mechanism of action of TCR T cell therapy, screening for HLA genotype and tumor antigen expression are the two components of the biomarker-driven identification of eligible patients. Based on the HLA and MAGE-A4 prevalence for clinical trial enrollment as reported in this study, eligibility (HLA eligible/MAGE-A4+) may vary from 4% to 31% depending on indications, highlighting the need for, and importance of, implementing a biomarker screening program that is both reliable and easily accessible for a TCR T cell therapy. Results from the accuracy evaluations of the HLA typing assay and MAGE-A4 IHC clinical trial assay used in the screening study reported here indicate that they are reliable when assessing eligibility for clinical trials investigating afami-cel and uza-cel.

The process of designing a targeted TCR is founded in the identification of a tumor antigen that binds with sufficient affinity to particular HLA molecules. Because alleles in the HLA-A*02 group are most frequently expressed in many populations around the world, they are often preferred when identifying appropriate tumor antigens.

Eligibility rates based on HLA criteria in the screening protocol and the SPEARHEAD-1 study were consistent with expectations based on public databases on HLA-A*02 allele frequencies. Data from the US National Marrow Donor Program[18,19] show that A*02:01P is

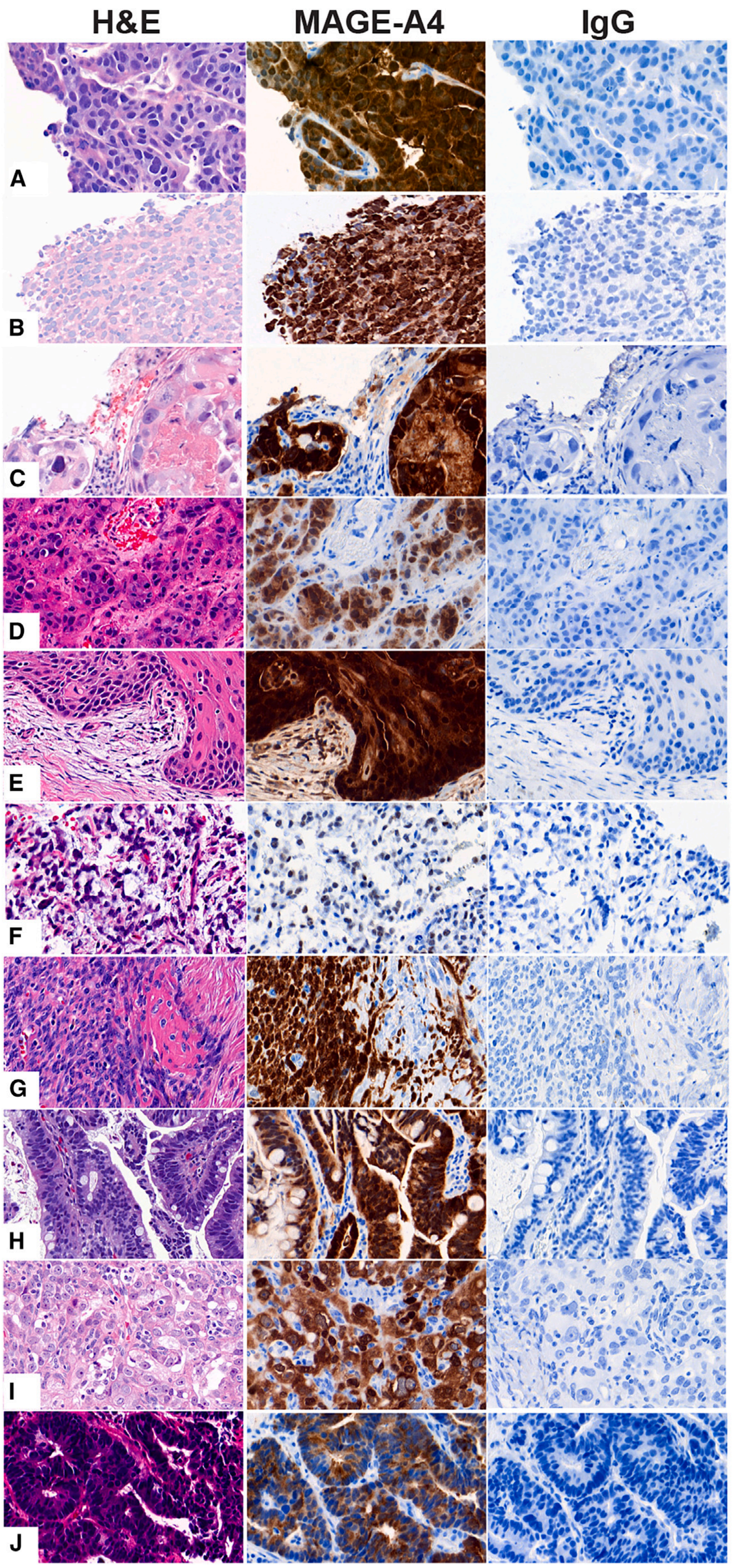

Figure 2. Representative images of histological and MAGE-A4 staining in different cancers screened

Tissues from patients with esophagogastric junction (A), melanoma (B), non-small cell lung sarcoma (C), urothelial (D), head and neck (E), myxoid/round cell liposarcoma (F), synovial sarcoma (G), esophageal (H), ovarian (I), or gastric (J) cancers. The cancer tissue was stained with H&E, anti-MAGE-A4 antibody (MAGE-A4), and isotype control (IgG), visualized in the left, middle, and right columns, respectively. MAGE-A4 immunoreactivity demonstrated specific expression in each cancer tissue. Magnification of the images: 40×. H&E, hematoxylin and eosin; IgG, immunoglobulin G; MAGE-A4, melanoma-associated antigen A4.

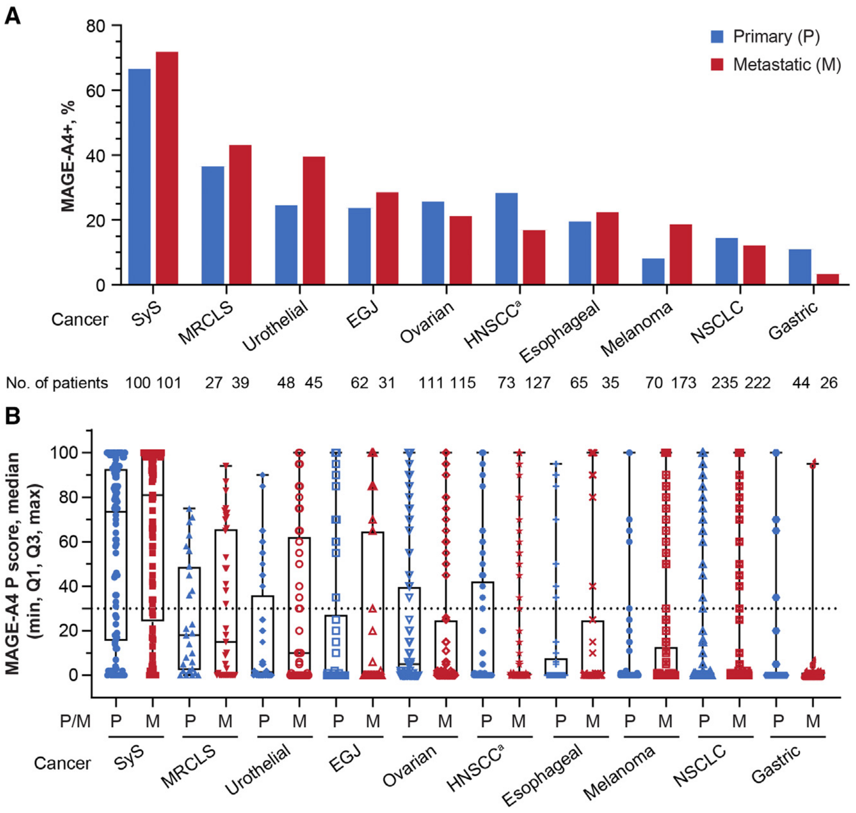

Figure 3. MAGE-A4 positivity and expression level by tissue location (primary or metastatic) across cancer types

(A) MAGE-A4 positivity. (B) MAGE-A4 expression level. [a]n = 199 HNSCC, n = 1 "other" head and neck cancer histology. The dotted line represents the cutoff value of the P score indicating MAGE-A4 positivity. EGJ, esophagogastric junction cancer; HNSCC, head and neck squamous cell carcinoma; M, metastatic; MAGE-A4, melanoma-associated antigen A4; MRCLS, myxoid/round cell liposarcoma; NSCLC, non-small cell lung cancer; P, primary; P score, protein score; SyS, synovial sarcoma.

generally the most frequent allele, but its frequency is higher in White populations (47.5% of individuals) and lower in Asian (18%) and Black or African American (23%) populations (Table S5). In our study, inclusion of A*02:02P, A*02:03P, and A*02:06P increased the proportion of eligible patients across Asian, Hispanic or Latino, and Black or African American populations. A higher percentage of White patients than other races and ethnicities are eligible to receive an A*02:01-restricted immunotherapy such as afami-cel. In this report, we show that the validations of alleles other than A*02:01 (A*02:02, A*02:03, and A*02:06) as inclusion criteria for studies offset genetic bias to some extent and increase eligibility in other populations. Whereas public databases report allele frequencies in specific populations, they do not indicate how specific alleles co-segregate within subgroups of a given race or ethnicity, which can lead to over- or underestimation of the percentage of population positive for a set of alleles from the same locus. Our genotyping data provide that insight.

Our results indicate that MAGE-A4 expression can be reliably assessed in fresh biopsy or archival tissues (up to 5 years old); however, the effect of storage time of FFPE blocks on MAGE-A4 positivity beyond 5 years would need further investigation in a longitudinal study. Consistent with previous reports (Table S6 and references thereof),[20] MAGE-A4 expression in this study was found at varying levels across tumor types. The difference of MAGE-A4 prevalence in this study in comparison with literature reports may be due to differences in assays used and their positivity cutoff, as well as patient populations and disease clinicopathology. The clinical utility of HLA/MAGE-A4 as

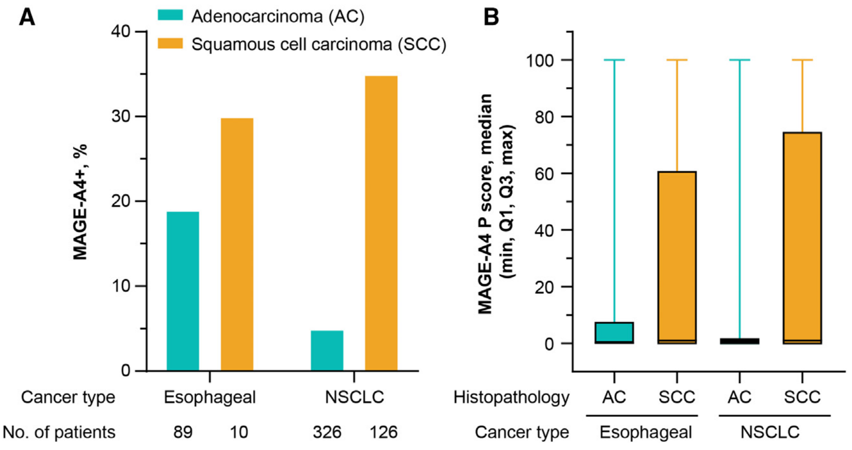

Figure 4. MAGE-A4 positivity and expression level by histopathology

(A) MAGE-A4 positivity. (B) MAGE-A4 expression level. AC, adenocarcinoma; MAGE-A4, melanoma-associated antigen A4; MAGE-A4+, MAGE-A4 positive; NSCLC, non-small cell lung cancer; P score, protein score; SCC, squamous cell carcinoma.

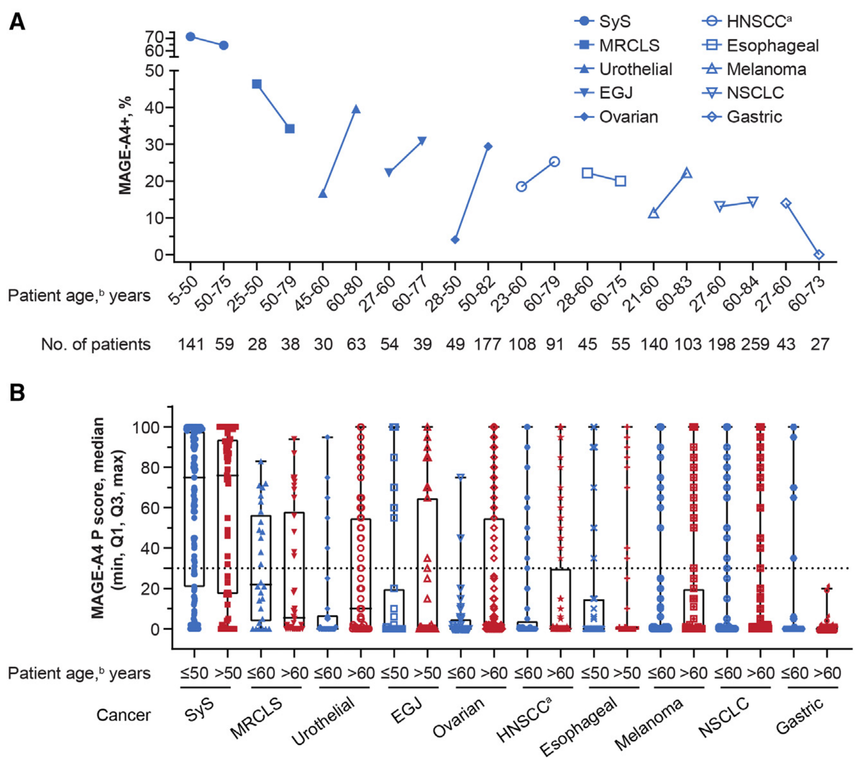

Figure 5. Effect of patient age on MAGE-A4 positivity and expression level across cancer types

(A) MAGE-A4 positivity. (B) MAGE-A4 expression level. [a]n = 199 HNSCC, n = 1 "other" head and neck cancer histology. [b]Patient age was determined at biopsy collection. The dotted line represents the cutoff value of the P score indicating MAGE-A4 positivity. EGJ, esophagogastric junction cancer; HNSCC, head and neck squamous cell carcinoma; MAGE-A4, melanoma-associated antigen A4; MAGE-A4+, MAGE-A4 positive; MRCLS, myxoid/round cell liposarcoma; NSCLC, non-small cell lung cancer; P score, protein score; SyS, synovial sarcoma.

biomarkers in selecting patients for MAGE-A4-targeted TCR T cell therapies has been demonstrated in two phase 1 trials in multiple indications, including SyS, ovarian cancer, urothelial cancer, and head and neck SCC.[15,16] Their clinical utility has been further confirmed in a phase 2 trial of afami-cel in SyS and MRCLS.[21] Thus, the MAGE-A4 prevalence reported here is of significance in guiding future clinical trial development for anti-MAGE-A4 TCR T cell therapies. In our samples, SyS showed the highest MAGE-A4 expression, whereas gastric cancer showed the lowest. Among the potential factors affecting MAGE-A4 expression, we found no consistent correlation between patient age and MAGE-A4 expression; however, older age of patients was associated with higher MAGE-A4 expression in ovarian cancer, consistent with previous reports.[22] In addition, MAGE-A4 expression in relation to tumor location (primary vs. metastatic lesions) was generally comparable across the cancer types included in this study, although higher MAGE-A4 expression in metastatic melanoma lesions was noted, in line with what was shown previously.[23] This implies that a cancer tissue, regardless of its origin/tissue location, may be used to determine a patient's MAGE-A4 eligibility at screening, without significant impact on the screening efficiency. Finally, we found that SCC compared with AC had significantly higher MAGE-A4 expression in NSCLC, consistent with prior reports.[24] As patient age, tumor location (primary vs. metastatic), and histology type impact prognosis after cancer treatment, increased understanding of their relationships to MAGE-A4 expression may shed light on clinical development of MAGE-A4-targeted T cell therapy, including patient screening/selection and trial design.

There are some limitations of this study. First, MAGE-A4 prevalence and expression levels may be subjected to tumor heterogeneity, clinicopathology, and sample size; most of the patients in this screening study only had one tissue block submitted for MAGE-A4 testing and some tumor types had a limited samples size (e.g., MRCLS, n = 67; gastric cancer, n = 70). Second, factors that may affect the determination of MAGE-A4 expression, including tumor location (primary vs. metastatic) and archived tissue storage time, are neither pairwise (for tumor locations) nor longitudinal (for archived tissue storage time), and the impact of archived tissue storage time greater than 5 years (7% of the total samples tested) on MAGE-A4 expression may be uncertain. Third, the actual target of afami-cel and uza-cel is the complex of HLA-A*02 and the MAGE-A4-derived peptide; however, there is currently no technology able to detect that complex on the surface of tumor cells, especially in FFPE samples. The current screening tests (germline HLA typing and expression of MAGE-A4 by IHC in tumor samples) are admittedly a surrogate for the detection of the peptide-HLA complex. However, the overall response rate per RECIST 1.1 obtained in SyS and other indications demonstrates the clinical validity of the screening process.[15,16,21]

Taken together, HLA-A genotype and MAGE-A4 tumor expression are key biomarkers to assess patient eligibility to enroll in various trials of TCR T cell therapy, including those investigating the safety and efficacy of afami-cel and uza-cel. This is the first report of a large-scale HLA and MAGE-A4 prospective screening study with demonstrated clinical utility of the biomarkers, setting a foundation of biomarker screening for TCR T cell therapies, and illustrating the extent of screening required for therapies of this type. The findings on HLA prevalence in different races/ethnicities and MAGE-A4 expression in different tumor and histology types and the impact of patient age in certain cancers (e.g., ovarian cancer) may guide future clinical development of TCR T cell therapies, including disease selection strategy, patient eligibility criteria, trial design, and investigation into different HLA-restricted TCRs. The impact of tumor location (primary vs. metastatic) and archived tissue storage time on antigen

determination also provides practical guidance on sample collection for screening and eligibility determination. Future efforts are warranted to further address assay implementation issues and to develop accurate and robust single-plex or multiplex screening assays that are easily deployable and accessible to meet the functional needs of TCR T cell therapies.

MATERIALS AND METHODS

Functional assessment of afami-cel selectivity to different HLA subtypes

To explore the functional response of MAGE-A4-targeted TCR T cell therapies to common HLA-A*02 subtypes, MAGE-A4+ HLA-A*02-negative tumor cell lines were transduced using lentiviral vectors expressing HLA-A*02 alleles, green fluorescent protein, and puromycin-N-acetyltransferase. These lines were selected through culture in puromycin, and comparable levels of transgene expression were confirmed by flow cytometry (data not shown). The ability of these lines to induce an afami-cel response was subsequently assessed by IFN-γ cell enzyme-linked immunoassay (ELISA). Generation of afami-cel TCR T cells was described previously.[17] For the cell-based ELISA, 384-well plates were coated with IFN-γ capture antibodies overnight followed by plating of target cells (10^4/well), effector T cells (10^4/well) and/or peptide, or IFN-γ standards. After 48 h, the plates were washed and the assay was carried out following the manufacturer's protocol (Human IFN-gamma DuoSet ELISA, R&D Systems, Minneapolis, MN), with the use of a luminescent HRP substrate (Glo Substrate, R&D Systems). Luminescence was measured using a FLUOstar Omega plate reader (BMGLabtech, Cary, NC). Peptide response curve fitting was performed using the drc R package using a three-parameter log-logistic function.[25]

Protocol design

The screening and SPEARHEAD-1 studies adhered to the principles outlined in the Declaration of Helsinki and were conducted according to the International Council for Harmonisation's Guideline for Good Clinical Practice. Written informed consent was obtained from all patients prior to any study-related procedures being performed. Design of SPEARHEAD-1 has been reported previously.[21]

Men and women aged ≥18 to ≤75 years with advanced solid or hematologic malignancy and a life expectancy >3 months could enroll in the screening study. Eligible cancer types included melanoma, NSCLC, head and neck, gastric, EGJ, esophageal, ovarian, urothelial, SyS, and MRCLS cancers. Patients must have been able to provide blood samples and tumor samples (e.g., archived FFPE tumor blocks or tissue sections, or fresh biopsies if feasible).

Patients' sex, race, and ethnicity were determined based on self-reporting by checking boxes associated with their demographics. For biological sex, there were two options (i.e., male or female). The options for race and ethnicity were as follows: White, Black or African American, Asian, American Indian or Alaska Native, Native Hawaiian or Pacific Islander, Hispanic or Latino, not Hispanic or Latino, or other.

The primary endpoints of the screening protocol were determination of MAGE-A4 antigen expression profile and HLA genotype for subsequent assessment of their eligibility for clinical trials of afami-cel and uza-cel TCR T cell therapies. The exploratory endpoint was determination of incidence of antigen expression in different cancer types.

HLA typing

Blood samples were collected from screened patients as the source of DNA for HLA-A typing. High-resolution (two-field) typing of HLA-A was required to discriminate inclusion, exclusion, and neutral A*02 alleles and determine eligibility. All samples were typed via the SeCore assay (One Lambda, Thermo Fisher Scientific, Los Angeles, CA), a Sanger sequencing-based typing assay that has received 510(k) approval from the FDA. In brief, amplification by polymerase chain reaction of HLA-A alleles using locus-specific primers was followed by bi-directional sequencing of exons 1 to 5 on an ABI 3730xl DNA Analyzer (Thermo Fisher Scientific) and subsequent analysis using uTYPE HLA Sequence Analysis Software (Thermo Fisher Scientific). When necessary, ambiguities were resolved using the SeCore GSSP or an SSP assay if an appropriate GSSP was not readily available. Buccal swabs were used for HLA typing of patients who consented remotely (n = 58); those who were determined to be HLA eligible and whose tumor expressed MAGE-A4 had their HLA type confirmed with a blood sample (n = 4). All testing occurred at Histocompatibility Laboratory Services, American Red Cross, in Philadelphia, and IMGM, in Martinsried, Germany.

An accuracy study using both well-characterized samples expressing several frequent and less frequent A*02 alleles and DNA samples from patients with SyS was conducted to assess the capacity of the SeCore assay to correctly assign genotype and its suitability for the intended use. The AllType NGS assay (One Lambda) was used as the predicate for samples that were not well characterized or for which the published genotype was erroneous. Seventy DNA samples were evaluated including 64 from Epstein-Barr virus-transformed lymphoblastic cell lines, which were procured from the International Histocompatibility Working Group (n = 34) or from the Class I UCLA DNA Reference Panel (n = 28), or were derived in-house (n = 2). In addition, six samples were from patients with SyS who were screened for eligibility to participate in a TCR T cell therapy clinical trial. Concordance between the SeCore genotype and the reference genotype (published or established with the AllType assay) was defined as the reference genotype being either identical to the SeCore genotype (absence of ambiguities) or being included among the possible SeCore genotypes (presence of ambiguities).

MAGE-A4 expression

MAGE-A4 testing of tumor samples, either an archived FFPE specimen or a fresh biopsy, in HLA-eligible patients was carried out via an IHC clinical trial assay. Tissue blocks were cut as 4-μm thickness slides, pretreated in the 3 in 1 PT module with TRS low pH antigen retrieval solution (Dako-K8005) at 60°C for 2 h and then at 97°C for 20 min, and then stained on the Dako autostainer Link 48

platform with an anti-MAGE-A4 monoclonal antibody (clone OTIF9, Origene, TA505362, 10 μg/mL) and an IgG isotype control antibody (IgG2a, Sigma Aldrich, M9144, 10 μg/mL) for 30 min at room temperature (22°C–25°C), visualized by the EnVision+ System-HRP Labelled Polymer (Dako-K4001) combined with a Dako Liquid DAB Substrate Chromogen System (Dako-K3468) for 30 and 5 min, respectively, counterstained with hematoxylin for 5 min, and then finally cover-slipped on the Sakura coverslipper, which are all qualified and validated at CellCarta. The MAGE-A4 IHC clinical trial assay was performed at a Clinical Laboratory Improvement Amendments 1998-certified and College of American Pathologists-accredited central laboratory, and specificity, precision, inter-lab concordance, and pathologist scoring concordance were analyzed to evaluate assay performance (details in supplemental information).

MAGE-A4 expression was determined by both percentage of tumor cell staining and intensity of cell staining (nuclear/cytoplasmic staining at 0, 1+, 2+, 3+ intensity). MAGE-A4 expression level was defined by P score (percent of tumor cells staining at 2+, 3+). P score was initially defined as tumor samples with percent cell staining at ≥1+ with 10% cutoff for screening/enrollment. A protocol amendment shifted this cutoff to a P score ≥30% at ≥2+ for MAGE-A4 positivity, which is used in all clinical trials. Both the MAGE-A4+ rate (%) and MAGE-A4 expression level (median P score) are reported here, based on the cutoff of P score ≥30% at ≥2+. H score is assessed as part of our translational research but is not used to determine eligibility, therefore it is not included with the screening protocol data.

Statistical analyses

The biomarker (HLA and MAGE-A4) screening samples were collected between May 22, 2017 and November 19, 2021 for this analysis for the screening study (NCT02636855) and from June 26, 2019 until October 22, 2021 for SPEARHEAD-1 (NCT04044768). Covariates of MAGE-A4 expression used in this study were demographics (sex, age), histopathology (cancer type, tumor subtype [primary vs. metastatic], estimated number of cancer cells, and estimated percentage of inflammatory cells), histology type (AC and SCC), and FFPE sample storage time. Covariates were assessed by univariate and multivariate methods using logistic regression modeling, and results are presented as odds ratios with 95% CIs and *p* values. Age, estimated number of cancer cells, FFPE sample storage time, sex, tumor subtype, and histology were categorical variables, whereas estimated percentage of inflammatory cells was a continuous variable. Software tools used for this study are available as open-source R v.3.6.3 and associated packages.

DATA AND CODE AVAILABILITY

All data relevant to the study are included in the article or uploaded as supplemental information. The raw datasets generated, used, and analyzed during the current study are available from the corresponding author on reasonable request.

SUPPLEMENTAL INFORMATION

Supplemental information can be found online at https://doi.org/10.1016/j.omtm.2024.101265.

ACKNOWLEDGMENTS

This study was sponsored by Adaptimmune. Writing support for this manuscript was provided by Gabrielle Knafler, PhD, of Envision Pharma Inc. (Fairfield, CT), which was contracted and compensated by Adaptimmune for these services. We would like to acknowledge and thank Robert Connacher for his preparation of Figure 2, and Adriano Quattrini for manufacturing the engineered T cells. We would also like to thank the study participants. This study was approved by the ethics committees of the participating screening sites.

AUTHOR CONTRIBUTIONS

T.W. and J.-M.N. were responsible for the concept design. T.W., J.-M.N., S.R., C.K., M.V.K., S.V.R., K.S., K.A., R.B., K.H., A.L., A.C., L.Q., J.P.S., C.M., C.C., E.P., and S.H. developed the initial proof-of-concept studies. T.W., J.-M.N., S.R., C.K., M.V.K., S.V.R., K.S., K.A., C.C., E.P., S.H., F.B., E.E., P.B., D.W., G.B.J., M.O.B., J.M.C., J.F.G., R.G., V.M., M.J., J.T., and D.S.H. oversaw the project. T.W., J.-M.N., S.R., C.K., M.C., M.V.K., S.V.R., K.S., K.A., L.Q., J.P.S., C.M., C.C., E.P., S.H., F.B., E.E., and P.B. were responsible for the experimental design, execution of assays, and data acquisition. T.W., J.-M.N., S.R., R.W., M.V.K., S.V.R., K.S., K.A., and J.P.S. analyzed and interpreted the data. All authors reviewed the manuscript and approved the final version for publication.

DECLARATION OF INTERESTS

T.W., J.-M.N., S.R., C.K., M.C., R.B., K.H., A.L., A.C., R.W., L.Q., J.P.S., C.M., F.B., E.E., P.B., and D.W. are or were employees of Adaptimmune at the time of the study and may own stock/stock options in Adaptimmune.

M.V.K., S.V.R., K.S., and K.A. are employees of CellCarta NV, Antwerp, Belgium, and were involved in the development and validation of the MAGE-A4 IHC assay.

G.B.J. received consulting fees from AbbVie, Daiichi Sankyo, Eli Lilly, Genzyme, Gilead, Merck Sharp & Dohme, Novartis, Regeneron; expenses from Regeneron; research funding from AstraZeneca, BeiGene, Bristol Myers Squibb, Daiichi Sankyo, Exelixis, Genentech, Incyte, Merck Sharp & Dohme, Novartis, Regeneron.

M.O.B. received grant support from Merck, Takara Bio; quality improvement support from Novartis; is on the advisory board of Adaptimmune, Bristol Myers Squibb, GlaxoSmithKline, IDEAYA, Instil Bio, Iovance, La Roche Possey, Medison, Merck, Novartis, Pfizer, Regeneron, Sanofi, Sun Pharma; oral presentations given for Bristol Myers Squibb, Merck, Novartis, Pfizer, Sanofi.

J.M.C. received grant/research support from AbbVie, Adaptimmune, Array, AstraZeneca, Bayer, Bristol Myers Squibb, CBMG, Genentech, GlaxoSmithKline, Grid Therapeutics, Medpacto, Moderna, Spectrum; consultant for Amgen, Corbus, G1 Therapeutics, Novartis, Omega, Sanofi, Turning Point, Vivacitas; speaker's bureau for AstraZeneca.

J.F.G. was compensated consultant or received honoraria from AI Proteins, AstraZeneca, Blueprint Medicines, Bristol Myers Squibb, Curie Therapeutics, Genentech/Roche, Gilead, iTeos, Jounce, Karyopharm, Loxo/Lilly, Merck, Merus Pharmacueticals, Mirati, Moderna, Novartis, Novocure, Nuvalent, Pfizer, Silverback Therapeutics, Takeda; research support from Genentech/Roche, Novartis, Takeda; institutional research support from Adaptimmune, Alexo, Array Biopharma, Bristol Myers Squibb, Blueprint Medicines, Jounce, Merck, Moderna, Novartis, Palleon, Tesaro; equity in AI Proteins; and has an immediate family member who is an employee with equity at Ironwood Pharmaceuticals.

V.M. received consulting fees from Affimed, AstraZeneca, Bayer, Bristol Myers Squibb, Janssen, Roche, Syneos; was principal investigator – institutional funding from AbbVie, AceaBio, Adaptimmune, ADC Therapeutics, Aduro, Agenus, Amcure, Amgen, Astellas, AstraZeneca, Bayer, BeiGene, BioInvent International AB, Bristol Myers Squibb, Boehringer, Boston, Celgene, Daichii Sankyo, DEBIOPHARM, Eisai, e-Terapeutics, Exelisis, Forma Therapeutics, Genmab, GlaxoSmithKline, Harpoon, Hutchison, Immutep, Incyte, Inovio, Iovance, Janssen, Kyowa Kirin, Lilly, Loxo, MedSir, Menarini, Merck, Merus, Millennium, MSD, Nanobiotix, Nektar, Novartis, Odonate Therapeutics, Pfizer, Pharma Mar, PharmaMar, Principia, PsiOxus, Puma, Regeneron, Rigontec, Roche, Sanofi, Sierra Oncology, Synthon, Taiho, Takeda, Tesaro, Transgene, Turning Point Therapeutics, Upshersmith.

M.J. received research funding (institutional) from AbbVie, Acerta, Adaptimmune, Amgen, Apexigen, Arcus Biosciences, Array BioPharma, Artios Pharma, AstraZeneca, Atreca, BeiGene, BerGenBio, BioAtla, Black Diamond, Boehringer Ingelheim, Bristol Myers Squibb, Calithera Biosciences, Carisma Therapeutics, Checkpoint Therapeutics, City of Hope National Medical Center, Corvus Pharmaceuticals, Curis, CytomX, Daiichi Sankyo, Dracen Pharmaceuticals, Dynavax, Eli Lilly, Elicio Therapeutics, EMD Serono, EQRx, Erasca, Exelixis, Fate Therapeutics, Genentech/Roche, Genmab, Genocea Biosciences, GlaxoSmithKline, Gritstone Oncology, Guardant Health, Harpoon, Helsinn Healthcare SA, Hengrui Therapeutics, Hutchison MediPharma, IDEAYA Biosciences, IGM Biosciences, Immunitas Therapeutics, Immunocore, Incyte, Janssen, Jounce Therapeutics, Kadmon Pharmaceuticals, Kartos Therapeutics, Loxo Oncology, Lycera, Memorial Sloan Kettering, Merck, Merus, Mirati Therapeutics, Mythic Therapeutics, NeoImmune Tech, Neovia Oncology, Novartis, Numab Therapeutics, Nuvalent, OncoMed Pharmaceuticals, Palleon Pharmaceuticals, Pfizer, PMV Pharmaceuticals, Rain Therapeutics, RasCal Therapeutics, Regeneron Pharmaceuticals, Relay Therapeutics, Revolution Medicines, Ribon Therapeutics, Rubius Therapeutics, Sanofi, Seven and Eight Biopharmaceuticals/Birdie Biopharmaceuticals, Shattuck Labs, Silicon Therapeutics, Stem CentRx, Syndax Pharmaceuticals, Takeda Pharmaceuticals, Tarveda, TCR2 Therapeutics, Tempest Therapeutics, Tizona Therapeutics, TMUNITY Therapeutics, Turning Point Therapeutics, University of Michigan, Vyriad, WindMIL Therapeutics, Y-mAbs Therapeutics; had a consulting/advisory role (institutional) for AbbVie, Amgen, Arcus Biosciences, Arrivent, Astellas, AstraZeneca, Black Diamond, Boehringer Ingelheim, Calithera Biosciences, Daiichi Sankyo, EcoR1, Genentech/Roche, Genmab, Genocea Biosciences, Gilead Sciences, GlaxoSmithKline, Gritstone Oncology, Ideaya Biosciences, Immunocore, iTeos, Janssen, Jazz Pharmaceuticals, Merck, Mirati Therapeutics, Molecular Axiom, Normunity, Novartis, Oncorus, Pyramid Biosciences, Regeneron Pharmaceuticals, Revolution Medicines, Sanofi-Aventis, SeaGen, Synthekine, Takeda Pharmaceuticals, Turning Point Therapeutics, VBL Therapeutics.

D.S.H. received travel, accommodations, expenses from AACR, ASCO, CLCC, Bayer, Genmab, SITC, Telperian; had consulting, speaker, or advisory role with 28Bio, AbbVie, Acuta, Adaptimmune, Alkermes, Alpha Insights, Amgen, Affini-T, Astellas, Aumbiosciences, Axiom, Baxter, Bayer, Boxer Capital, BridgeBio, CARSgen, CLCC, COG, COR2ed, Cowen, EcoR1, Erasca, Fate Therapeutics, F. Hoffmann-La Roche, Genentech, Gennao Bio, Gilead, GLG, Group H, Guidepoint, HCW Precision Oncology, Immunogenesis, InduPro, Janssen, Liberium, MedaCorp, Medscape, Numab, Oncologia Brasil, ORI Capital, Pfizer, Pharma Intelligence, POET Congress, Prime Oncology, Projects in Knowledge, Quanta, RAIN, Ridgeline, SeaGen, Stanford, STCube, Takeda, Tavistock, Trieza Therapeutics, Turning Point Therapeutics, WebMD, YingLing Pharma, Ziopharm; has other ownership interests in Molecular Match (advisor), OncoResponse (founder, advisor), Telperian (founder, advisor); received institutional research/grant funding from AbbVie, Adaptimmune, Adlai-Nortye, Amgen, AstraZeneca, Bayer, Biomea, Bristol Myers Squibb, Daiichi Sankyo, Deciphera, Eisai, Eli Lilly, Endeavor, Erasca, F. Hoffmann-La Roche, Fate Therapeutics, Genentech, Genmab, Immunogenesis, Infinity, Kyowa Kirin, Merck, Mirati, Navier, NCI-CTEP, Novartis, Numab, Pfizer, Pyramid Bio, Revolution Medicine, SeaGen, STCube, Takeda, TCR2, Turning Point Therapeutics, VM Oncology.

REFERENCES

1. Creelan, B.C., Wang, C., Teer, J.K., Toloza, E.M., Yao, J., Kim, S., Landin, A.M., Mullinax, J.E., Saller, J.J., Saltos, A.N., et al. (2021). Tumor-infiltrating lymphocyte treatment for anti-PD-1-resistant metastatic lung cancer: a phase 1 trial. Nat. Med. *27*, 1410–1418. https://doi.org/10.1038/s41591-021-01462-y.

2. Sarnaik, A.A., Hamid, O., Khushalani, N.I., Lewis, K.D., Medina, T., Kluger, H.M., Thomas, S.S., Domingo-Musibay, E., Pavlick, A.C., Whitman, E.D., et al. (2021). Lifileucel, a tumor-infiltrating lymphocyte therapy, in metastatic melanoma. J. Clin. Oncol. *39*, 2656–2666. https://doi.org/10.1200/JCO.21.00612.

3. Schuster, S.J., Bishop, M.R., Tam, C.S., Waller, E.K., Borchmann, P., McGuirk, J.P., Jäger, U., Jaglowski, S., Andreadis, C., Westin, J.R., et al. (2019). Tisagenlecleucel in adult relapsed or refractory diffuse large B-cell lymphoma. N. Engl. J. Med. *380*, 45–56. https://doi.org/10.1056/NEJMoa1804980.

4. Stevanovic, S., Draper, L.M., Langhan, M.M., Campbell, T.E., Kwong, M.L., Wunderlich, J.R., Dudley, M.E., Yang, J.C., Sherry, R.M., Kammula, U.S., et al. (2015). Complete regression of metastatic cervical cancer after treatment with human papillomavirus-targeted tumor-infiltrating T cells. J. Clin. Oncol. *33*, 1543–1550. https://doi.org/10.1200/JCO.2014.58.9093.

5. Zacharakis, N., Chinnasamy, H., Black, M., Xu, H., Lu, Y.C., Zheng, Z., Pasetto, A., Langhan, M., Shelton, T., Prickett, T., et al. (2018). Immune recognition of somatic mutations leading to complete durable regression in metastatic breast cancer. Nat. Med. *24*, 724–730.

6. Tsimberidou, A.M., Van Morris, K., Vo, H.H., Eck, S., Lin, Y.F., Rivas, J.M., and Andersson, B.S. (2021). T-cell receptor-based therapy: an innovative therapeutic approach for solid tumors. J. Hematol. Oncol. *14*, 102. https://doi.org/10.1186/s13045-021-01115-0.

7. Wang, M., and Claesson, M.H. (2014). Classification of human leukocyte antigen (HLA) supertypes. Methods Mol. Biol. *1184*, 309–317. https://doi.org/10.1007/978-1-4939-1115-8_17.

8. Dos Santos Francisco, R., Buhler, S., Nunes, J.M., Bitarello, B.D., França, G.S., Meyer, D., and Sanchez-Mazas, A. (2015). HLA supertype variation across populations: new insights into the role of natural selection in the evolution of HLA-A and HLA-B polymorphisms. Immunogenetics *67*, 651–663. https://doi.org/10.1007/s00251-015-0875-9.

9. Gragert, L., Madbouly, A., Freeman, J., and Maiers, M. (2013). Six-locus high resolution HLA haplotype frequencies derived from mixed-resolution DNA typing for the entire US donor registry. Hum. Immunol. *74*, 1313–1320. https://doi.org/10.1016/j.humimm.2013.06.025.

10. Maiers, M., Gragert, L., and Klitz, W. (2007). High-resolution HLA alleles and haplotypes in the United States population. Hum. Immunol. *68*, 779–788. https://doi.org/10.1016/j.humimm.2007.04.005.

11. Hickman, E.S., Lomax, M.E., and Jakobsen, B.K. (2016). Antigen selection for enhanced affinity T-cell receptor-based cancer therapies. J. Biomol. Screen *21*, 769–785. https://doi.org/10.1177/1087057116637837.

12. Bergeron, A., Picard, V., LaRue, H., Harel, F., Hovington, H., Lacombe, L., and Fradet, Y. (2009). High frequency of MAGE-A4 and MAGE-A9 expression in high-risk bladder cancer. Int. J. Cancer *125*, 1365–1371. https://doi.org/10.1002/ijc.24503.

13. Ishihara, M., Kageyama, S., Miyahara, Y., Ishikawa, T., Ueda, S., Soga, N., Naota, H., Mukai, K., Harada, N., Ikeda, H., and Shiku, H. (2020). MAGE-A4, NY-ESO-1 and SAGE mRNA expression rates and co-expression relationships in solid tumours. BMC Cancer *20*, 606. https://doi.org/10.1186/s12885-020-07098-4.

14. Iura, K., Kohashi, K., Ishii, T., Maekawa, A., Bekki, H., Otsuka, H., Yamada, Y., Yamamoto, H., Matsumoto, Y., Iwamoto, Y., and Oda, Y. (2017). MAGEA4 expression in bone and soft tissue tumors: its utility as a target for immunotherapy and diagnostic marker combined with NY-ESO-1. Virchows Arch. *471*, 383–392. https://doi.org/10.1007/s00428-017-2206-z.

15. Hong, D.S., Van Tine, B.A., Biswas, S., McAlpine, C., Johnson, M.L., Olszanski, A.J., Clarke, J.M., Araujo, D., Blumenschein, G.R., Jr., Kebriaei, P., et al. (2023). Autologous T cell therapy for MAGE-A4^{+} solid cancers in HLA-A*02^{+} patients: a phase 1 trial. Nat. Med. *29*, 104–114. https://doi.org/10.1038/s41591-022-02128-z.

16. Moreno Garcia, V., Calvo, E., Asch, A., Butler, M., Zugazagoitia, J., Charlson, J., Cervantes, A., Van Tine, B., Aggen, D., Clarke, J., et al. (2023). 1019O Clinical and translational data from the phase I SURPASS trial of ADP-A2M4CD8 T cell receptor (TCR) T cell therapy alone or combined with nivolumab in solid tumors. Ann. Oncol. *34*, S620–S621. https://doi.org/10.1016/j.annonc.2023.09.2158.

17. Sanderson, J.P., Crowley, D.J., Wiedermann, G.E., Quinn, L.L., Crossland, K.L., Tunbridge, H.M., Cornforth, T.V., Barnes, C.S., Ahmed, T., Howe, K., et al. (2020). Preclinical evaluation of an affinity-enhanced MAGE-A4-specific T-cell receptor for adoptive T-cell therapy. OncoImmunology *9*, 1682381. https://doi.org/10.1080/2162402X.2019.1682381.

18. Gonzalez-Galarza, F.F., McCabe, A., Santos, E.J.M.D., Jones, J., Takeshita, L., Ortega-Rivera, N.D., Cid-Pavon, G.M.D., Ramsbottom, K., Ghattaoraya, G., Alfirevic, A., et al. (2020). Allele frequency net database (AFND) 2020 update: gold-standard data classification, open access genotype data and new query tools. Nucleic Acids Res. *48*, D783–D788. https://doi.org/10.1093/nar/gkz1029.

19. National Marrow Donor Program (2023). Be the match. https://bethematchclinical.org/.

20. Weon, J.L., and Potts, P.R. (2015). The MAGE protein family and cancer. Curr. Opin. Cell Biol. *37*, 1–8. https://doi.org/10.1016/j.ceb.2015.08.002.

21. D'Angelo, S.P., Araujo, D.M., Abdul Razak, A.R., Agulnik, M., Attia, S., Blay, J.-Y., Carrasco Garcia, I., Charlson, J.A., Choy, E., Demetri, G.D., et al. (2024). Afamitresgene autoleucel for advanced synovial sarcoma and myxoid round cell liposarcoma (SPEARHEAD-1): an international, open-label, phase 2 trial. Lancet *403*, 1460–1471. https://doi.org/10.1016/S0140-6736(24)00319-2.

22. Yakirevich, E., Sabo, E., Lavie, O., Mazareb, S., Spagnoli, G.C., and Resnick, M.B. (2003). Expression of the MAGE-A4 and NY-ESO-1 cancer-testis antigens in serous ovarian neoplasms. Clin. Cancer Res. *9*, 6453–6460.

23. Brasseur, F., Rimoldi, D., Liénard, D., Lethé, B., Carrel, S., Arienti, F., Suter, L., Vanwijck, R., Bourlond, A., Humblet, Y., et al. (1995). Expression of *MAGE* genes in primary and metastatic cutaneous melanoma. Int. J. Cancer *63*, 375–380. https://doi.org/10.1002/ijc.2910630313.

24. Shigematsu, Y., Hanagiri, T., Shiota, H., Kuroda, K., Baba, T., Mizukami, M., So, T., Ichiki, Y., Yasuda, M., So, T., et al. (2010). Clinical significance of cancer/testis antigens expression in patients with non-small cell lung cancer. Lung Cancer *68*, 105–110. https://doi.org/10.1016/j.lungcan.2009.05.010.

25. Ritz, C., and Streibig, J.C. (2005). Bioassay analysis using R. J. Stat. Software *12*, 1–22. https://doi.org/10.18637/jss.v012.i05.

OMTM, Volume *32*

Supplemental information

Identifying MAGE-A4-positive tumors for TCR T cell therapies in HLA-A*02-eligible patients

Tianjiao Wang, Jean-Marc Navenot, Stavros Rafail, Cynthia Kurtis, Mark Carroll, Marian Van Kerckhoven, Sofie Van Rossom, Kelly Schats, Konstantinos Avraam, Robyn Broad, Karen Howe, Ashley Liddle, Amber Clayton, Ruoxi Wang, Laura Quinn, Joseph P. Sanderson, Cheryl McAlpine, Carly Carozza, Eric Pimpinella, Susan Hsu, Francine Brophy, Erica Elefant, Paige Bayer, Dennis Williams, Marcus O. Butler, Jeffrey M. Clarke, Justin F. Gainor, Ramaswamy Govindan, Victor Moreno, Melissa Johnson, Janet Tu, David S. Hong, and George R. Blumenschein Jr.

SUPPLEMENTAL TEXT

MAGE-A4 immunohistochemistry (IHC) clinical trial assay (CTA)

Samples

Tumor and normal tissue

Formalin-fixed paraffin-embedded (FFPE) tissue blocks were obtained in accordance with the Helsinki Declaration of 1975, following patient privacy procedures and approval by the hospital ethics committee (EC/PC/avl/2016.003) or purchased from different commercial providers (Proteogenex, QualTek Molecular Laboratories, Adaptimmune, ABS Bio, BioIVT, and Discovery Life Sciences).

Solid tumor indications (unique samples) used for MAGE-A4 validation (prevalence, precision, assay transfer, and inter-lot robustness): lung cancer (4), urinary bladder (2), HNSCC (33), ovarian cancer (44), gastric cancer (35), esophageal adenocarcinoma (31), esophageal squamous cell carcinoma (34), esophagastric junction adenocarcinoma (49), melanoma skin (2), synovial sarcoma (35), myxoid/round cell liposarcoma (35), endometrium carcinoma (37).

FFPE blocks used during the assay development and validation passed the following quality requirements: no tissue detachment, sufficient tissue, adequate staining (hematoxylin and eosin [H&E] or PTEN or ki67 IHC), and no impaired tissue integrity. Additionally, all tumor specimens were evaluated by a certified pathologist.

Cell lines

FFPE cell line slides (A375 and HCT116) with known expression levels of MAGE-A4 were procured, produced, characterized, and provided by Adaptimmune as run controls.

Additional cell lines were procured, produced, and characterized by Adaptimmune for specificity study of anti–MAGE-A4 monoclonal antibody (clone OTI1F9), including MAGE-As transduced NALM6 cell lines, NCI-H82, NCI-H466, Mel526, and Mel624, whose MAGE-As expression were shown by flow cytometry with Tomato Red reporter, quantitative reverse transcription polymerase chain reaction (RT-qPCR), or publicly available RNAseq database.

Tissue microarrays

TMA slides were purchased from US Biomax (MNO1021). All tissues were collected under the highest ethical standards, with the donor being informed completely and with their consent. US Biomax makes sure they follow standard medical care and protect the donors' privacy. Furthermore, all human tissues were collected under HIPAA-approved protocols and have been tested negative for HIV and hepatitis B and are approved for commercial product development.

Staining procedure

Before staining, slides were baked for 2 hours at 60°C and deparaffinized using the automated in pretreatment module: 3-in-1 specimen preparation procedure using TRS low pH antigen retrieval solution (K8005) (20 min, 97°C). The Envision detection system (EnVision+ System- HRP Labeled Polymer Anti-Mouse [Dako - K4001]) combined with a Dako Liquid DAB Substrate Chromogen System (Dako - K3468) was used for visualization. All stained slides were scanned as whole-slide images using a digital slide scanner (3DHISTECH, Budapest, Hungary).

Pathologist scoring

Slides were scored for the overall percentage of MAGE-A4–positive tumor cells and the intensity of MAGE-A4 staining. Only tumor cells were scored and any expression in surrounding stroma was ignored. All scoring was performed by a pathologist on either glass slides or high-resolution scanned whole-slide digital images.

Highly heterogenous tumors, such as synovial sarcoma and myxoid/round cell liposarcoma (MRCLS), require scoring in multiple high-power fields using the “field of view” method, while tumors with homogeneous MAGE-A4 expression are scored using regional method.

The percentage of MAGE-A4–positive tumor cells was determined at each intensity (0, 1+, 2+, and 3+ intensity) relative to the total number of viable tumor cells in the sample. When a MAGE-A4 signal was present in the cytoplasm and nucleus, the compartment with the highest-intensity expression was evaluated. Specific scoring rules were applied when scoring liposarcoma with myxoid regions and regions with complex arborizing vessel patterns. Arborizing vessels and the cell poor myxoid component were not taken into account when present. The high cellularity regions of the tumor were scored when scoring the myxoid liposarcoma cases.

A sample was considered MAGE-A4 positive if ≥30% tumor cells had a MAGE-A4 positivity at 2+ intensity or more (Figure S2).

Statistical analysis

A specific cutoff point (≥30% MAGE-A4–positive tumor cells stained at ≥2+ intensities) was applied on the scoring outcome to determine positivity/negativity for each sample. The positive/negative status was used to establish concordance. For precision assessment (intra- and inter-run variability), percent positive agreement and percent negative agreement of repeat staining of samples were based on the positive/negative status. The acceptance criterion for precision was set as 80% concordance at the slide level (nine replicates in three different runs for each of a minimum of four different samples per indication). Furthermore, the concordance on sample level is included for descriptive purposes.

Accuracy/specificity of the MAGE-A4 antibody

Several FFPE cell line slides and control tissue (normal testis) with known expression levels of MAGE-A4 were characterized to determine the MAGE-A4 specificity.

Cell line A375, which is known to be positive (datasheet Origene, clone OTI1F9; Sanderson et al., 2019) for MAGE-A4, demonstrated MAGE-A4–positive staining using the MAGE-A4 IHC CTA, while for cell line HCT116, known to be negative for MAGE-A4, no staining could be observed in all staining runs (Figure S3). In testis, nuclear and cytoplasmic MAGE-A4 staining was observed in the atrophic ducts and in the seminiferous tubules with strong intratubular staining, while no MAGE-A4 staining was demonstrated in the stroma (Figure S4).

No MAGE-A4 staining was observed in a normal human tissue TMA, except for human testis (Figure S5). The TMA with different (normal) human tissue types was evaluated for staining intensity and no positivity for MAGE-A4 could be observed in these normal tissues (breast, intestine, liver, lung, stomach, heart, fallopian tube) with a 10 µg/ml concentration of the primary antibody. For testis, nuclear and cytoplasmic MAGE-A4 positivity is present in the seminiferous tubules.

To further validate the specificity of anti–MAGE-A4 antibody, NALM6 parental cell line was transduced with different full-length MAGE-As (-A1, -A2, -A3, -A4, -A6, -A8, -A9, -A10). The expression of MAGE-As in NALM6-transduced cell lines was confirmed by flow cytometry with a reporter (Tomato Red) (Figure S6A). Anti–MAGE-A4 antibody stained specifically to MAGE-A4–transduced NALM6 cell line without cross-reactivity to MAGE-A1, -A2, -A3, -A6, and -A9. Rare staining (0.28%) by the anti–MAGE-A4 antibody was observed in MAGE-A8–transduced NALM6 cells (with a high MAGE-A8 expression level in >50% of cells), which is unlikely to change the diagnostic accuracy of an assay using the anti–MAGE-A4 antibody. Some low-level staining by the anti–MAGE-A4 antibody of MAGE-A10–transduced NALM6 cell line was also observed (Figure S6B).

To further investigate the cross-reactivity of anti–MAGE-A4 antibody to MAGE-A10, three cell lines (NCI-H82, NCI-H466, and Mel526) were chosen for further characterization. By qRT-PCR, NCI-H82 was shown to have high MAGE-A4 expression and high MAGE-A10 expression (MAGE-A4^{h}/MAGE-A10^{h}).

NCI-H466 was shown to have low MAGE-A4 expression and high MAGE-A10 expression (MAGE-A4^{l}/MAGE-A10^{h}). Mel526 was shown to have negligible MAGE-A4 expression and high MAGE-A10 expression (MAGE-A4^{n}/MAGE-A10^{h}) (Figure S6C). Anti–MAGE-A4 antibody showed strongly positive, weakly positive and negative staining in NCI-H82, NCI-H466, and Mel526, respectively, supporting the specificity of anti–MAGE-A4 antibody for MAGE-A4 without cross-reactivity to MAGE-A10 (Figure S6D). Some low-intensity staining could be observed with the anti–MAGE-A4 antibody in transduced NALM6 cells expressing extremely high levels of MAGE-A10 (Figure S6B), indicating a possible low-affinity cross-reactivity that can only be detected in this artificial system, which may not be physiologically or pathologically relevant.

Further evidence of the specificity of the MAGE-A4 CTA and estimation of the potential impact of the cross-reactivity with MAGE-A10 on its diagnostic value was provided by the analysis of data from 352 tumor samples from 10 different indications (melanoma, bladder cancer, NSCLC, head and neck cancer, esophageal cancer, esophagogastric junction cancer, ovarian cancer, gastric cancer, MRCLS, synovial sarcoma) stained for expression of both MAGE-A4 and MAGE-A10. These samples were screened under a screening protocol (ADP-0000-001, NCT02636855) used to determine eligibility for enrollment into one of two clinical trials using T cells directed against MAGE-A10 (ADP-0022-003, NCT02592577 and ADP-0022-004, NCT02989064) as well as a clinical trial using T cells directed against MAGE-A4 (ADP-0044-001, NCT03132922). For detection of MAGE-A10, a CTA based on a goat polyclonal antibody (Santa Cruz, Cat # sc-324906) was developed, validated, and used under a CLIA-certified laboratory to stain by IHC sections from FFPE tumor samples. Serial sections from the same samples were stained with the MAGE-A10 CTA. Similar to the MAGE-A4 CTA, scoring for the MAGE-A10 CTA was based on the percentage of live tumor cells stained at intensities of 0, 1+, 2+, or 3+. Most tested samples showed no expression of either target proteins. Figure S6E shows the P score (percentage of tumor cells stained at ≥2+) for both MAGE-A4 and MAGE-A10 in a selection of positively stained samples. Among the positively stained samples, the majority had expression of both MAGE-A4 and MAGE-A10 with similar levels. Four samples showed MAGE-A4 staining with a P score of 100 (with 3+ staining intensity in 70%–100% of tumor cells) but MAGE-A10 P score of 0, demonstrating the specificity of the anti–MAGE-A10 antibody, without cross-reactivity to MAGE-A4 (all the four data points overlapped and are shown as one in Figure S6E). Conversely, eight samples (Figure S6E, circled) showed P scores for MAGE-A10 between 20 and 100 (with 3+ staining intensity in 10%–70% of tumor cells) and no or very low staining for MAGE-A4 (P scores between 0 and 10). The low staining for MAGE-A4 observed in these samples could be due to actual low expression of MAGE-A4, but, even assuming that the MAGE-A4 signal is entirely due to cross-reactivity of the anti–MAGE-A4 antibody with MAGE-A10, this low cross-reactivity would not change the MAGE-A4 diagnosis status of these samples (positivity cutoff, ≥30% ≥2+), thus negating the risk of false positivity and confirming the diagnostic validity of the MAGE-A4 CTA.

In addition, anti–MAGE-A4 antibody staining by IHC showed no staining in Mel624 cell line, which is MAGE-A11 positive and MAGE-A12 positive by mRNA profile (Figure S6D). This further indicates the specificity of anti–MAGE-A4 antibody without cross-reactivity to MAGE-A11 and MAGE-A12.

MAGE-A4 prevalence

MAGE-A4 prevalence was assessed on a broad tissue sample set including a wide range of tumor indications (Table S1). For the determination of the prevalence of MAGE-A4 in different tumor indications, a MAGE-A4 cutoff of ≥30% at a ≥2+ intensity was applied. Feasibility of the MAGE-A4 assay was assessed in 316 tissue samples distributed over nine tumor indications covering the complete MAGE-A4 dynamic range (Figure S2, Table S1). Of the 316 tissue samples tested, 80 samples were positive for MAGE-A4. As demonstrated in Figure S2, the prevalence of MAGE-A4 ranged from 6% in EGJ cancer up to 67% in synovial sarcoma. Lower prevalence is observed in gastric cancer, EGJ cancer, MRCLS, ovarian cancer, and endometrium carcinoma, while in synovial sarcoma, HNSCC, and esophageal squamous cell carcinoma, and adenocarcinoma the prevalence is higher.

Robustness of the assay
To evaluate the robustness of the MAGE-A4 IHC CTA, precision testing (intra-run and inter-run), inter-lot (lot 1 vs. lot 2) and inter-lab (CellCarta BE vs. CellCarta US) comparison was evaluated to confirm the MAGE-A4 IHC CTA robustness regardless of the antibody lot used or the lab performing the MAGE-A4 assay. Each sample tested was stained for MAGE-A4 and their corresponding isotype control.

The MAGE-A4 IHC CTA robustness was evaluated both qualitatively and semi-quantitatively as scored by a pathologist. For each slide, the MAGE-A4 status (positivity cutoff: ≥30% tumor cells stained by MAGE-A4 at a ≥2+ intensity) was determined. The evaluation was based on the case status of each sample. All serial sections of each sample should be positive or negative in all the reads and an 80% overall concordance (overall percent agreement [OPA]) must be reached on slide level to consider the robustness as valid. The robustness results are summarized below.

Repeatability and reproducibility: precision
To evaluate the robustness (intra-run and inter-run) of the MAGE-A4 IHC assay, precision testing was performed in three independent staining runs on non-consecutive days on at least two Dako Link autostainer platforms by at least two different operators (Figure S7) to evaluate inter-run, intra-run, inter-operator, and inter-instrument variability. In each run, four serial sections were stained (three slides with the positive protocol and one with the negative protocol) from each block to evaluate the repeatability (intra-run) and reproducibility (inter-run) of the assay. Over three runs, 12 slides (nine with positive protocol and three with negative protocol) were stained per block.

Based on these results from the qualitative (Figures S8 and S9) and semi-quantitative (Table S2) evaluation, the precision of the MAGE-A4 assay was confirmed and each indication tested showed a concordance of >80%. Therefore, the MAGE-A4 assay was considered robust. Since different operators and instruments were used, the MAGE A4 assay is robust regardless of the operator or Dako Link autostainer instrument used for staining. MRCLS showed the lowest robustness (OPA 89%) since five slides from two samples deviated resulting in a different category. In the first sample, one slide was scored negative (22% at ≥2+ intensity), whereas the average score for this samples was 37% at ≥2+ intensity. In the second sample, four slides were scored negative (21%, 27%, 29%, and 29% at ≥2+ intensity), whereas the average score for this sample was 33% at ≥2+ intensity. Since the cutoff for MAGE-A4 is 30%, both samples were borderline cases, and all slides were scored around the cutoff. The variation on slide level was minimal (15% CV and 20% CV, respectively) but since categorization was used, this resulted in a different category.

Inter-lab variability
The MAGE-A4 assay was initially validated at CellCarta Antwerp (CC BE). After validation at CC BE, the MAGE-A4 assay was transferred to CellCarta Naperville (CC US). All of CellCarta's laboratories are CAP/CLIA certified. All staining platforms at all sites are cross validated twice per year.

For inter-lab variability between CellCarta Antwerp (CC BE) and CellCarta Naperville (CC US), two serial slides of 12 tissue samples (six sarcoma and six carcinoma samples) (Table S3) were stained at both labs and evaluated for concordance using the ≥30% positivity at a ≥2+ intensity cutoff.

As demonstrated in Table S3, one sample, a MRCLS, had a higher variability (CV 47% [2.53/5.36]) compared to the other samples (CV <10%) although the MAGE-A4 status remained unchanged. Biological variation between the two stained slides could lead to the observed variability of staining.

Based on the results, it has been concluded that the MAGE-A4 assay performs similarly regardless of the staining lab, CC BE or CC US. The robustness of the MAGE-A4 assay is therefore confirmed. Representative images of the inter-lab comparison are included in Figures S10–S13.

Inter-lot variability

To evaluate lot-to-lot variability, three different MAGE-A4 lots (A001, F001, and F002) were compared and evaluated for robustness. Lot-to-lot variability was tested on synovial sarcoma (5), MRCLS (4), melanoma (3), breast carcinoma (1), ovarian carcinoma (1), bladder carcinoma (1), lung carcinoma (1), laryngeal squamous cell carcinoma (1), and qualified batch run controls (MAGE-A4–positive and –negative cell pellet, normal tissue, and MAGE-A4–positive tumor sample). Serial slides of the tissue samples were stained with both lots and qualitatively and semi-quantitatively evaluated.

The results of inter-lot variability are presented in Figures S14–S16 and Table S4. In general, F001 showed a slightly weaker staining compared to A001 and F002. However, the scoring was not affected, and the lot-to-lot variability was considered valid.

Based on the results, it has been concluded that the MAGE-A4 assay performs similarly regardless of the MAGE-A4 antibody lot used for testing.

SUPPLEMENTAL TABLES

Table S1. MAGE-A4 prevalence data in commercially procured FFPE tissue carcinoma and sarcoma samples.

EGJ, esophagogastric junction; FFPE, formalin fixed paraffin embedded; MAGE-A4, melanoma-associated antigen A4.

Tumor indication	Tissue samples tested	Prevalence MAGE-A4 with 30% cutoff	Dynamic range % MAGE-A4 at 2+ and 3+ intensity		
			0%–25%	25%–35% (around the cutoff)	35%–100%
Synovial sarcoma	30	20/30 (67%)	10	1	19
Myxoid/round cell liposarcoma	31	4/31 (13%)	26	1	4
Ovarian	41	6/41 (15%)	33	3	5
Endometrium	37	6/37 (16%)	31	2	4
Gastric adenocarcinoma	34	5/34 (15%)	29	0	5
Esophageal squamous cell carcinoma	34	15/34 (44%)	19	2	13
EGJ	49	3/49 (6%)	46	1	2
Esophageal adenocarcinoma	30	6/30 (20%)	23	2	5
Head and neck squamous cell carcinoma	30	15/30 (50%)	15	0	15

Table S2. Tumor indications used for precision evaluation.

EGJ, esophagogastric junction carcinoma; HNSCC, head and neck squamous cell carcinoma; MAGE-A4, melanoma-associated antigen A4; MRCLS, myxoid/round cell liposarcoma.

Tumor indication	Number of samples tested for robustness	Overall concordance on slide level (total slide stained)	Dynamic range % MAGE-A4		
			0%–25%	25%–35% (around the cutoff)	35%–100%
Synovial sarcoma	5	100% (45/45)	1	2*	2
MRCLS	5	89% (40/45)	2	2*	1
Ovarian	10	100% (89/89)**	5	3	2
Endometrium carcinoma	5	100% (44/44)**	2	2	1
Esophageal adenocarcinoma	5	100% (45/45)	0	2	3
Esophageal squamous cell carcinoma	4	100% (36/36)	1	2	1
Gastric adenocarcinoma	5	100% (45/45)	2	0	3
HNSCC	9	100% (81/81)	2	3	4
EGJ	4	100% (36/36)	3	0	1
<u>Mix of other solid tumors:</u><br>Bladder carcinoma<br>Melanoma<br>Lung carcinoma	 2<br>2<br>4	100% (72/72)	0	1	7

*One sample from synovial sarcoma (average % MAGE-A4 at ≥2+: 36%) and one sample from MRCLS (average % MAGE-A4 at ≥2+: 37%) were counted as samples around the cutoff in precision evaluation since their MAGE-A4 scores fell closely enough although not strictly within 25%–35%.

**One slide could not be evaluated.

Table S3. Results of MAGE-A4 CTA assay transfer.

BE, Belgium; CTA, clinical trial assay; HNSCC, head and neck squamous cell carcinoma; MAGE-A4, melanoma-associated antogen A4; MRCLS, myxoid/round cell liposarcoma; SD, standard deviation; US, United States.

Tumor indication (n° samples tested)	Results MAGE-A4			
	Average % at ≥2+ intensity	SD	Status	Concordance US/BE
Melanoma (2)	95.00	0.00	Positive	100% concordant
	10.00	0.00	Negative	
Ovarian (1)	30.00	0.00	Positive	
Synovial sarcoma (4)	0.00	0.00	Negative	
	0.00	0.00	Negative	
	91.00	2.02	Positive	
	44.42	4.36	Positive	
Bladder carcinoma (1)	60.00	0.00	Positive	
HNSCC (1)	100.00	0.00	Positive	
Lung carcinoma (1)	67.50	3.54	Positive	
MRCLS (2)	5.36*	2.53	Negative*	
	7.00	0.24	Negative	

*An MRCLS sample with higher variability (CV 47% [2.53/5.36)] in % MAGE-A4 positivity compared to the 11 other carcinoma samples.

Table S4. Inter-lot robustness of the MAGE-A4 antibody.

MAGE-A4, melanoma-associated antigen A4; MRCLS, myxoid/round cell liposarcoma.

MAGE-A4 antibody lot	Tumor type	% positive cells at ≥2+ intensity	MAGE-A4 status
**Lot A001 vs. F001**			
A001	Synovial sarcoma	46	Positive
F001		39	Positive
A001	Synovial sarcoma	96	Positive
F001		90	Positive
A001	Synovial sarcoma	0	Negative
F001		0	Negative
A001	MRCLS	0	Negative
F001		0	Negative
A001	MRCLS	29	Negative
F001		25	Negative
A001	MRCLS	47	Positive
F001		32	Positive
A001	Melanoma	13	Negative
F001		12	Negative
A001	Breast cancer	0	Negative
F001		0	Negative
A001	Esophageal cancer	92	Positive
F001		83	Positive
**Lot F001 vs. F002**			
F001	Melanoma	95	Positive
F002		95	Positive
F001	Ovarian cancer	25	Negative
F002		25	Negative
F001	Synovial sarcoma	0	Negative
F002		0	Negative
F001	Synovial sarcoma	0	Negative
F002		0	Negative
F001	Urinary bladder cancer	65	Positive
F002		50	Positive
F001	Laryngeal squamous cell carcinoma	100	Positive
F002		100	Positive
F001	Melanoma	15	Negative
F002		10	Negative
F001	Lung squamous cell carcinoma	65	Positive
F002		75	Positive

Table S5. Prevalence of HLA-A*02 alleles in different races and ethnicities in patients screened by the NMDP.[1,2]

API, Asian or Pacific Islander; NMDP, National Marrow Donnor Program.

Numbers represent the percentage of individuals expressing each allele or group of alleles in the population of interest using the formula Pi = 2 x F – (F^2), where Pi is the percentage of individuals expressing the allele and F is the allele frequency.

Allele	NMDP European Caucasian (N = 1,242,890)	NMDP African American (N = 416,518)	NMDP Mexican or Chicano (N = 261,235)	NMDP API (2007, N = 3,542)	NMDP Chinese (N = 99,672)	NMDP Japanese (N = 24,582)
A*02:01	47.51	23.17	37.51	18.02	18.03	27.41
A*02:02	0.18	8.11	1.51	0.06	0.02	0.01
A*02:03	0.004	0.04	0.04	6.22	14.88	0.28
A*02:06	0.36	0.14	3.92	9.42	6.86	14.40
A*02:01 + 02:02 + 02:03 + 02:06	47.90	30.41	41.80	31.90	37.12	39.82

References:

1. Gragert, L., Madbouly, A., Freeman, J., Maiers, M. (2013). Six-locus high resolution HLA haplotype frequencies derived from mixed-resolution DNA typing for the entire US donor registry. Hum. Immunol. *74*, 1313–1320. https://doi.org/10.1016/j.humimm.2013.06.025

2. Maiers, M., Gragert, L., Klitz, W. (2007). High-resolution HLA alleles and haplotypes in the United States population. Hum. Immunol. *68*, 779–788. https://doi.org/10.1016/j.humimm.2007.04.005

Table S6. MAGE-A4 prevalence reported in this study in comparison to previous literature reports.

EGJ, esophagogastric junction; HNSCC, head and neck squamous cell carcinoma; IHC, immunohistochemistry; MRCLS, myxoid/round cell liposarcoma; NSCLC, non-small cell lung cancer; RT-qPCR, quantitative reverse transcription polymerase chain reaction; SyS, synovial sarcoma.

Cancer	This study (ADP-0000-001/ADP-0044-002)			Literature reports			
	MAGE-A4+ (%)	Assay	Positivity cutoff	MAGE-A4+ (%)	Assay	Positivity cutoff	Reference
SyS	70% (140/201)	IHC	≥30%, ≥2+	82% (89/108)	IHC	Total score ≥3	1
				53% (9/17)	IHC	≥5%, ≥1+	2
MRCLS	40% (27/67)	IHC	≥30%, ≥2+	0% (0/9)	IHC	≥5%, ≥1+	2
				68% (63/93)	IHC	Total score ≥3	3
Urothelial	32% (30/93)	IHC	≥30%, ≥2+	64% (60/94)	IHC	>0%, ≥1+	4
				42% (175/418)	IHC	>0%, ≥1+	5
				19% (281/1522)	IHC	>0%, ≥1+	6
EGJ	26% (24/93)	IHC	≥30%, ≥2+				N/A
Ovarian	24% (54/226)	IHC	≥30%, ≥2+	42% (31/74)	IHC	≥5%, ≥1+	7
				36% (106/294)	IHC	≥5%, ≥1+	8
HNSCC	22% (43/200)	IHC	≥30%, ≥2+	72% (63/88)	IHC	>0%, ≥1+	9
				24% (12/51)	RT-qPCR	Ct ≤30	9
				60% (34/57)	RT-qPCR	≥1% reference	10
				38% (27/72)	RT-qPCR	>12.2 copies/10^4 GAPDH	11
Esophageal	21% (21/100)	IHC	≥30%, ≥2+	55% (124/226)	RT-qPCR	>12.2 copies/10^4 GAPDH	11
				7% (3/46)	Microarray	≥ 2-fold of normal tissue	12
				<20% (12/59)	IHC	>0%, ≥2+	12
Melanoma	16% (39/243)	IHC	≥30%, ≥2+	<10% (4/47)	IHC	>0%, ≥1+	13

				9% (53/586)	IHC	>0%, ≥1+	14
NSCLC	14% (63/457)	IHC	≥30%, ≥2+	30% (47/159)	IHC	H score ≥100	15
				18% (12/67)	RT-qPCR	>12.2 copies/10^4 GAPDH	11
Gastric	9% (6/70)	IHC	≥30%, ≥2+	35% (7/20)	RT-qPCR	>12.2 copies/10^4 GAPDH	11

References:

1. Iura, K., Maekawa, A., Kohashi, K., Ishii, T., Bekki, H., Otsuka, H., Yamada, Y., Yamamoto, H., Harimaya, K., Iwamoto, Y., et al. (2017). Cancer-testis antigen expression in synovial sarcoma: NY-ESO-1, PRAME, MAGEA4, and MAGEA1. Hum. Pathol. *61*, 130–139. https://doi.org/10.1016/j.humpath.2016.12.006

2. Kakimoto, T., Matsumine, A., Kageyama, S., Asanuma, K., Matsubara, T., Nakamura, T., Iino, T., Ikeda, H., Shiku, H., Sudo, A. (2019). Immunohistochemical expression and clinicopathological assessment of the cancer testis antigens NY-ESO-1 and MAGE-A4 in high-grade soft-tissue sarcoma. Oncol. Lett. *17*, 3937–3943. https://doi.org/10.3892/ol.2019.10044

3. Iura, K., Kohashi, K., Ishii, T., Maekawa, A., Bekki, H., Otsuka, H., Yamada, Y., Yamamoto, H., Matsumoto, Y., Iwamoto, Y., et al. (2017). MAGEA4 expression in bone and soft tissue tumors: its utility as a target for immunotherapy and diagnostic marker combined with NY-ESO-1. Virchows Arch. *471*, 383–392. https://doi.org/10.1007/s00428-017-2206-z

4. Sharma, P., Shen, Y., Wen, S., Bajorin, D.F., Reuter, V.E., Old, L.J., Jungbluth, A.A. (2006). Cancer-testis antigens: expression and correlation with survival in human urothelial carcinoma. Clin. Cancer Res. *12*, 5442–5447. https://doi.org/10.1158/1078-0432.CCR-06-0527

5. Bergeron, A., Picard, V., LaRue, H., Harel, F., Hovington, H., Lacombe, L., Fradet, Y. (2009). High frequency of MAGE-A4 and MAGE-A9 expression in high-risk bladder cancer. Int. J. Cancer *125*, 1365–1371. https://doi.org/10.1002/ijc.24503

6. Kocher, T., Zheng, M., Bolli, M., Simon, R., Forster, T., Schultz-Thater, E., Remmel, E., Noppen, C., Schmid, U., Ackermann, D., et al. (2002). Prognostic relevance of MAGE-A4 tumor antigen expression in transitional cell carcinoma of the urinary bladder: a tissue microarray study. Int. J. Cancer *100*, 702–705. https://doi.org/10.1002/ijc.10540

7. Yakirevich, E., Sabo, E., Lavie, O., Mazareb, S., Spagnoli, G.C., Resnick, M.B. (2003). Expression of the MAGE-A4 and NY-ESO-1 cancer-testis antigens in serous ovarian neoplasms. Clin. Cancer Res. *9*, 6453–6460.

8. Daudi, S., Eng, K.H., Mhawech-Fauceglia, P., Morrison, C., Miliotto, A., Beck, A., Matsuzaki, J., Tsuji, T., Groman, A., Gnjatic, S., et al. (2014). Expression and immune responses to MAGE antigens predict survival in epithelial ovarian cancer. PLoS One *9*, e104099. https://doi.org/10.1371/journal.pone.0104099

9. Baran, C.A., Agaimy, A., Wehrhan, F., Weber, M., Hille, V., Brunner, K., Wickenhauser, C., Siebolts, U., Nkenke, E., Kesting, M., et al. (2019). MAGE-A expression in oral and laryngeal leukoplakia predicts malignant transformation. Mod. Pathol. *32*, 1068–1081. https://doi.org/10.1038/s41379-019-0253-5

10. Cuffel, C., Rivals, J.P., Zaugg, Y., Salvi, S., Seelentag, W., Speiser, D.E., Lienard, D., Monnier, P., Romero, P., Bron, L., et al. (2011). Pattern and clinical significance of cancer-testis gene expression in head and neck squamous cell carcinoma. Int. J. Cancer *128*, 2625–2634. https://doi.org/10.1002/ijc.25607

11. Ishihara, M., Kageyama, S., Miyahara, Y., Ishikawa, T., Ueda, S., Soga, N., Naota, H., Mukai, K., Harada, N., Ikeda, H., et al. (2020). MAGE-A4, NY-ESO-1 and SAGE mRNA expression rates and co-expression relationships in solid tumours. BMC Cancer *20*, 606. https://doi.org/10.1186/s12885-020-07098-4

12. Lin, J., Lin, L., Thomas, D.G., Greenson, J.K., Giordano, T.J., Robinson, G.S., Barve, R.A., Weishaar, F.A., Taylor, J.M., Orringer, M.B., et al. (2004). Melanoma-associated antigens in esophageal adenocarcinoma: identification of novel MAGE-A10 splice variants. Clin. Cancer Res. *10*, 5708–5716. https://doi.org/10.1158/1078-0432.CCR-04-0468

13. Errington, J.A., Conway, R.M., Walsh-Conway, N., Browning, J., Freyer, C., Cebon, J., Madigan, M.C. (2012). Expression of cancer-testis antigens (MAGE-A1, MAGE-A3/6, MAGE-A4, MAGE-C1 and NY-ESO-1) in primary human uveal and conjunctival melanoma. Br. J. Ophthalmol. *96*, 451–458. https://doi.org/10.1136/bjophthalmol-2011-300432

14. Barrow, C., Browning, J., MacGregor, D., Davis, I.D., Sturrock, S., Jungbluth, A.A., Cebon, J. (2006). Tumor antigen expression in melanoma varies according to antigen and stage. Clin. Cancer Res. *12*, 764–771. https://doi.org/10.1158/1078-0432.CCR-05-1544

15. Su, C., Xu, Y., Li, X., Ren, S., Zhao, C., Hou, L., Ye, Z., Zhou, C. (2015). Predictive and prognostic effect of CD133 and cancer-testis antigens in stage Ib-IIIA non-small cell lung cancer. Int. J. Clin. Exp. Pathol. *8*, 5509–5518.

SUPPLEMENTAL FIGURES

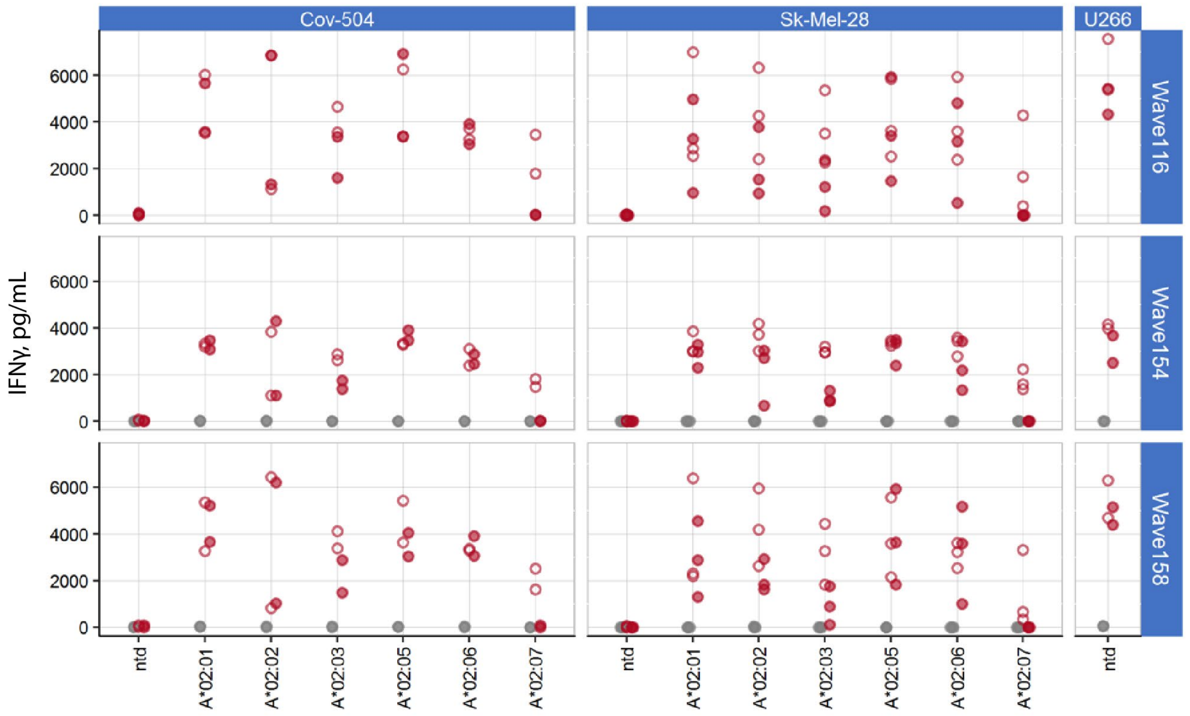

Figure S1. IFNγ release by afami-cel in response to MAGE-A4–positive cell lines expressing different HLA-A2 subtypes.

Cov-504 and Sk-Mel-28 (MAGE-A4 positive, HLA-A2 negative) cells were transduced to express HLA-A*02:01, HLA-A*02:02, HLA-A*02:03, HLA-A*02:05, HLA-A*02:06 and HLA-A*02:07 and subsequently used as targets for three separate batches of afami-cel (red points) or donor-matched non-transduced T cells (gray points; not available for Wave116). The natively HLA-A*02:01–positive and MAGE-A4–positive cell line U266 was included as a positive control. Closed circles show recognition (IFNγ release pg/ml) of endogenous MAGE-A4, open circles show recognition of cell lines exogenously loaded with 10^{-5} M synthetic MAGE-A4$_{230\text{-}239}$ peptide (IFNγ release pg/ml). Data are facetted vertically by T-cell donor. HLA, human leukocyte antigen; IFN, interferon; MAGE-A4, melanoma-associated antigen A4; ntd, non-transduced.

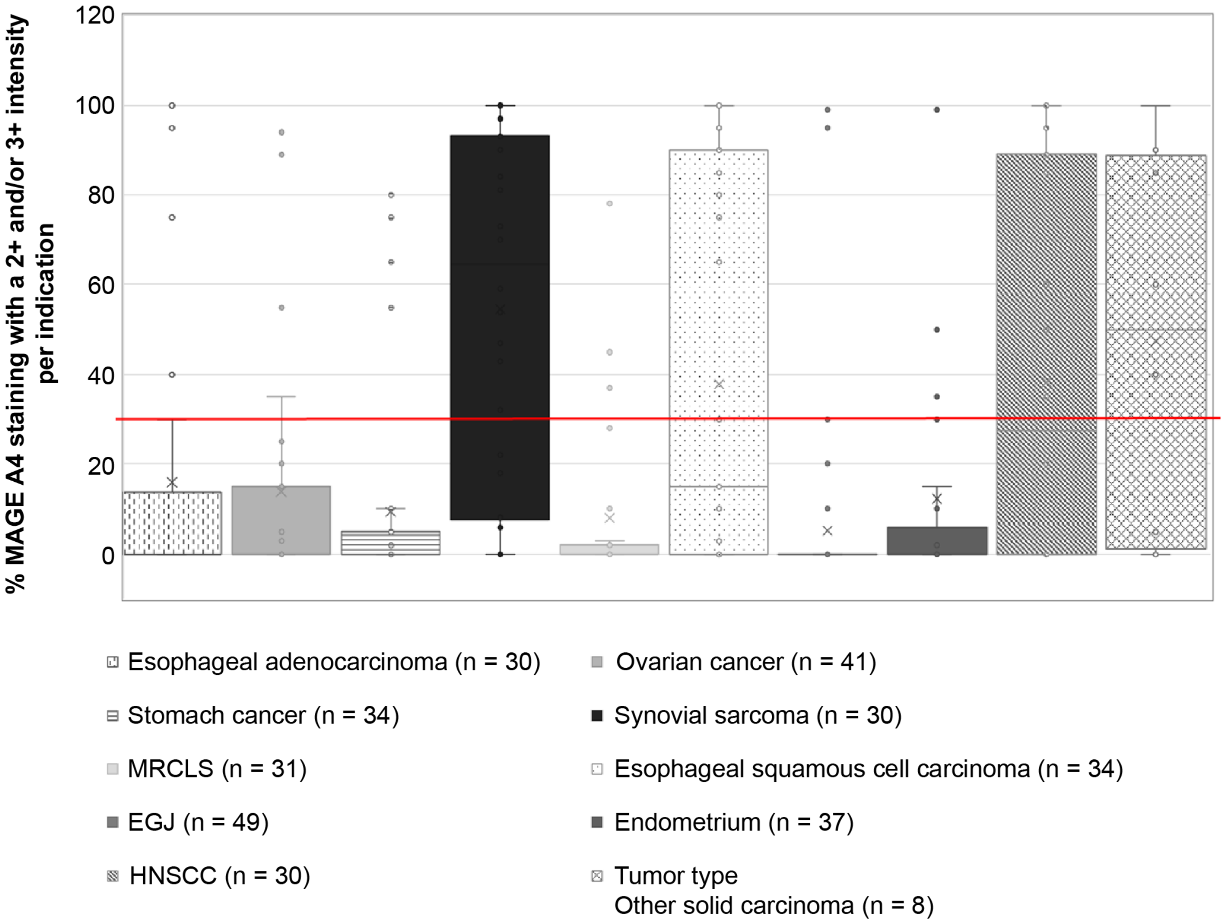

Figure S2. Distribution of the % MAGE-A4 positivity per tumor indication. Cutoff for MAGE-A4 positivity is indicated by a red horizontal line at 30%. The number of samples tested is indicated between brackets (n). EGJ, esophagogastric junction carcinoma; HNSCC, head and neck squamous cell carcinoma; MAGE-A4, melanoma-associated antigen A4; MRCLS, myxoid/round cell liposarcoma.

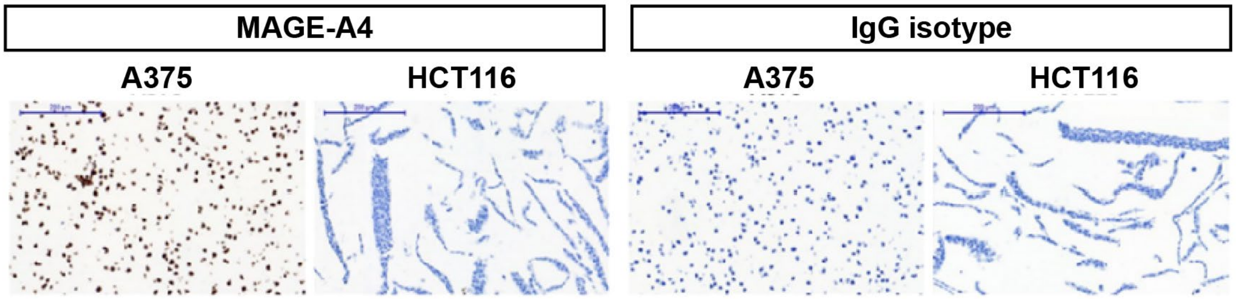

Figure S3. MAGE-A4 expression in control cell lines A375 (positive) and HCT116 (negative).

IgG, immunoglobulin G; MAGE-A4, melanoma-associated antigen A4.

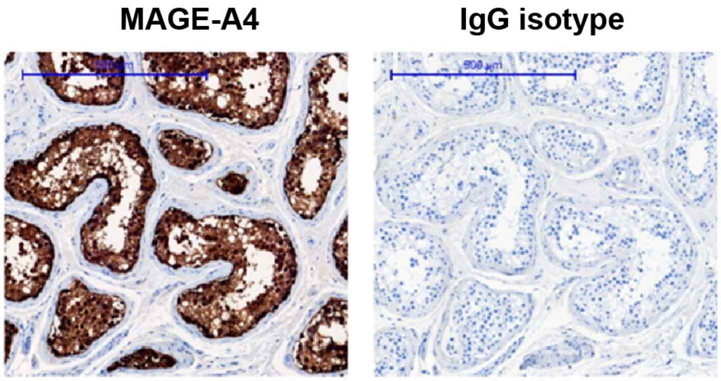

Figure S4. MAGE-A4 expression in control tissue (testis).

IgG, immunoglobulin G; MAGE-A4, melanoma-associated antigen A4.

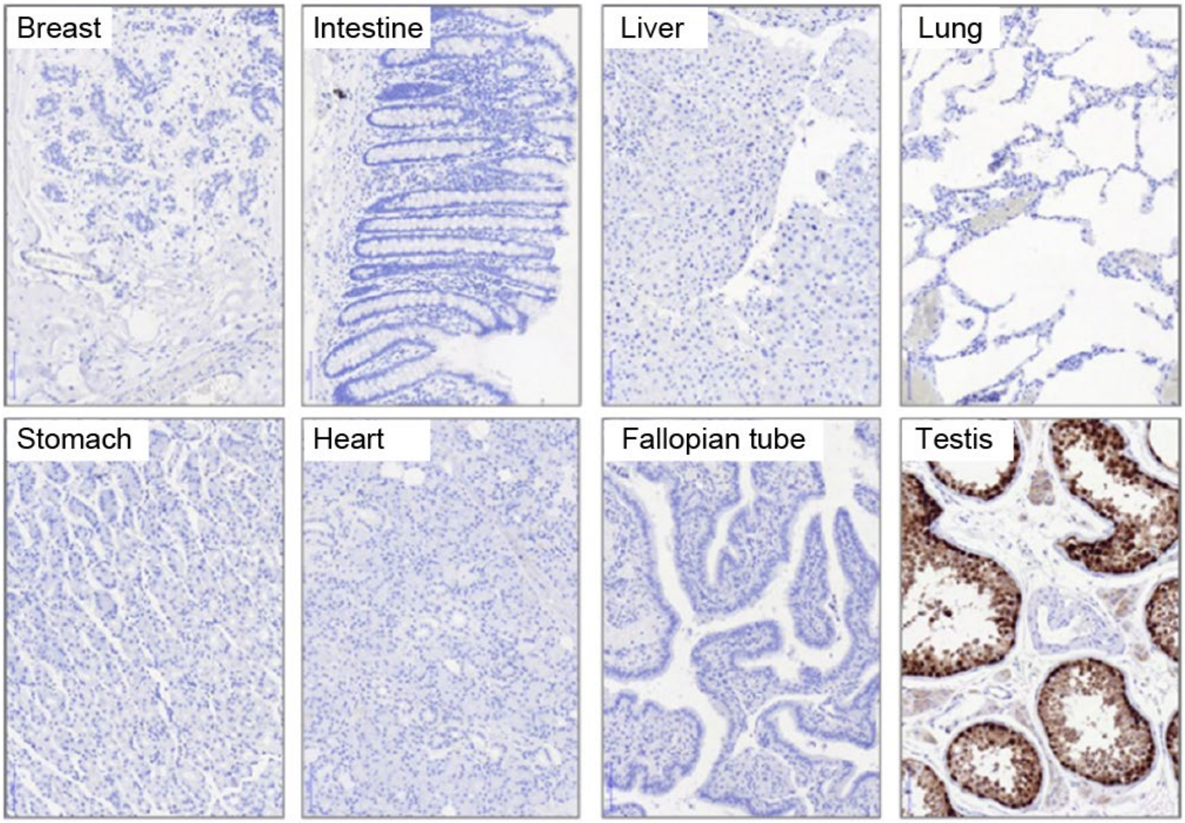

Figure S5. MAGE-A4 expression in normal tissues.

MAGE-A4, melanoma-associated antigen A4.

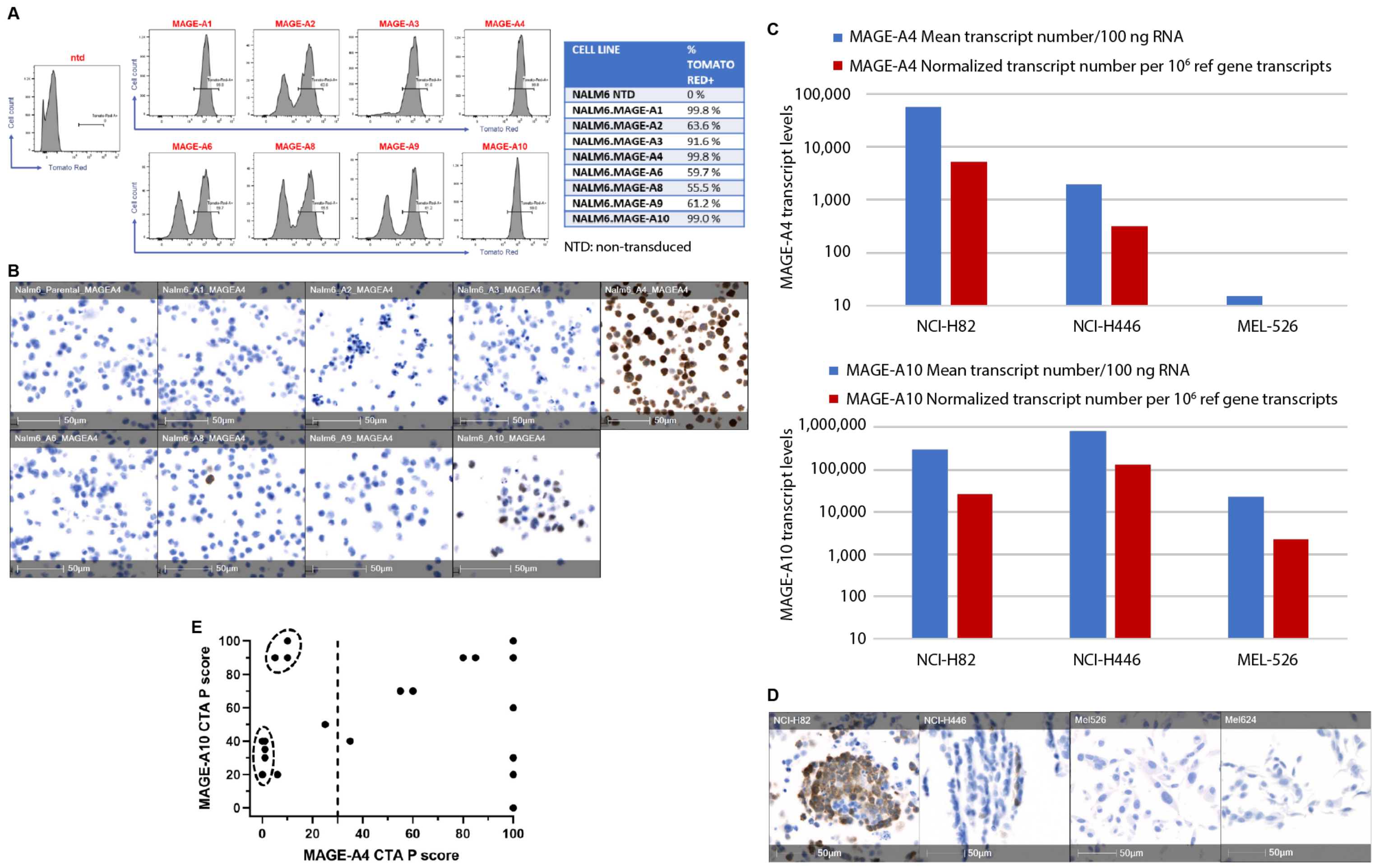

Figure S6. Anti–MAGE-A4 antibody specificity.

(A) MAGE-As expression in MAGE-As transduced NALM6 cell lines as determined by flow cytometry detection of the reporter protein Tomato Red. (B) IHC staining with anti–MAGE-A4 antibody of transduced NALM6 cell lines expressing various MAGE-As (A1, A2, A3, A4, A6, A8, A9 ,and A10). (C) MAGE-A4 and MAGE-A10 expression in NCI-H82, NCI-H466, and Mel526 cell lines as determined by qRT-PCR. (D) IHC staining with anti–MAGE-A4 antibody of NCI-H82 (MAGE-A4 high/MAGE-A10 high), NCI-H466 (MAGE-A4 low/MAGE-A10 high), Mel526 (MAGE-A4 negligible/MAGE-A10 high), and Mel624 (MAGE-A11+, MAGE-A12+) cell lines. (E) Tumor tissues stained and scored by both MAGE-A4 IHC CTA and MAGE-A10 IHC CTA. Dash vertical line showed the MAGE-A4 positivity cutoff (≥30% staining at ≥2+ intensities). The circled samples were highlighted to illustrate the negligible impact of MAGE-A10 expression on the determination of MAGE-A4 diagnosis. CTA, clinical trial assay; IHC, immunohistochemistry; MAGE-A4, melanoma-associated antigen A4; ntd, non-transduced; RT-PCR, reverse transcription polymerase chain reaction.

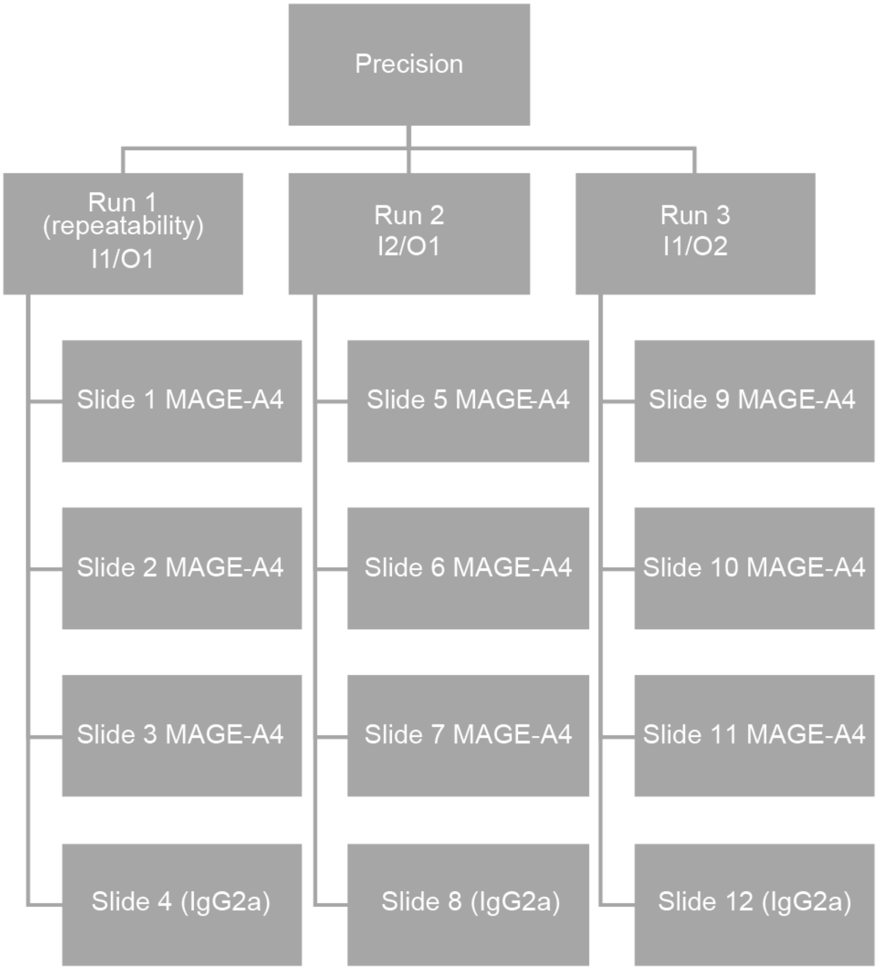

Figure S7. Diagram illustrating the robustness assessment strategy.

IgG, immunoglobulin G; MAGE A4, melanoma-associated antigen A4.

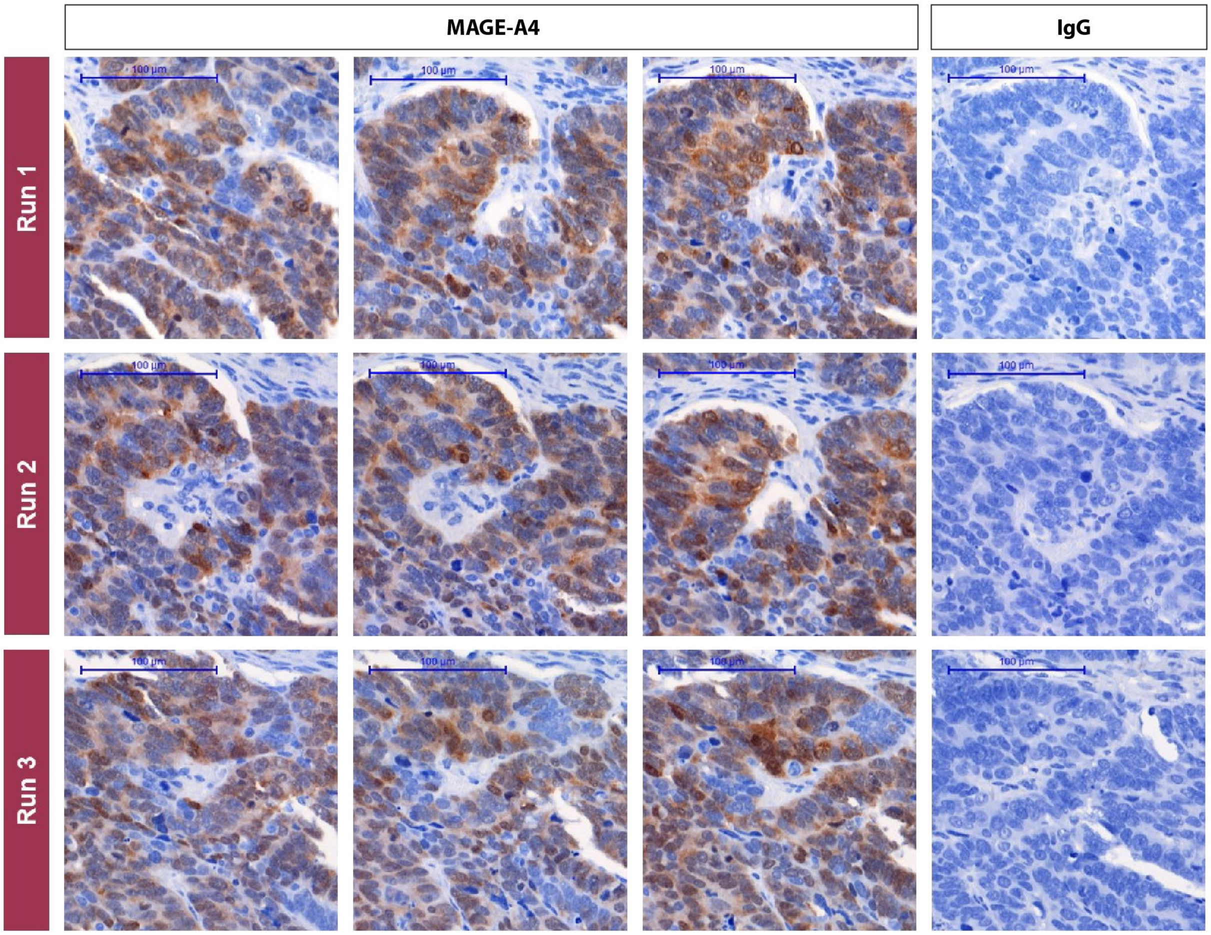

Figure S8. Representative detail images of the precision assessment of MAGE-A4 on a negative ovarian cancer tissue with 20% MAGE-A4 positivity at ≥2+ intensity during sensitivity screening.

Three serial sections of each sample were stained in each run for MAGE-A4. In each run, one slide was stained for the isotype IgG control. Repeatability was assessed in run 1, reproducibility was evaluated between the three different runs. 10x magnification. IgG, immunoglobulin G; MAGE-A4, melanoma-associated antigen A4.

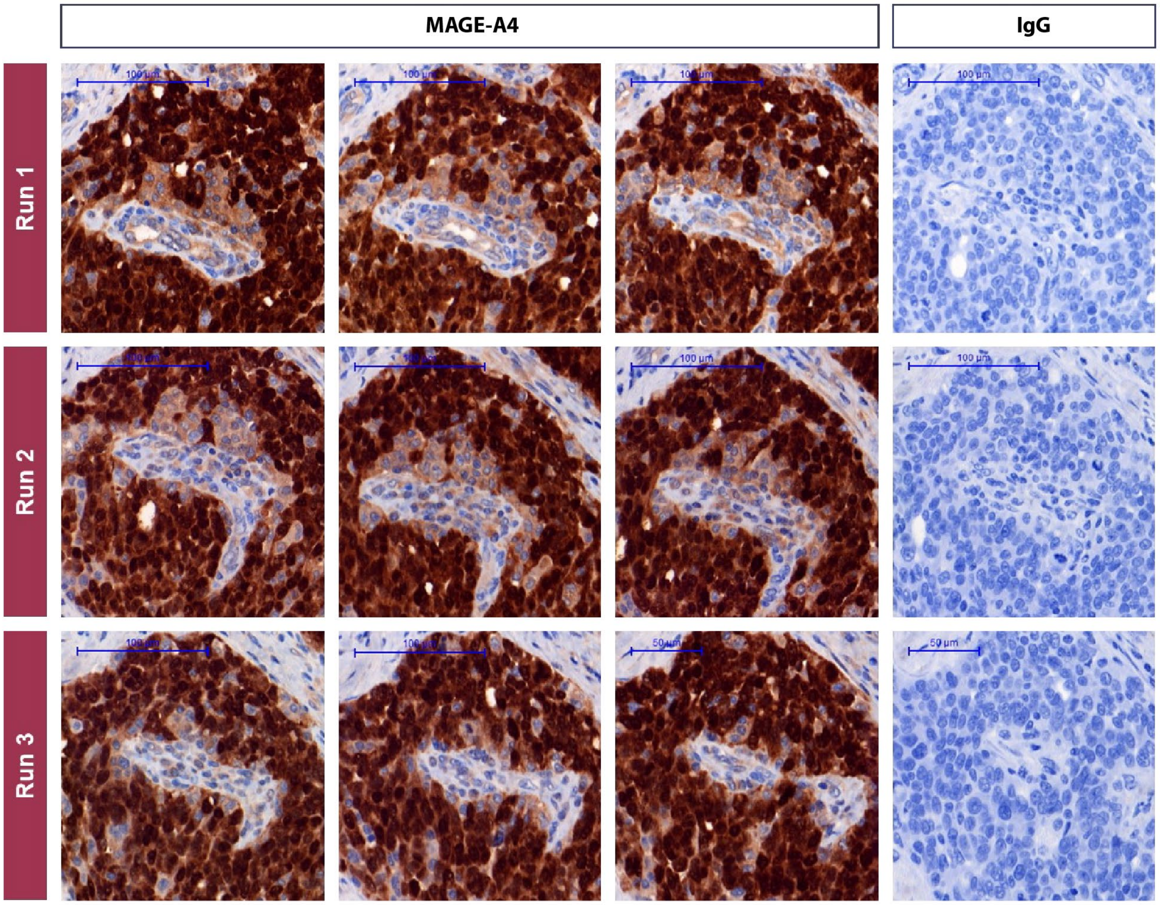

Figure S9. Representative detail images of the precision assessment of MAGE-A4 on a positive endometrium cancer tissue with a 94% MAGE-A4 positivity at ≥2+ intensity during sensitivity screening.

Three serial sections of each sample were stained in each run for MAGE-A4. In each run, one slide was stained for the isotype IgG control. Repeatability was assessed in run 1, reproducibility was evaluated between the three different runs. 20x magnification. IgG, immunoglobulin G; MAGE-A4, melanoma-associated antigen A4.

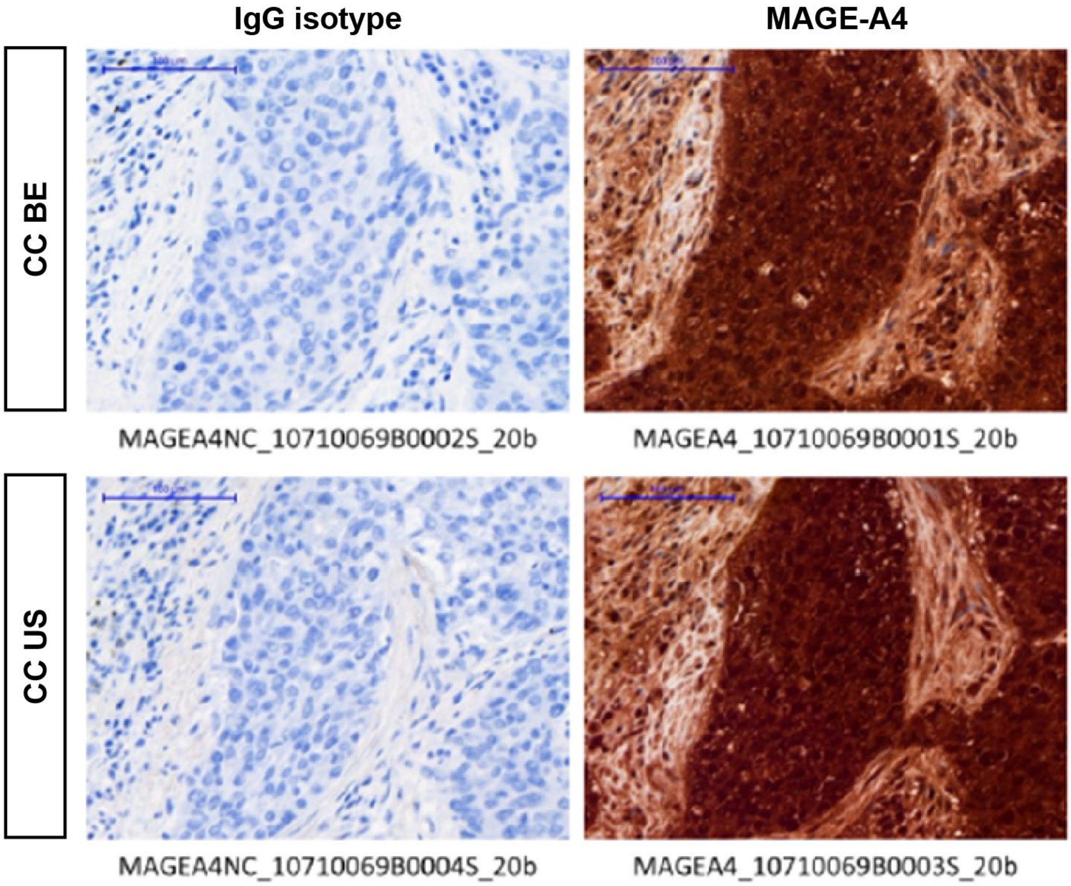

Figure S10. Representative images from lung squamous cell carcinoma stained at CC BE (top) and CC US (bottom) with the MAGE-A4 primary antibody (right) and the IgG isotype control (left).

BE, Belgium; CC, CellCarta, IgG, immunoglobulin G; MAGE-A4, melanoma-associated antigen A4; US, United States.

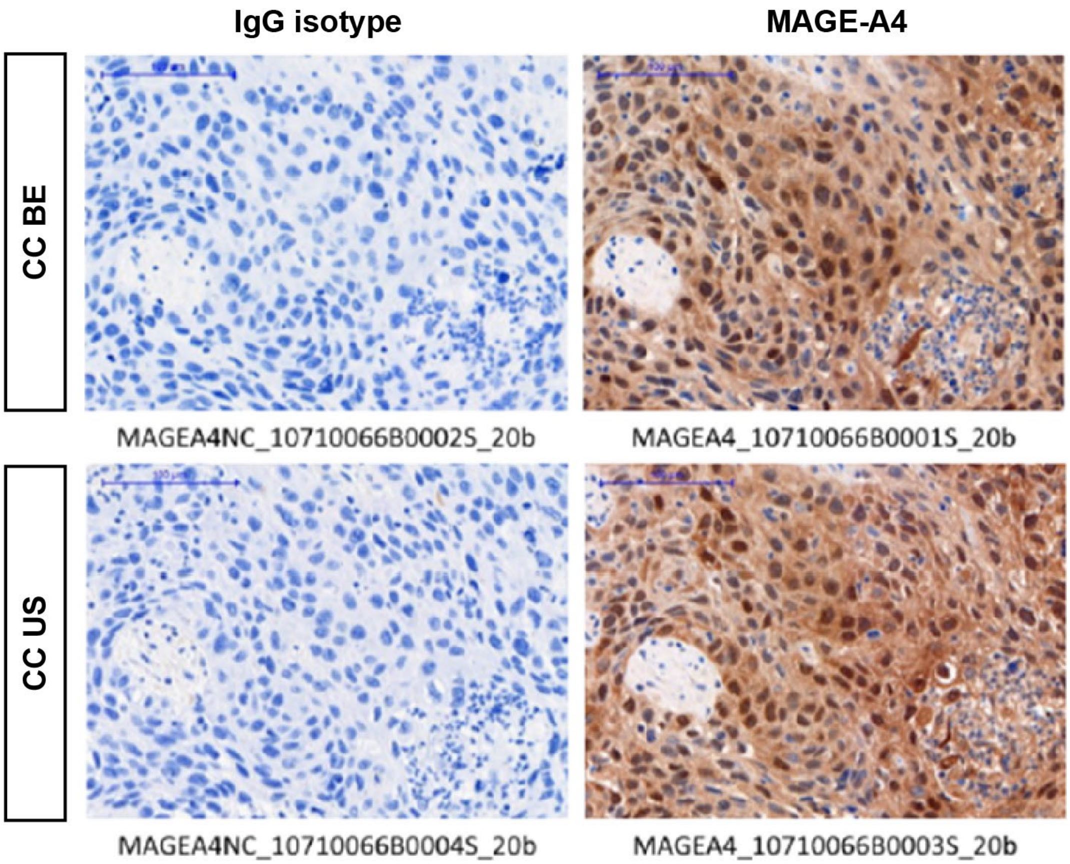

Figure S11. Representative images from urinary bladder cancer stained at CC BE (top) and CC US (bottom) with the MAGE-A4 primary antibody (right) and the IgG isotype control (left).

BE, Belgium; CC, CellCarta, IgG, immunoglobulin G; MAGE-A4, melanoma-associated antigen A4; US, United States.

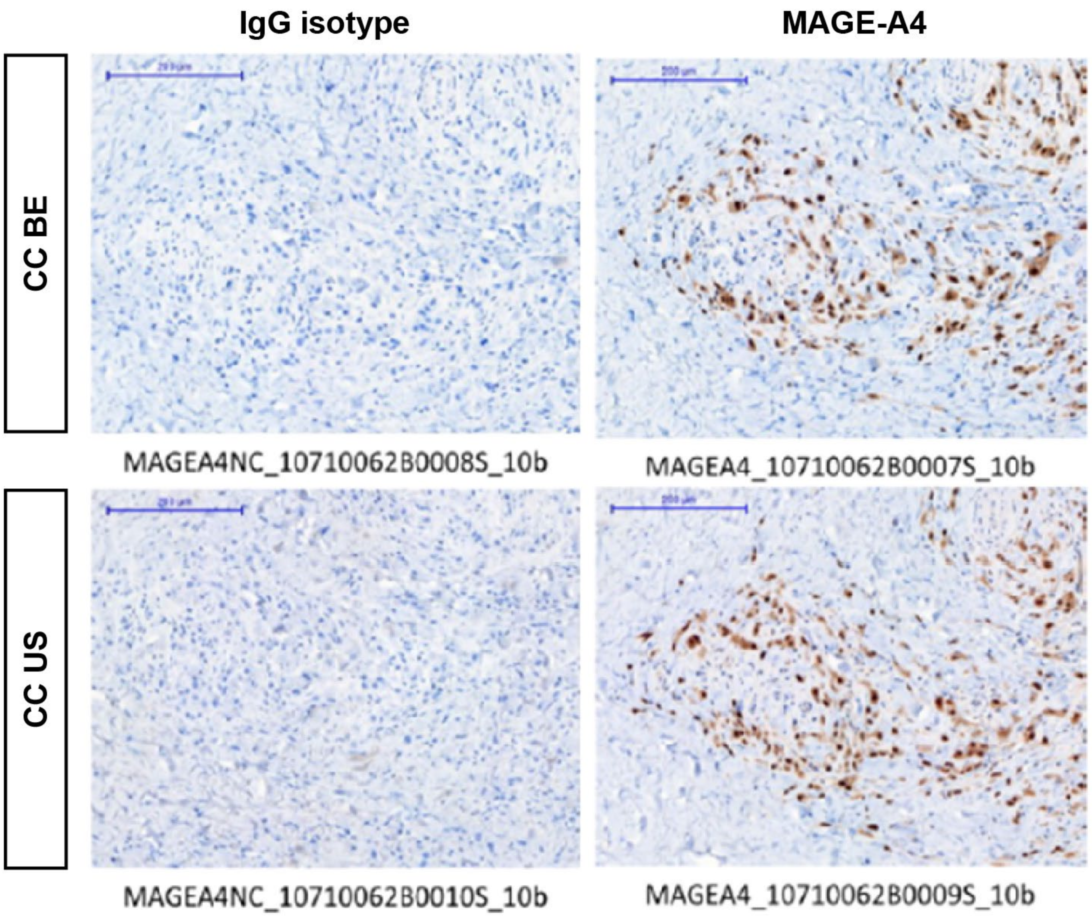

Figure S12. Representative images from melanoma cancer stained at CC BE (top) and CC US (bottom) with the MAGE-A4 primary antibody (right) and the IgG isotype control (left).

BE, Belgium; CC, CellCarta, IgG, immunoglobulin G; MAGE-A4, melanoma-associated antigen A4; US, United States.

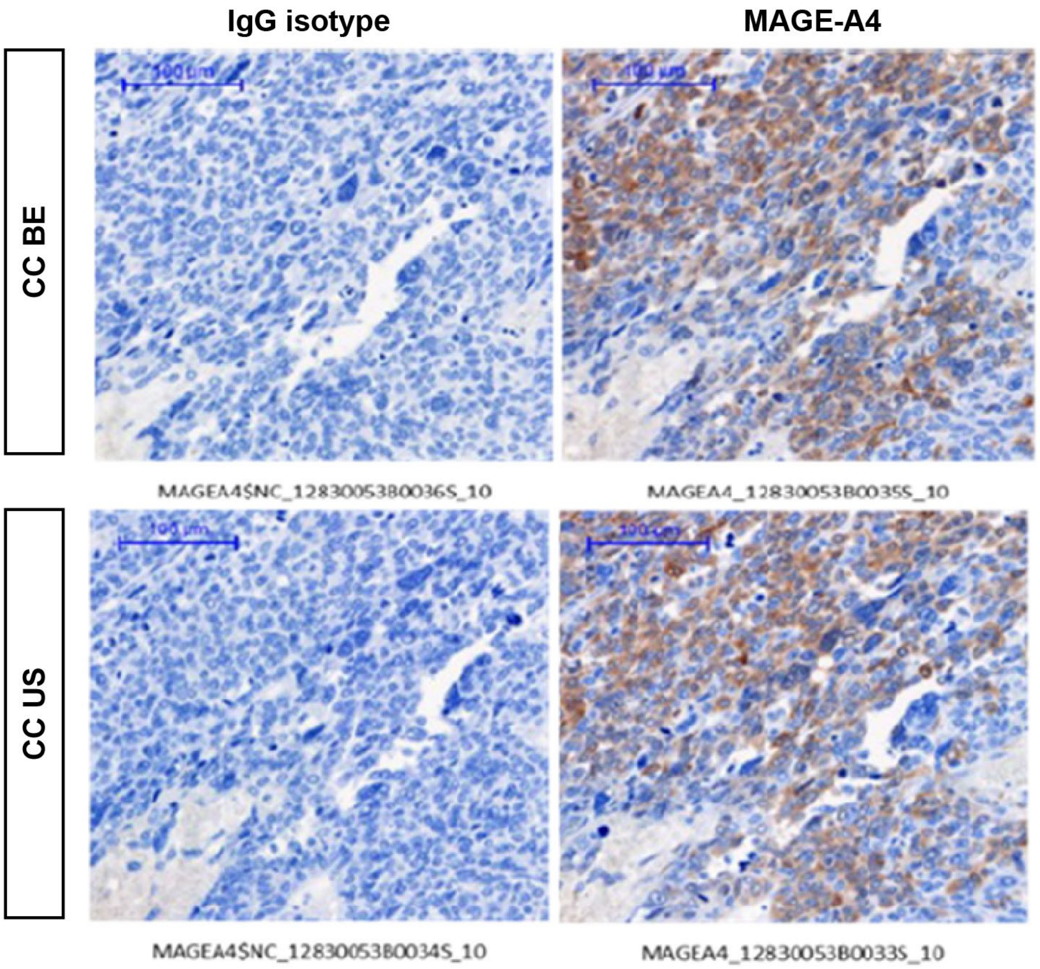

Figure S13. Representative images from myxoid/round cell liposarcoma stained at CC BE (top) and CC US (bottom) with the MAGE-A4 primary antibody (right) and the IgG isotype control (left).

BE, Belgium; CC, CellCarta, IgG, immunoglobulin G; MAGE-A4, melanoma-associated antigen A4; US, United States.

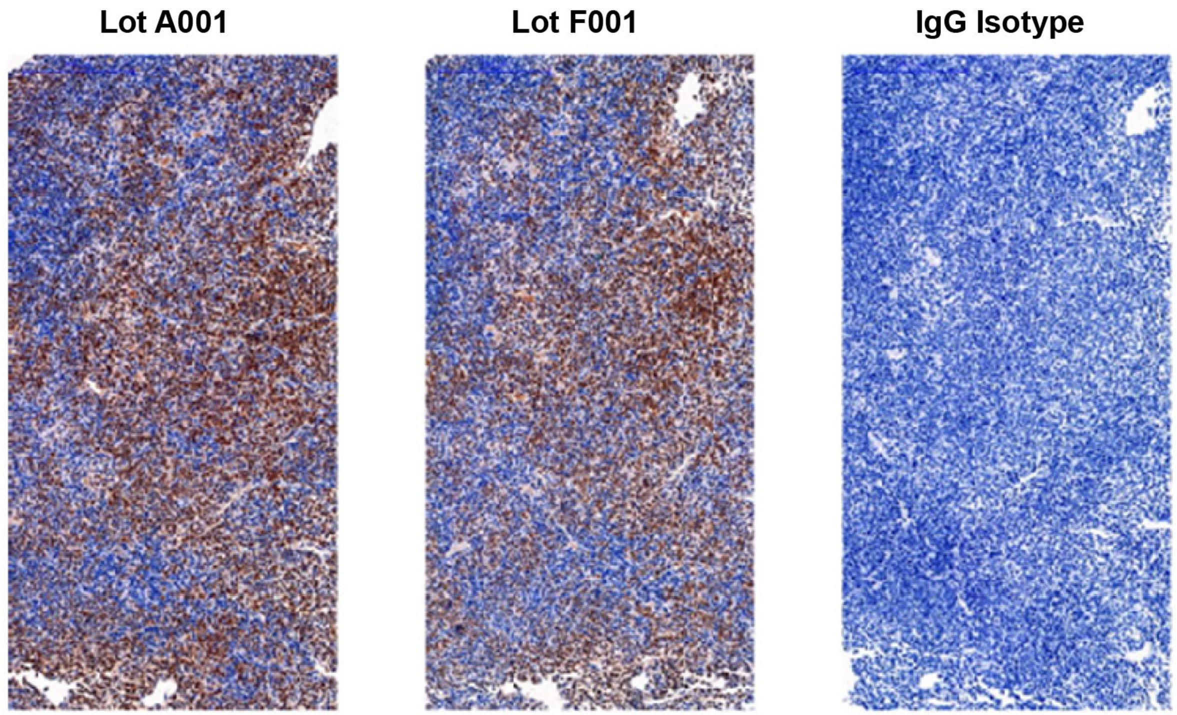

Figure S14. Serial slides of synovial sarcoma stained for MAGE-A4 using different antibody lots (A001 and F001).

No immunoreactivity is detected in the negative IgG controls. IgG, immunoglobulin G; MAGE-A4, melanoma-associated antigen A4.

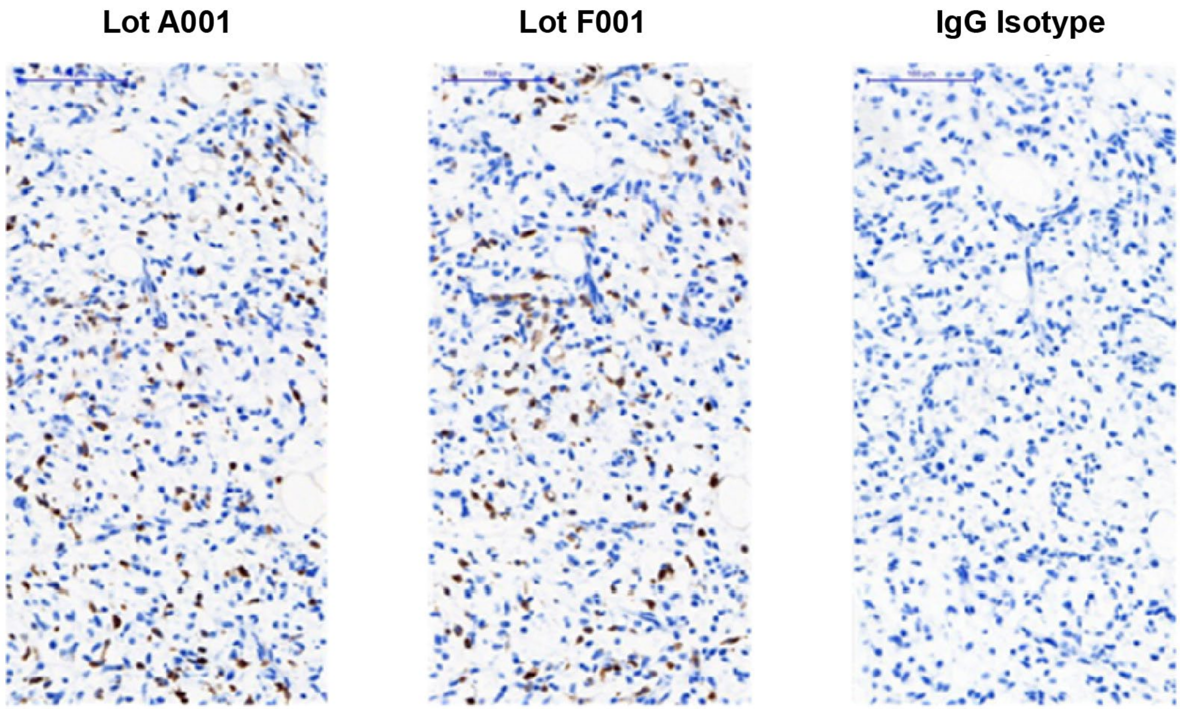

Figure S15. Serial slides of MRCLS stained for MAGE-A4 using different antibody lots (A001 and F001).

No immunoreactivity is detected in the negative IgG controls. IgG, immunoglobulin G; MAGE-A4, melanoma-associated antigen A4; MRCLS, myxoid/round cell liposarcoma.

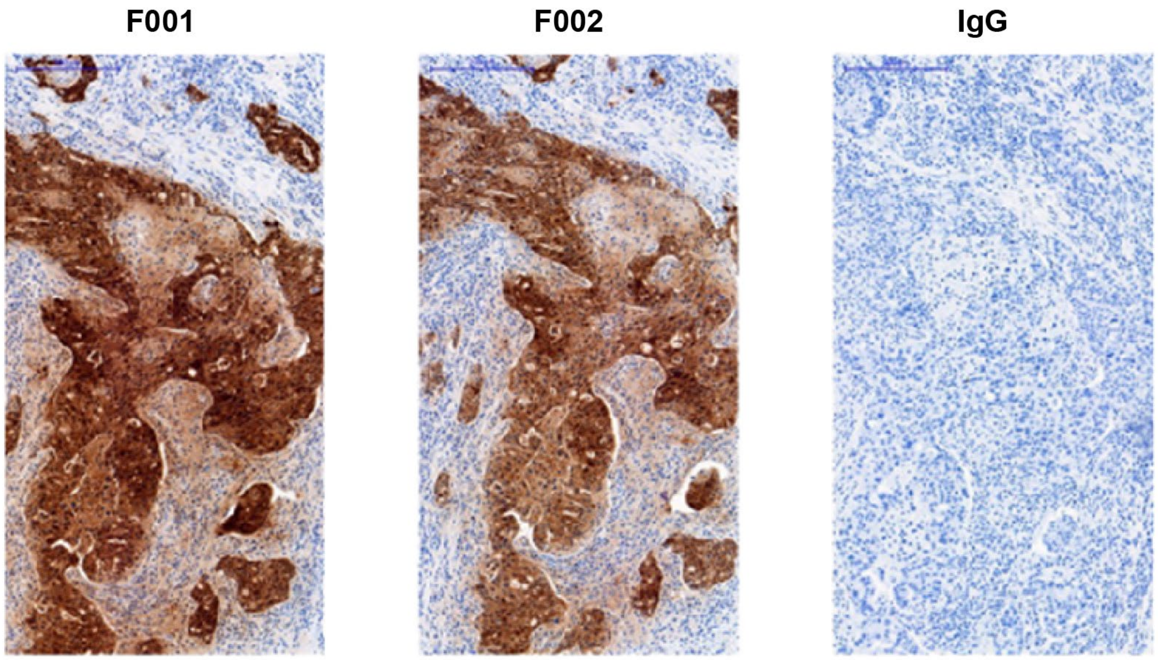

Figure S16. Serial slides of ovarian cancer stained for MAGE-A4 using different antibody lots (F001 and F002).

No immunoreactivity is detected in the negative IgG controls. IgG, immunoglobulin; MAGE-A4, melanoma-associated antigen A4.

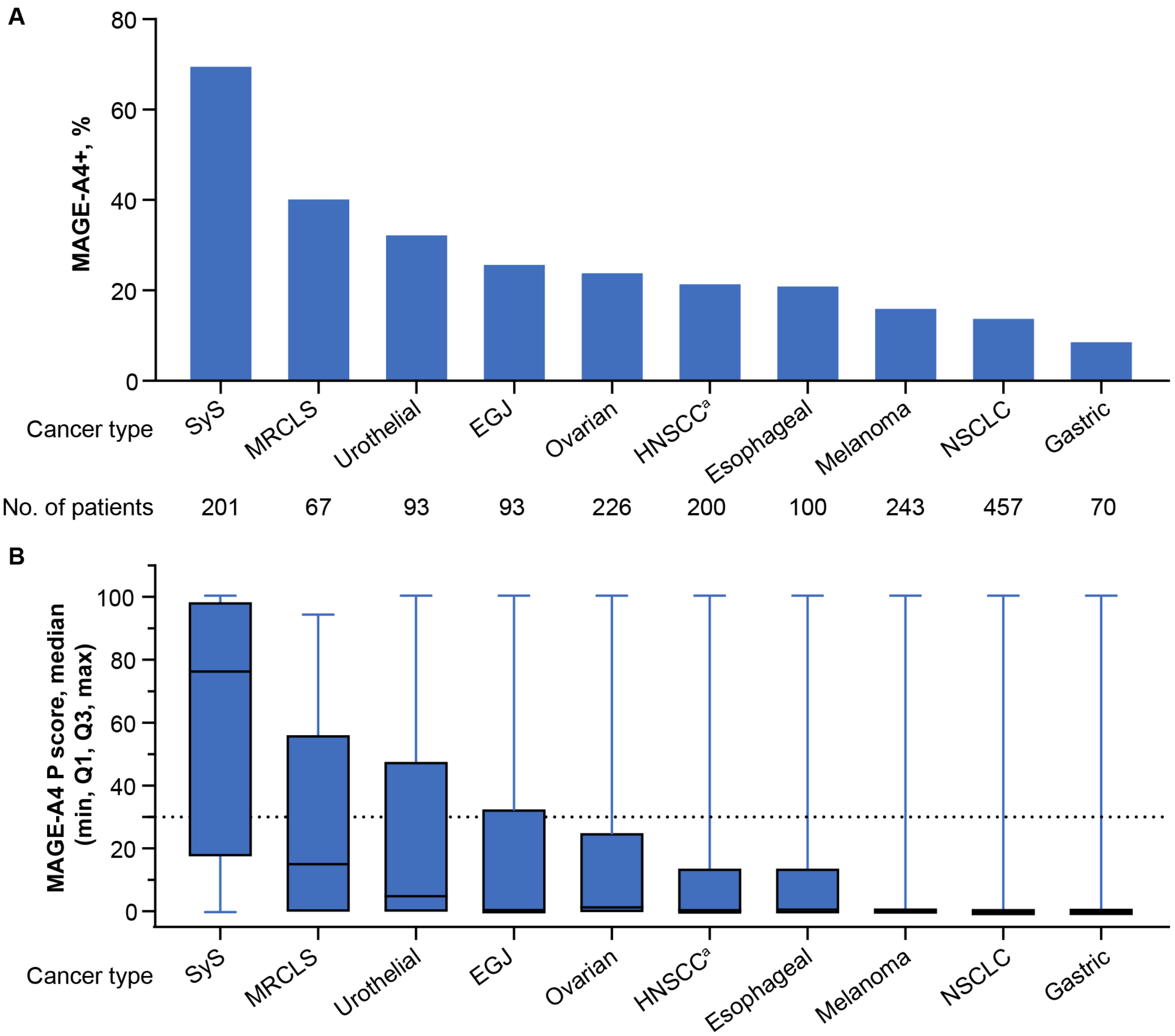

Figure S17. MAGE-A4 positivity and expression level by cancer type.

(A) MAGE-A4 positivity. (B) MAGE-A4 expression level. [a]n = 199 HNSCC, n = 1 “other” head and neck cancer histology. The dotted line represents the cutoff value of the P score indicating MAGE-A4 positivity. EGJ, esophagogastric junction cancer; HNSCC, head and neck squamous cell carcinoma; MAGE-A4, melanoma-associated antigen A4; MRCLS, myxoid/round cell liposarcoma; NSCLC, non-small cell lung cancer; P score, protein score; SyS, synovial sarcoma.

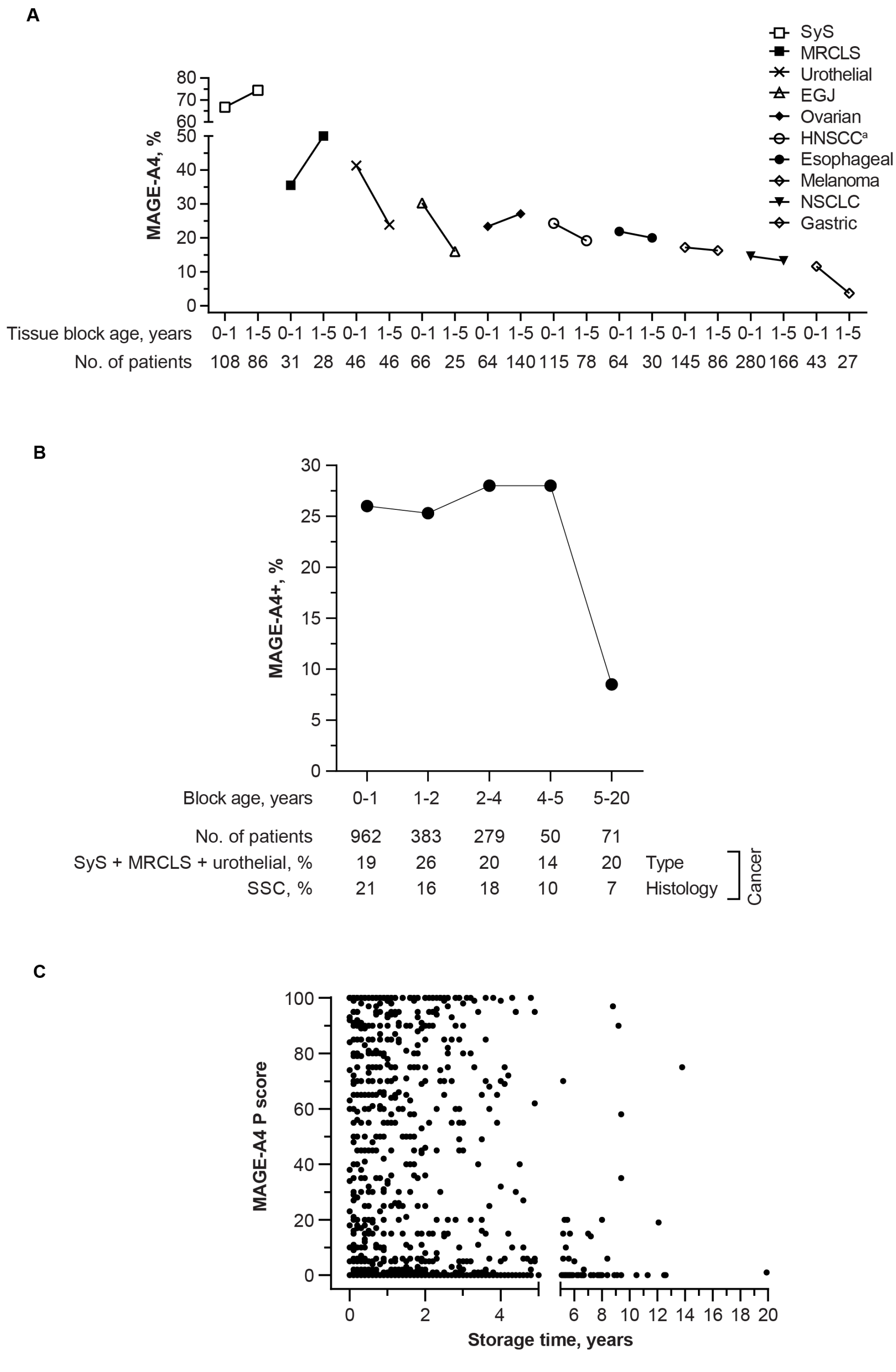

Figure S18. Effect of FFPE tumor sample storage time on MAGE-A4 positivity across cancer types.

(A) Storage time on MAGE-A4 positivity for individual cancer types. (B) Block age on MAGE-A4 positivity for all samples overall. (C) FFPE storage time on MAGE-A4 P score for all samples. [a]n = 199 HNSCC, n = 1 “other” head and neck cancer histology. EGJ, esophagogastric junction cancer; FFPE, formalin-fixed, paraffin-embedded; HNSCC, head and neck squamous cell carcinoma; MAGE-A4, melanoma-associated antigen A4; MRCLS, myxoid/round cell liposarcoma; NSCLC, non-small cell lung cancer; P score, protein score; SCC, squamous cell carcinoma; SyS, synovial sarcoma.
